# Open-source micro-tensile testers via additive manufacturing for the mechanical characterization of thin films and papers

**DOI:** 10.1371/journal.pone.0197999

**Published:** 2018-05-29

**Authors:** Krishanu Nandy, David W. Collinson, Charlie M. Scheftic, L. Catherine Brinson

**Affiliations:** 1 Department of Mechanical Engineering, Northwestern University, Evanston, Illinois, United States of America; 2 Department of Material Science and Engineering, Northwestern University, Evanston, Illinois, United States of America; Dalhousie University, CANADA

## Abstract

The cost of specialized scientific equipment can be high and with limited funding resources, researchers and students are often unable to access or purchase the ideal equipment for their projects. In the fields of materials science and mechanical engineering, fundamental equipment such as tensile testing devices can cost tens to hundreds of thousands of dollars. While a research lab often has access to a large-scale testing machine suitable for conventional samples, loading devices for meso- and micro-scale samples for in-situ testing with the myriad of microscopy tools are often hard to source and cost prohibitive. Open-source software has allowed for great strides in the reduction of costs associated with software development and open-source hardware and additive manufacturing have the potential to similarly reduce the costs of scientific equipment and increase the accessibility of scientific research. To investigate the feasibility of open-source hardware, a micro-tensile tester was designed with a freely accessible computer-aided design package and manufactured with a desktop 3D-printer and off-the-shelf components. To our knowledge this is one of the first demonstrations of a tensile tester with additively manufactured components for scientific research. The capabilities of the tensile tester were demonstrated by investigating the mechanical properties of Graphene Oxide (GO) paper and thin films. A 3D printed tensile tester was successfully used in conjunction with an atomic force microscope to provide one of the first quantitative measurements of GO thin film buckling under compression. The tensile tester was also used in conjunction with an atomic force microscope to observe the change in surface topology of a GO paper in response to increasing tensile strain. No significant change in surface topology was observed in contrast to prior hypotheses from the literature. Based on this result obtained with the new open source tensile stage we propose an alternative hypothesis we term ‘superlamellae consolidation’ to explain the initial deformation of GO paper. The additively manufactured tensile tester tested represents cost savings of >99% compared to commercial solutions in its class and offers simple customization. However, continued development is needed for the tensile tester presented here to approach the technical specifications achievable with commercial solutions.

## Introduction

### Additively manufactured laboratory equipment

Tensile testers are one of the most fundamental pieces of equipment in a materials science laboratory for the analysis of the mechanical properties of materials. In particular, tensile testers can be used to conduct quasi-static loading on a range of materials to characterize the deformation response of a test specimen under a variety of conditions. To produce quantitative data precise instrumentation is required so that the load and displacement experienced by a sample is accurately measured. Commercially available tensile testers are often prohibitively expensive for many laboratories and are limited in their scope and flexibility. In situ mechanical testing with a variety of microscopy tools can yield key fundamental insights to material response, yet each instrument can require different dimensions and constraints on a particular loading device, which decreases access for researchers to equipment appropriate for each specific line of inquiry.

This study presents the first steps toward a solution to the prohibitive costs of tensile testing equipment through the development of an inexpensive and highly customizable tensile tester using a combination of additive manufacturing and off-the-shelf components. Additive manufacturing is the typically rapid production of three dimensional objects from a stock material that is melted and printed through a small, precisely controlled print head to build up a three-dimensional (3-D) component in a layer-by-layer process. Additive manufacturing (colloquially known as 3-D printing) is ideal for the manufacture of low volume products due to its ability to produce complicated geometries in a single step that are currently inaccessible by conventional manufacturing methods. While the original intention of additive manufacturing was for rapid prototyping of products before mass manufacture, improvements in the tolerances and the range of materials accessible to additive manufacturing have allowed it to now become a viable option for ‘rapid manufacture’[1]. Printers have become much more accessible with some models becoming cheap enough for enthusiasts to purchase 3D printers for amateur use. The rise of hobbyists using 3D printers has led to a plethora of CAD models and designs online that are freely available for use[2–4]. Along with the growth of the 3D printing community, inexpensive and single-board microcontrollers such as the Arduino^™^ and the Raspberry PI^™^ have made advanced electronic data acquisition and control systems accessible as well.

While developments in accessible 3D printing and microcontrollers has seen widespread use amongst amateur users, these technologies have only seen limited application within the scientific community. Two examples of scientific equipment manufactured with 3D printing include static optical components[5] and supersonic expansion nozzles[6]. However, these are both simple products without moving parts. Micropipettes are a more mechanically complicated example that uses 3D printing to produce components[7]. Recently, there have been several efforts to integrate electronics with 3D printed components to create a 3D printed plastic AFM head as a replacement for a standard AFM head[8] as well as 3D printed microcontrollers as part of a wirelessly controlled syringe pump library[9].

Examples of 3D printed research equipment are relatively sparse within literature, but the examples present are generally freely available for download from online repositories and are representative of a slowly growing library of open-access laboratory hardware. The potential of widely used open source hardware is enormous. Not only could hardware be easily customized by an individual for their specific application, the potential capital required for purchase as well as ongoing maintenance costs could be significantly reduced. Modular, customizable research equipment could be upgraded as desired with custom grips, more sensitive load cells or other features. Currently, laboratory hardware is produced by a small number of companies, leading to high prices for laboratory equipment and parts for even simple pieces of scientific apparatus. Moving towards open-source hardware could drive down the price of scientific research equipment and improve accessibility of research equipment around the world. The concept of accessible science is exemplified by the work of Prakash et al.[10] who has developed tools for scientific research in impoverished regions of the world.

The benefits of using desktop 3D printers include the low capital compared to other manufacturing equipment, low maintenance costs, prevalence of spare parts online and the ability to customize the printer to suit specific needs. These printers are often readily available for access in shops, ‘maker spaces’ and other central, public facilities. Other considerations must be given to the minimum feature size and accuracy of the 3D printer. The capabilities and affordability of FDM printers have seen significant gains in recent years. High-end consumer FDM printers such as the Ultimaker 3 (Ultimaker, Netherlands) and the BCN3D Sigma (BCN3D, Spain) can print at fine resolutions, use multiple extruders at once and print a wide variety of materials. More recently, a wave of affordable FDM printers have emerged from China (e.g. the Creality3D CR). These options provide a large build area and can be a robust option for basic tasks at a much lower price point than the more expensive FDM printers available.

As the library of 3D printable materials increases, the potential range of components that can be successfully printed increases. Engineering polymers such as Polycarbonate and Nylon have slowly been developed to be 3D printable materials. These polymers have excellent material properties that allow them to be used for load bearing applications that more established 3D printable materials such as ABS and PLA cannot withstand. The addition of fillers to 3D printable polymers is another option that may be of use for the development of scientific equipment. Along with providing an increase in strength[11], fillers such as Graphite powder can also allow 3D printable polymers to be conductive[12] with resistivity between 30Ω/cm in the x-y plane of a 3D printed part to 115 Ω/cm along z-axis of the 3D printed part for one brand of conductive PLA.

The tensile tester developed in this study is focused on the experimental analysis of small samples with high compliance and require low loads for deformation. Therefore, polymers used in FDM or desktop stereolithography (SLA) printers are expected to provide appropriate machine stiffness for the low loads expected during operation. While we focus on polymer-based techniques as they are widely accessible, it is noted that additive manufacturing techniques for metals also exist and will become less expensive and more available in the future. They are expected to be particularly useful to produce low compliance 3d printed tensile testers that can test samples with high stiffness.

The following sections outline the development and testing of a 3D printed customizable tensile tester and its application with an AFM to measure the change in surface topology of a buckled Graphene Oxide (GO) thin films and GO paper under axial tension. The observations enabled by the developed tensile tester allows insight into the behavior of GO morphologies. These studies present a step forward towards a 3D printable tensile tester that can be used in conjunction with modern microscopy equipment. The performance of the tensile tester is then considered and compared to existing commercial solutions.

### Graphene oxide (GO) paper

**Fig 1:**
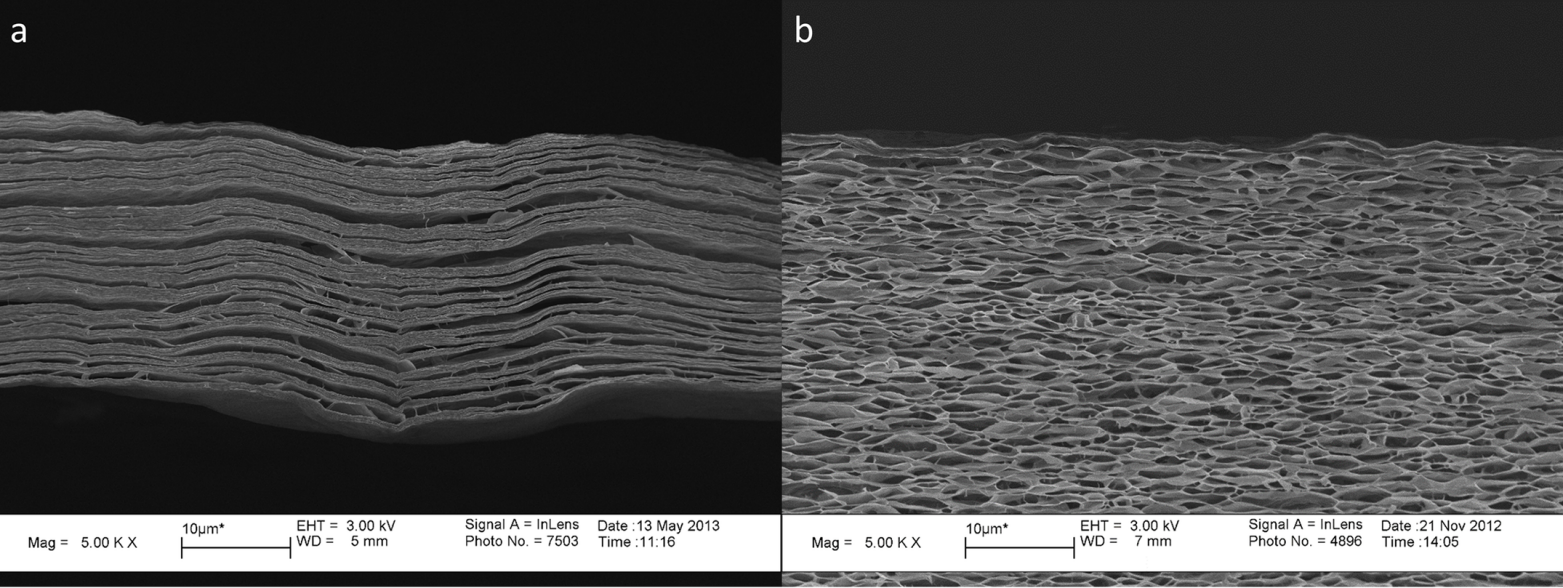
(a) Fully formed GO paper manufactured via vacuum filtration[17] that was swollen and flash frozen with liquid nitrogen before lyophilization to remove the water allowing weak interlamellar regions to delaminate. Weak interlamellar regions near the paper surface are circled in red; (b) GO paper flash-frozen in the final stages of self-assembly (~90%) via vacuum-infiltration to highlight the substructures that exist in GO paper.

To demonstrate the capabilities of each tensile tester, we chose a current material of considerable research interest, graphene oxide films and papers[13–16]. In this section we briefly introduce GO and some features of study regarding its mechanical properties and deformation mechanisms as a paper as well as a thin film. GO Paper is a material with high stiffness, strength and ductility. Previous studies[13, 17] have shown that GO paper possesses a hierarchical structure where nano-sheets (≈5–10 Å thick) organize to form lamellae (≈25 nm thick), each of which is composed of hundreds of nano-sheets. The lamellae also organize to form larger scale super-lamellae (~500 nm thick) which are composed of tens of lamellae. The overall structure forms a paper several microns thick (Fig 1a) with lamellae that are highly interconnected in a honeycomb-like structure as pictured in Fig 1b. The GO papers used in this study are manufactured using a process called vacuum-assisted self-assembly[2, 6, 13] (VASA). The process involves the suspension of GO nano-sheets in an aqueous solution and filtering the solution with the assistance of a vacuum pump to remove the water and create GO paper.

While the mechanical properties of GO paper have been documented[15, 16, 18–20], the mechanism of GO paper deformation under a tensile load is poorly understood, so it desirable to conduct a study where the GO paper is observed in situ as a load is applied. The small length scale of the GO paper substructure requires high resolution microscopy techniques such as atomic force microscopy (AFM) or scanning electron microscopy (SEM) to resolve any change in the GO paper topology as it is deformed. For the sample to be deformed, a tensile tester compatible with these techniques must be developed. A commercial scanning electron microscope (SEM)-compatible tensile tester suitable for GO paper can cost up to USD$27,000 (Deben UK Limited, UK). In this demonstration example, we employ a custom, 3D printed tensile tester in conjunction with atomic force microscopy to explore the in-situ deformation of GO paper samples as produced by Wood et al.[13].

### Buckling of graphene oxide thin films

Buckled films and wrinkled surface have the potential for impact in electronics and optics industries with their ability to alter the interaction between a surface and incident light[21–24]. A study by Thomas et al.[14], presented a mechanical alternative to electrochromic films that allowed for the modulation of visible light by exploiting the delamination buckling of GO films under compression. The transmittance of visible light through GO thin films can be reversibly controlled through cyclic compression and tension from a transparent film (smooth) to an opaque film (crumpled). A theoretical model for buckling of the GO paper during compression was proposed by Thomas et al.[14], but the topology of the buckled films was not measured directly.

**Fig 2:**
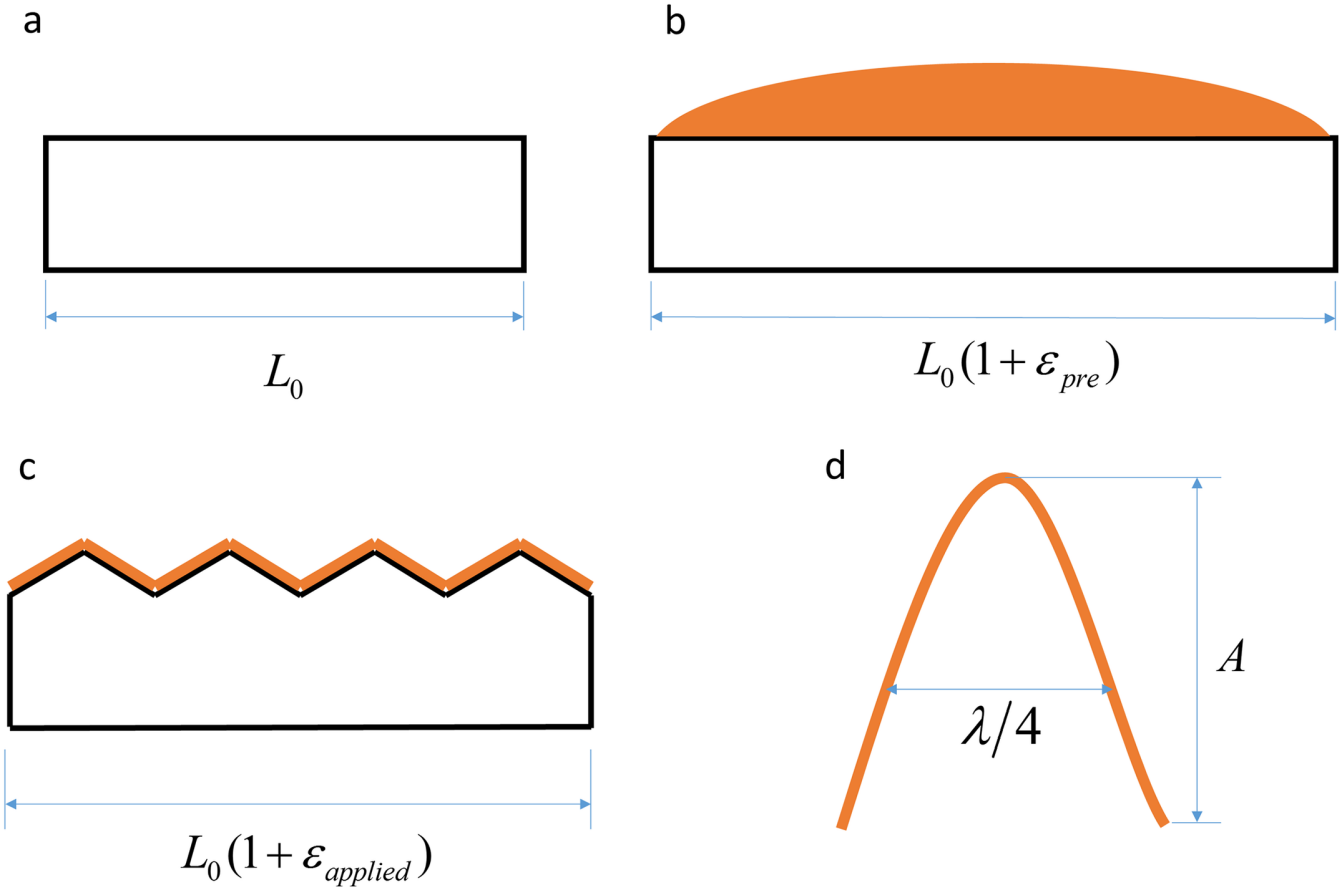
Schematic representation of GO film—PDMS system. (a) Undeformed PDMS film; (b) strained PDMS film with aqueous solution GO drop casted on surface; (c) Buckled GO-PDMS system after partial release of strain in (b); (d) geometry of a single GO buckle due to the release of pre-strain.

In this demonstration, we aim to use an atomic force microscope (AFM) compatible tensile tester to control the crumpling of the GO films and allow an AFM to image the topology of GO thin films deposited on a stretched PDMS substrate and attain quantitative data of buckle topology at various applied strains (*ε*_*applied*_). To make the samples, a PDMS substrate (Fig 2a) with a known gauge length (*L*_0_) is stretched to a known pre-strain (*ε*_*pre*_) and an aqueous GO solution is drop cast onto the substrate (Fig 2b). The strain is then released to allow the GO film to buckle (Fig 2c), forming mesoscale wrinkles (Fig 2d) with an amplitude (*A*) and wavelength(*λ*).

Mechanical models developed by Jiang et al.[25] describes the geometry of continuous, sinusoidal wrinkles developing as the strain is released from a given pre-strain. For buckling to be induced in the GO thin film the pre-strain must be greater than the critical strain (Eq (1)).

$$\varepsilon_{c}=\frac{1}{4}{\left(3{\bar{E}}_{s}/{\bar{E}}_{f}\right)}^{2/3}$$ (1)

Here ${\bar{E}}_{s}$ is the plane modulus of the PDMS substrate, ${\bar{E}}_{f}$ is the plane modulus of the GO film. For a film of thickness *h*, the initial buckle wavelength is

$$\lambda_{0}=2\pi h{\left(\frac{{\bar{E}}_{f}}{{\bar{E}}_{s}}\right)}^{1/3}$$ (2)

As the pre-strain is released, the wavelength of the wrinkle can be predicted by

$$\lambda =\frac{\lambda_{0}(1+\varepsilon_{applied})}{(1+\varepsilon_{pre}){\left(1+\varepsilon_{applied}+\xi \right)}^{1/3}}$$ (3)

As the strain is released where *λ*_0_ is defined in Eq (2). *ξ* is defined as *ξ* = 5(*ε*_*pre*_−*ε*_*applied*_)(1+*ε*_*pre*_)/32 which is included to account for geometric and constitutive nonlinearities. The wrinkle amplitude is defined as

$$A=h\frac{\sqrt{(\varepsilon_{pre}-\varepsilon_{applied})/\varepsilon_{c}-1}}{\sqrt{1+\varepsilon_{pre}}{\left(1+\varepsilon_{applied}+\xi \right)}^{1/3}}$$ (4)

For the purposes of this study, the following values were used for the for the substrate and film material properties:${\bar{E}}_{s}=280\;\text{kPa},\;\nu_{s}=0.48,\;{\bar{E}}_{f}=28\;\text{GPa},\;\nu_{f}=0.3$. ${\bar{E}}_{s}$ was determined experimentally, the other material parameters were extracted from literature[25].

## Methods

The development of a custom, additively manufactured tensile tester and application to several studies is detailed in this section. Engineering details of the tensile tester and its manufacture are presented Followed by the required steps for the sample preparation of buckled GO thin films and GO paper. The steps taken for AFM and SEM analysis of the GO paper and the buckled thin films is also given.

### 3D printed AFM compatible tensile tester

**Fig 3:**
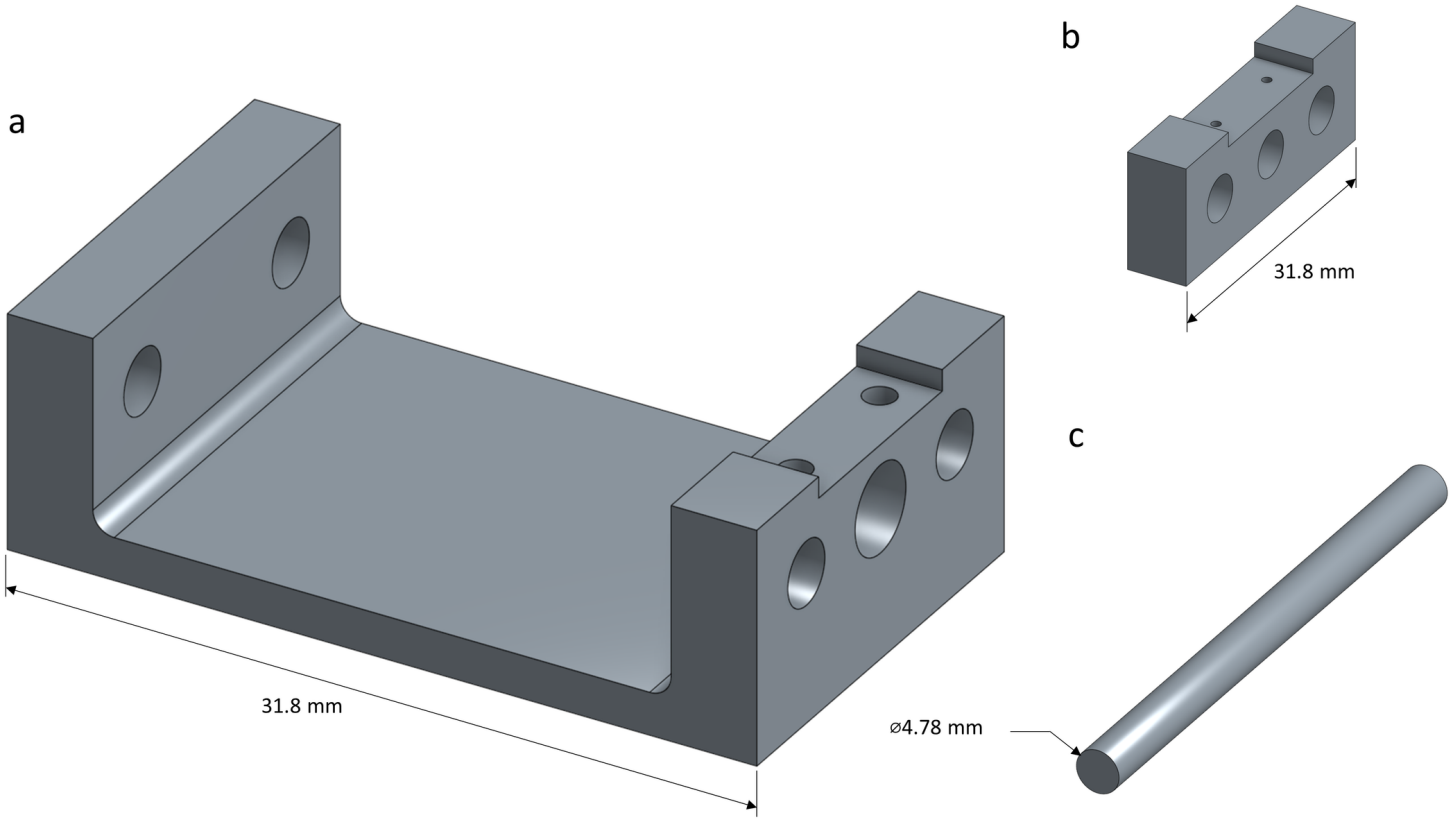
Solid CAD models of AFM tensile tester (AMT1) components manufactured using additive manufacturing. (a) Frame. (b) Sample mount. (c) Brace.

**Fig 4:**
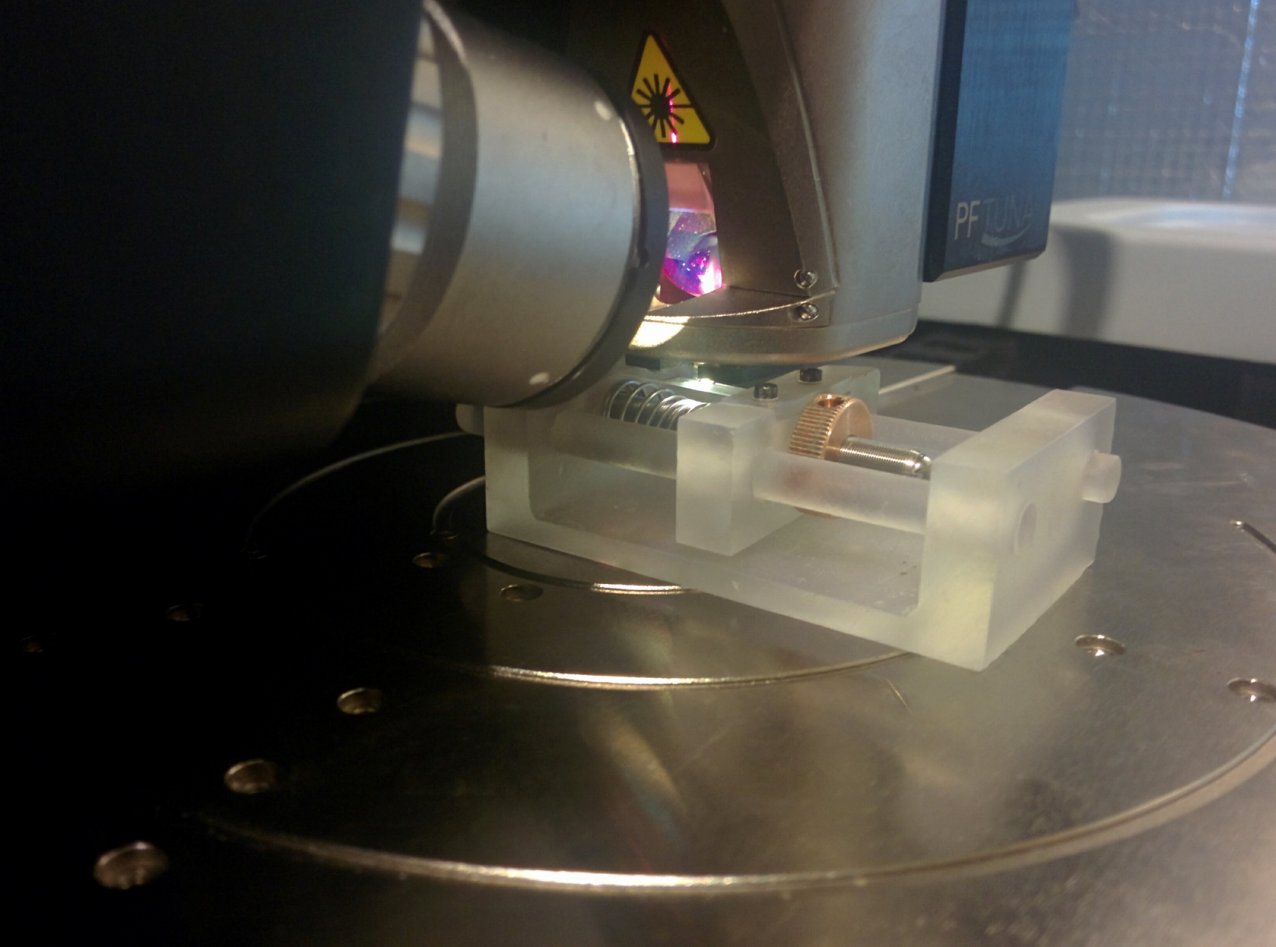
AFM compatible tensile tester (AMT1) in-situ under a Bruker Dimension Icon^™^.

A tensile tester (Fig 3) termed ‘AMT1’ was additively manufactured using stereo lithography (SLA). The structural components of the tester were produced with a desktop photopolymer printer (FormLabs, USA). The form factor of the tensile tester was designed so that it could fit under a Bruker Dimension Icon AFM (Fig 4). The developed system allows for materials to be tested under an AFM while being held at a static strain. Engineering drawings in S1 Drawing detail the dimensions and design of the additively manufactured components of AMT1 and include a parts list of all the required components. The computer models and the engineering drawings for AMT1 are also accessible online at Onshape^™^[26].

### Materials

#### GO films on stretched PDMS substrate

To prepare graphene oxide solutions GO was dispersed in de-ionized water and diluted to a concentration of 0.25*mg*/*mL*. Then the solution was sonicated in a water bath for 30 minutes to encourage homogenous dispersion of GO particles. The GO solution was then diluted further to concentrations between 0.0025–0.01*mg*/*mL*, based on the film thickness desired in the final sample. The diluted solutions were then centrifuged for 30 min to remove large GO particles and impurities. Approximately 50*μL* of the diluted solution was drop-cast onto a PDMS substrate that was typically 5mm wide and 21 mm long. To prepare the substrate, it was rinsed in de-ionized water and dried using pressurized air. The PDMS substrate was then mounted in the tensile tester and a pre-strain was induced. The GO solution was then deposited, and the solution was left to slowly dry overnight in a dust-free environment.

#### Atomic force microscopy imaging and analysis

The topography of both sets of composites were measured via a Bruker Dimension Icon^™^ in tapping mode to measure the surface topology of the dried GO films and GO paper. Silicon cantilevers (RTESPA-150, Bruker, USA) with a nominal stiffness of 6N/m were and nominal probe radius of 8 nm were used. The mounted samples were systematically scanned and then rescanned at different levels of applied strain so that the development of the GO film or GO paper topology as a function of strain could be observed. The images captured at each level of applied strain were then analyzed with to determine the development of surface topology as a function of strain.

#### Scanning electron microscopy imaging

To confirm the GO paper topology observations conducted by the AFM a complementary study was conducted in a SEM with a conventionally manufactured tensile tester. The GO paper sample placed in the SEM was a strip approximately 2mm wide with a gauge length of 25mm. The sample was first secured in the tensile tester and then entire structure was coated in 8 nm of Osmium by an Osmium coater (Structure Probe Inc., West Chester, PA) to render the non-conductive GO paper sample conductive. The whole apparatus was mounted in a FEI Nova NanoSEM 600 (FEI Company, Hillsboro, OR). The sample was then imaged without any strain. Next the vacuum was released, and the sample was removed from the chamber and placed under a tensile load to induce a strain of 2.7% before being returned to the SEM and imaged again. The apparatus was removed one final time and the sample was strained until failure and imaged a third time by the SEM. The images were taken at various magnifications and compiled to observe any topographical change in the GO paper sample because of the strain.

## Results

The following section presents and discusses the experimental results of the demonstration problems and the performance of AMT1. The first experiment examined the surface through AFM of a GO paper held in the AMT1 at increasing levels of tensile strain until the paper failed. The experiment was then repeated with a conventional tensile tester and a SEM to validate the results. The second experiment used the AMT1 tensile tester to compress a GO thin film to observe the formation of buckles in the GO paper using AFM under a compressive strain. AMT1 was successfully used to produce experimental data and allow conclusions to be drawn about the mechanical behavior of GO paper and thin films. Future iterations of the devices will refine the tolerances and mechanical stiffness of the systems to improve the accuracy of the experimental data produced.

### GO paper under static strain in the AFM

**Fig 5:**
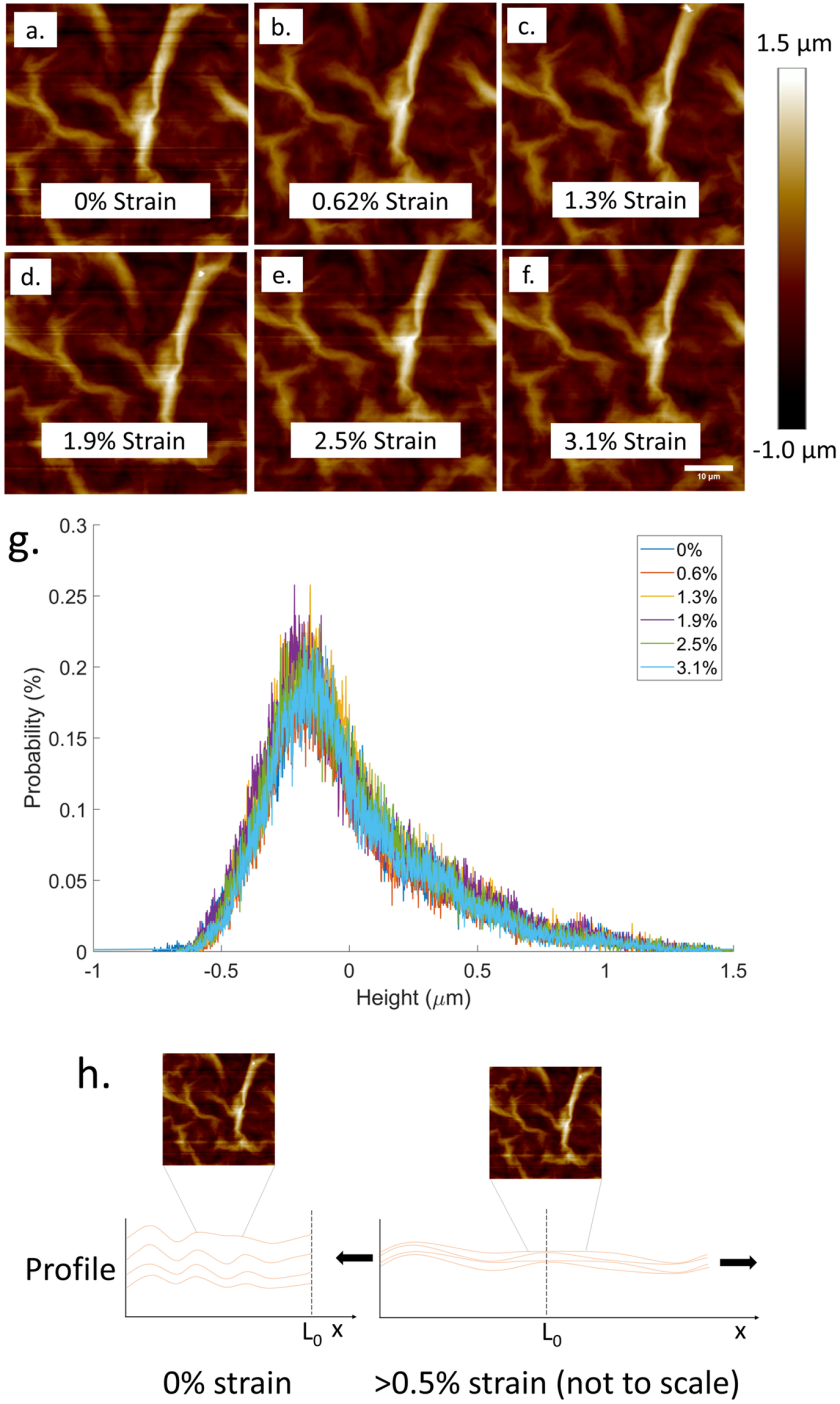
Height topography of a GO paper at (a) 0% strain, (b) 0.62% strain, (c) 1.3% strain, (d) 1.9% strain, (e) 2.5% strain, (f) 3.1% strain; (g) Spectral distribution of measured height at increasing amounts of strain for all pixels in each image(a-f). (h) Schematic of super-lamellae consolidation mechanism as a GO paper sample is loaded (not to scale). All AFM images are 25x25μm.

**Fig 6:**
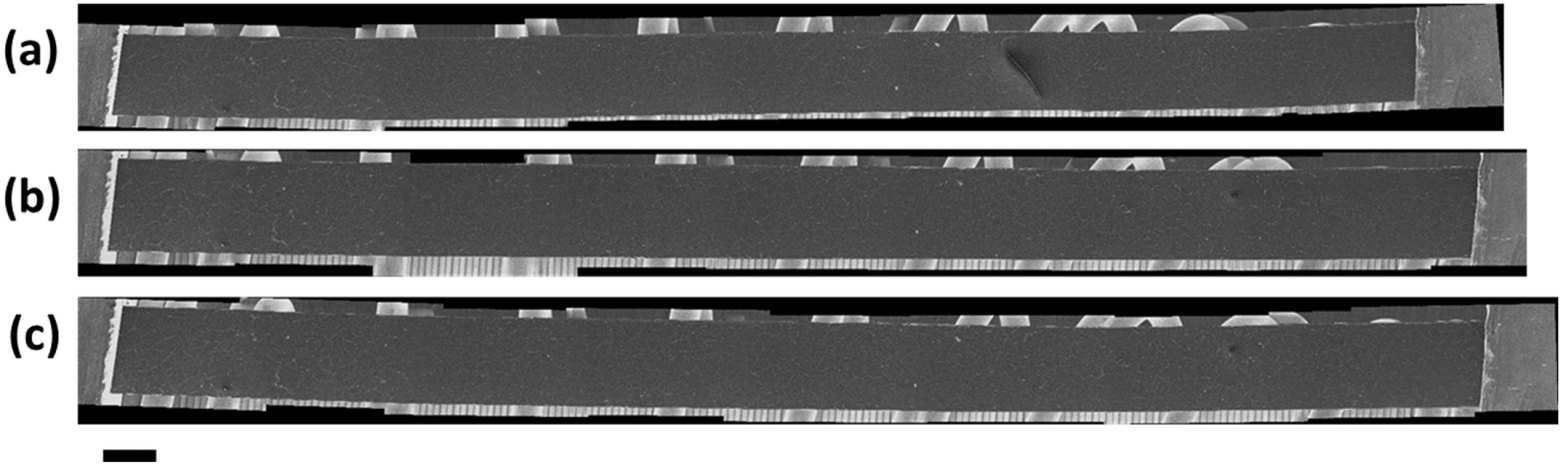
Composite SEM images of GO paper mounted on the tensile tester in-situ. (a) Zero-strain condition. The feature indicated by the red circle in is dust (b) 2.7% strain (c) Post-fracture condition. Fracture occurred underneath the RHS grip.

**Fig 7:**
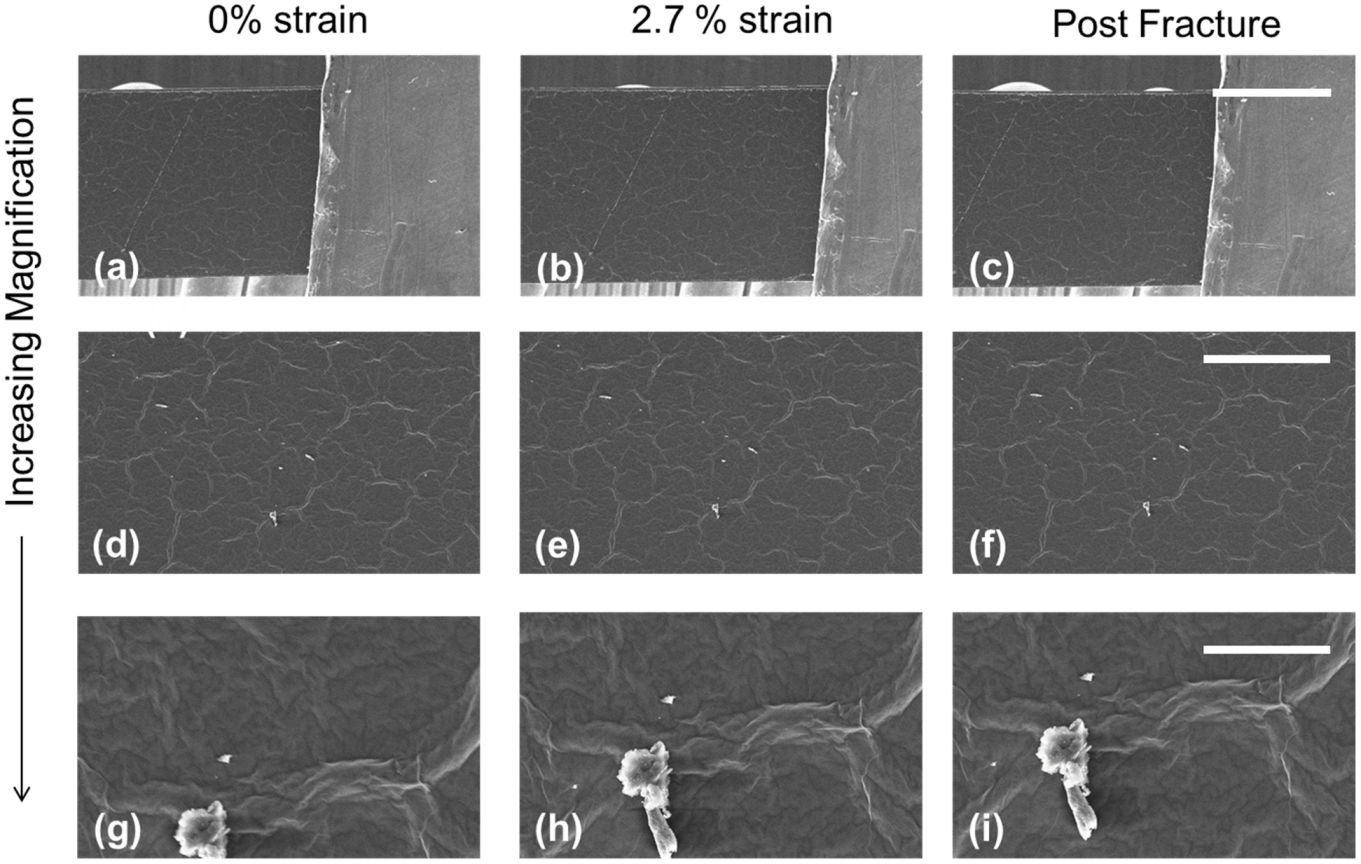
SEM images of GO paper at 0% strain (a, d, g), 2.7% strain (b, e, h) and post fracture (c, f, i). The light gray feature that can be observed on the right-hand side of figures a, b and c is the metallic grips of the tensile tester. The bright irregular features in figures g, h, i is dust on the surface of the GO paper.

Tapping mode AFM was used to capture the surface topology across the surface of a GO paper sample at increasing levels of strain (Fig 5). The collapse and relaxation of surface wrinkles has been posited in previous studies [15, 20] as a possible mechanism for the initial ‘yield’ of GO paper under tensile strain. However, the topographical data presented provides evidence to suggest that this hypothesis is incorrect. To compare to the AFM data, a GO paper sample was mounted in the SEM-compatible tensile tester and then visualized with SEM, as presented in Fig 6. The images in Fig 6 are constructed by stitching multiple SEM images together to create a macroscale overview of the mounted sample at various states. Higher magnification images of the sample are presented in Fig 7 to look for deformation on the surface of the GO paper.

Neither the SEM images (Fig 7) nor the AFM scans (Fig 5a–5f), show discernible change in features: surface wrinkles of the GO paper sample do not show any clear qualitative change in width or height as the sample displacement is increased. These observations are consistent with the data presented in Fig 5g as the spectral distribution of the recorded height in each image did not significantly change as the sample displacement increased.

The images in Fig 7(d)–7(f) were binarized and then the centroid location and extent of each surface wrinkle were compared between each image. Again, no apparent strain was observed and no significant increase in distance between surface features was observed as the sample displacement increased. The evidence suggests that the previously hypothesized wrinkle collapse does not occur in GO paper due to tensile load.

These experimental results were consistent across multiple GO papers and suggest that alternative mechanisms for the initial mechanical behavior of GO paper should be considered. Based on the observations provided by these in situ tests, ‘superlamellae consolidation’ (Fig 5h) is proposed as a potential mechanism for the initial elastic deformation. A tensile load applied to a GO paper sample causes the super-lamellae (Fig 5h) to straighten and as a result compress the space between the super-lamellae layers. Any change in topography because of super-lamellae consolidation is suggested to occur at a length scale larger than what was imaged for this study. Therefore, the locally constrained wrinkles dominate the spectral height distribution of the captured images and no significant change between the images because of applied strain was observed. The ‘super-lamellae consolidation’ is also suggested to occur internally and would not readily observable by AFM or SEM surface images, consistent with results here. Consequently, the wrinkles typically observed on GO papers (Figs 7 and 5a–5f) are suggested to be features that are highly constrained locally and therefore insensitive to deformation under strains applied globally to the GO paper. It is suggested that future experiments be conducted with a GO paper cross-section under observation to confirm or disprove that the super-lamellae geometry compresses and elongates because of tensile loads.

### Experimental measurement of GO thin film buckling

To further explore capabilities of AMT1, buckling of two GO films supported on PDMS (Section 3.2.1) was examined under the AFM using AMT1. The first sample was a thick film (≈ 250*nm*) pre-strained to 236%. The second GO film was a thin film (≈ 50*nm*) pre-strained to 246%. Strain was released in small increments by reducing the gauge length approximately ~60μm at a time so the initial buckle formation could be captured with the AFM. Once the images of the initial buckle formation were captured, the gauge length was systemically reduced approximately 250 μm to capture the continued growth of the buckles. The extracted topology from the AFM provides a direct, in-situ measurement of GO film topology that can be used to confirm theoretical models of thin film buckle development as a function of applied strain.

It was observed that the amplitude and wavelength of the buckles varied between different regions in the GO film. The variance observed is thought to be the result of varied adhesion between the PDMS substrate and the GO films which would impact the transfer of strain from the substrate to the GO thin film. The areas of the film in better contact with the substrate surface will experience a greater transfer of strain from the PDMS substrate. As the buckle amplitude and wavelength is dependent on the applied strain[25], any variance in adhesion will lead to variance in the size of the buckles throughout a sample. Perfect adhesion is an assumption made by the buckling mechanics model used[25], and the model will not perfectly predict buckle development in locations where poor adhesion exists between the substrate and film. Therefore, the application of the buckling mechanics model was adjusted to account for poor adhesion by using the film thickness (*h*) as a fitting parameter. The GO thin film buckling behavior provides a platform to demonstrate that AMT1 can successfully be used to capture high-quality nanoscale images of a strained sample in conjunction with an AFM.

**Fig 8:**
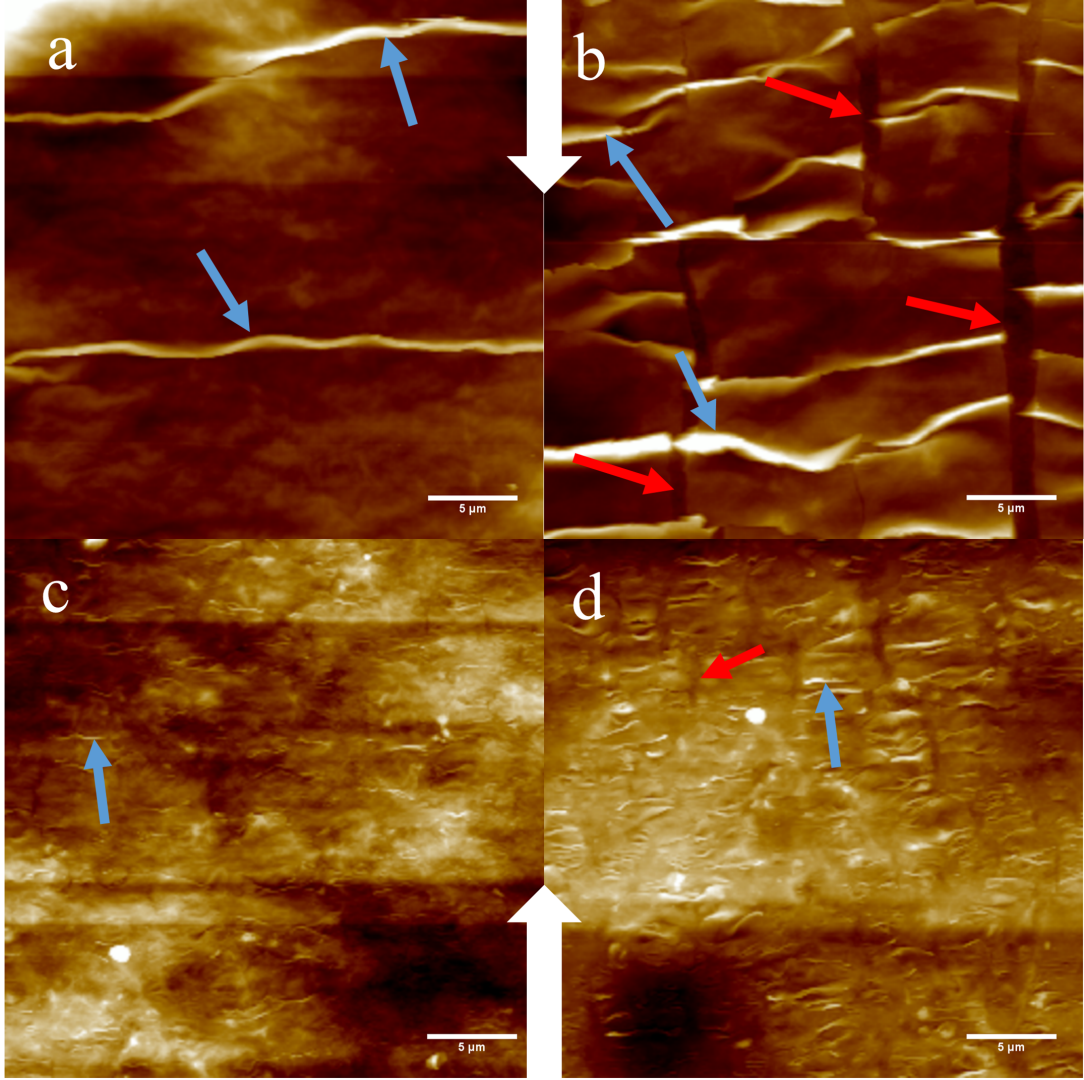
AFM height images of buckled GO film deposited from 0.01 mg/mL (a and b, “thick” films) and 0.0025mg/mL solution (c and d, “thin” films). (a) 187% applied strain; (b) 159% applied strain;(c) 187% applied strain;(d) 159% applied strain. Blue arrows indicate buckles and red arrows indicate transverse cracking. The large white arrows indicate the direction of the compressive strain applied to the thin films for all the images.

Fig 8 displays several acquired AFM images of the buckled GO films using the AMT1. Fig 8(a) shows surface topology of a thick GO film (≈ 250*nm*) after the initial pre-strain of 236% was reduced to 187%, Fig 8b is the same film after the induced strain was released further to 159%. In both images, the developing buckles are on the order of 1–2 *μm* in height. Fig 8c and 8d are images of a thinner GO film (≈ 50*nm*) at an applied strain of 187% (Fig 8c) and 159% (Fig 8d). In the thinner films the buckle height was on the order of 100-500nm. Eq (4) predicts that the buckle amplitude is proportional to the film thickness and this is consistent with what is observed in Fig 8 with the buckling amplitude in the thinner film (Fig 8c and 8d) less than that observed in the thicker film (Fig 8a and 8b). Transverse cracking was observed in the films because of induced transverse strain.

The theoretical models (Eqs (3) and (4)) used to predict the buckle wavelength (*λ*) and amplitude (*A*) assume a continuous sinusoidal pattern and perfect adhesion. In contrast, these experimental samples are expected to have variabilities in adhesion between the GO and the PDMS, surface imperfections and film thickness that will produce a non-uniform buckling pattern. The transverse cracks are also expected to affect the continuity of the buckling pattern. However, several trends observed are consistent with the theoretical predictions that thicker films produce larger buckles than the thinner films, and initial onset of buckling occurs at the expected critical strain (*ε*_*c*_). A future study focusing on the mechanics of GO film buckling may either apply an additional surface treatment to improve adhesion or develop current buckling mechanics models to adjust for imperfect adhesion.

The induced strain in the PDMS was controlled by adjusting the gauge length in increments as small as ≈ 65*μm*. No obvious drift in gauge length was observed while the drop-cast GO solution dried over a period of ~12 hours or during imaging, and high-quality AFM images were captured that exhibited no drift. The biggest limitation of the device in its current iteration is that the sample mount of AMT1 (Fig 3b) contacts the AFM head at low gauge lengths. While not required for the experiments conducted here, the frame of AMT1 could be modified in future iterations of the tensile tester so that the sample mount passes underneath the AFM head and this feature would be easily customizable for different constraints for any model of AFM.

**Fig 9:**
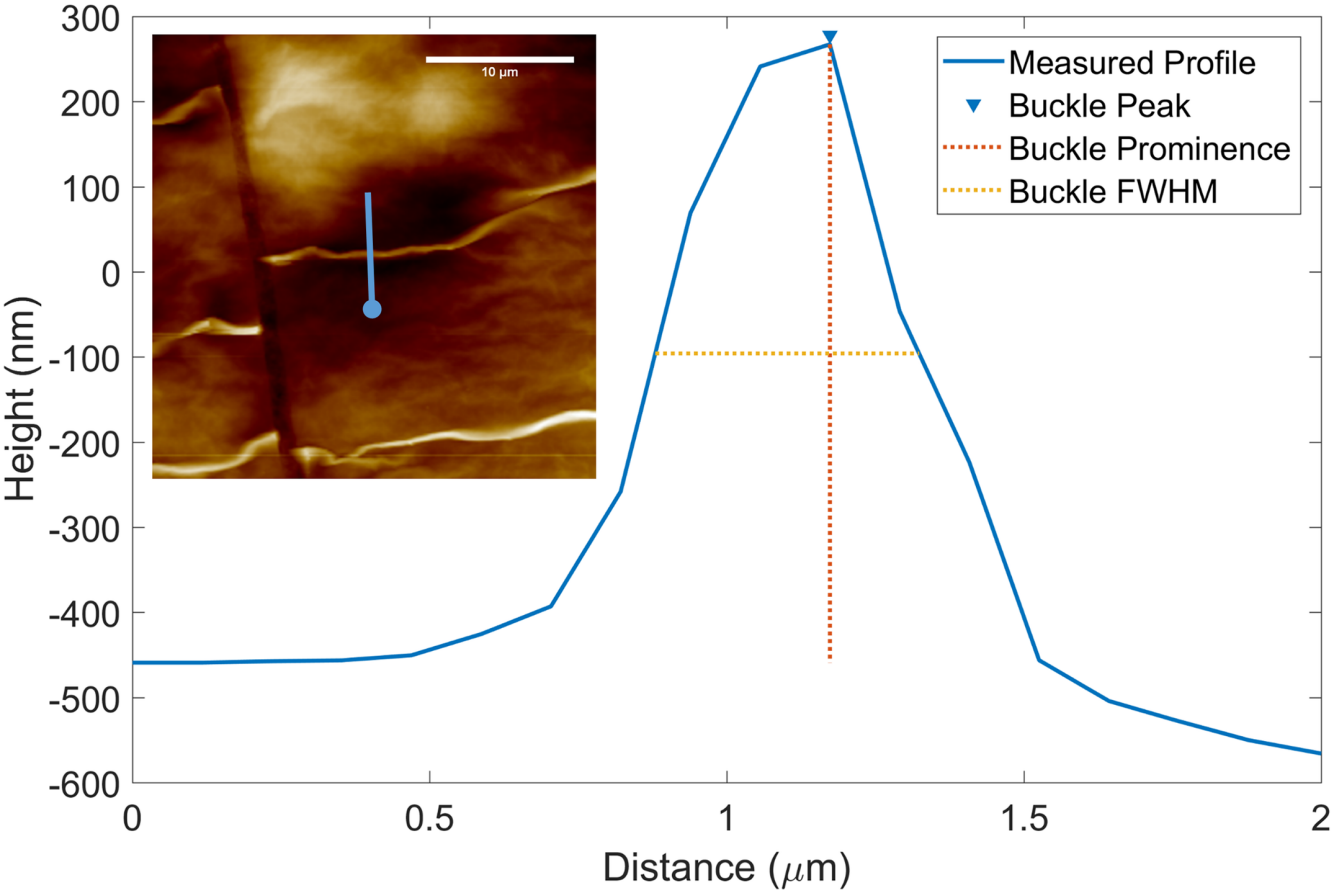
Experimental profile and characterization of a representative buckle observed in the thick (≈ 200*nm*) GO film at 214% applied strain; Inset. AFM image depicting location of line scan used to measure buckle profile. The blue line indicates line scan orientation and the blue circle indicates the line scan origin.

Further detailed imaging can be captured with the AFM using AMT1 to compare topographical data to the models presented earlier. Line scans are taken across the buckles in the AFM scans to produce a profile of the buckles. The amplitude and full width—half-maximum (FWHM) of a buckle profile is then measured as shown in Fig 9. The amplitude (*A*) of the buckle is equivalent to the buckle prominence and the buckle wavelength (*λ*) is approximated to be twice the measured FWHM. From the topology captured (Fig 8) it is evident that the GO-PDMS system does not buckle in a purely sinusoidal pattern with the distance between each buckle significantly greater than the FWHM of each buckle. This may lead to disagreement with theoretical predictions, which assumes a perfect sinusoidal pattern of buckling, but may still allow for a qualitative comparison between the theoretical prediction how the buckles develop and the experimental data.

**Fig 10:**
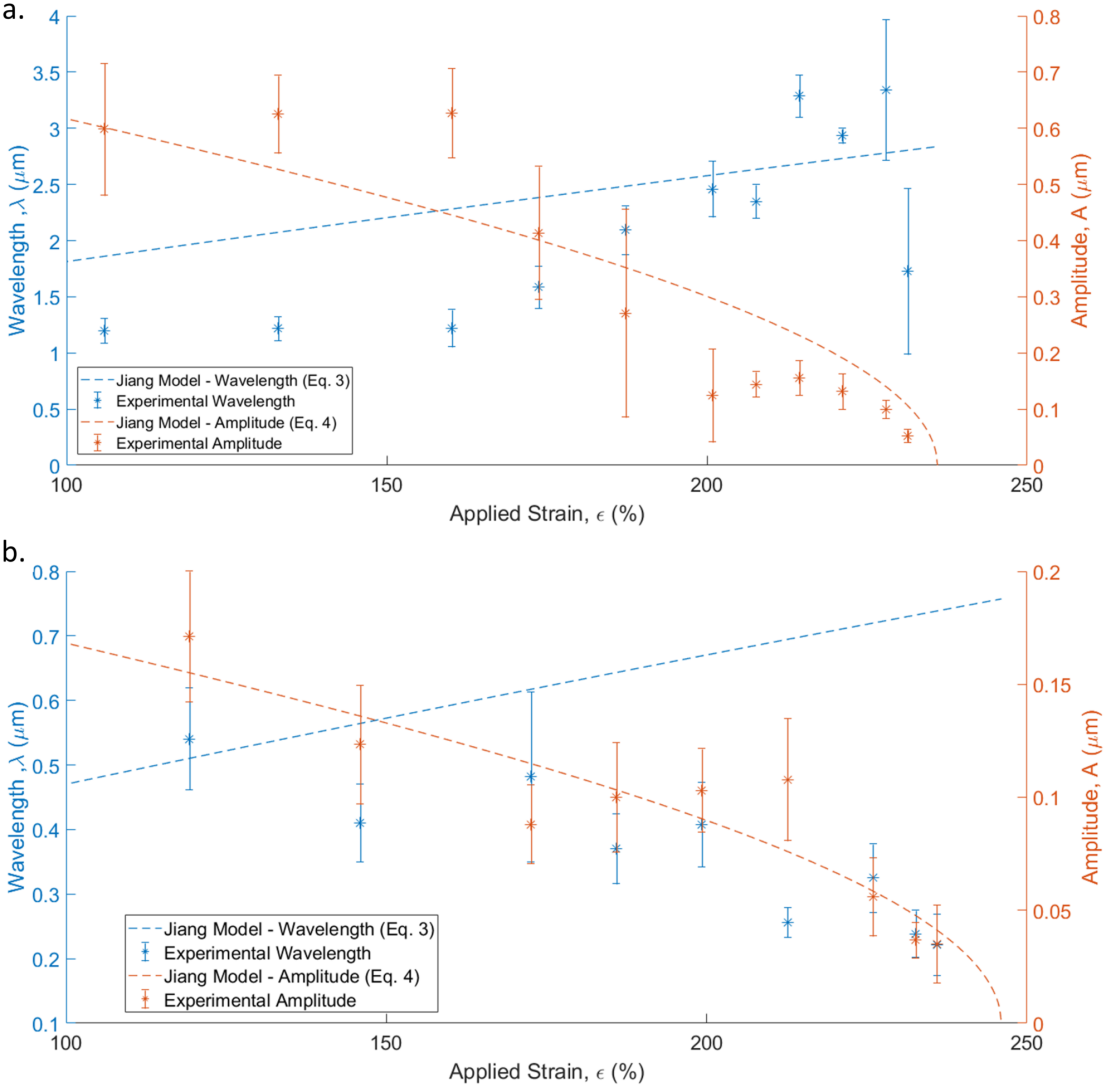
(a) Experimental data and predicted trend lines for buckle amplitude and wavelength induced in thick film (~250nm) deposited from a 0.01mg/mL solution of GO. Each data point is generated from multiple height profiles across 2–6 buckles observed at each applied strain. The error bar indicates the standard error of the measured amplitude and wavelength data. (b) Experimental data and predicted trend lines for buckle amplitude and wavelength induced in a thin film (~50 nm) deposited from a 0.0025mg/mL solution of GO. Each data point is generated from height profiles across 2–9 buckles observed at each applied strain. The error bar indicates the standard error of the measured amplitude and wavelength data.

The experimental results, considering data from multiple buckles at each strain level, are compared for the thick film deposited from a 0.01mg/mL solution (Fig 8a and 8b)) to the buckling mechanics model (Eqs (3) and (4)) in Fig 10a. The model was fit to the experimental data by using the film thickness (*h*) as a fitting parameter. The fitted film thicknesses used were lower than the experimentally measured thickness (*h*_*fit*_ ≈ 21*nm* for the 250nm film, *h*_*fit*_ ≈ 6*nm* for the 50nm film), suggesting that the buckle development as a function of applied strain was not as significant as predicted. Therefore, it is proposed that the scans were taken in regions of low adhesion which resulted in a poor transfer of strain and a reduced response to applied strain compared to theory. Fig 10b compares the experimental data to the mechanics model for a thinner film deposited from a 0.0025*mg*/*mL* solution. Again, the film thickness was used as a fitting parameter. While the data is in reasonable agreement to the model for the thicker film, for the thinner film the increase in buckle wavelength as the applied strain decreases conflicts with the prediction made by the mechanics model.

The error bars that can be observed in Fig 10a and 10b are the result of variance between the buckles examined as part of the analysis as well as variance in the height and width of a single buckle. Measurements were made on buckles for within the same area of the film as the applied strain decreased, however the location of the profile measurement (Fig 9) varied. Future iterations of this experiment could focus on the development of a single buckle to reduce variance or seek methods to attain consistent adhesion between the GO film and the PDMS substrate.

## Discussion

The two studies presented here provide one of the first demonstrations that cost-effective, additively manufactured tensile testers can be used conjunction with qualitative and quantitative microscopy techniques enabling new findings. Specifically, in the studies here, conclusions can be drawn about the response of GO paper and thin films to uniaxial strains. The flexibility and low cost of AMT1 and the ability to iterate its design to be compatible with a specific microscopy instrument is a significant advantage over commercial options. Future meso-scale characterization of thin films and fibers, which are important classes of materials, require load cell accuracy that is often below what commercially available tensile testers can provide without great expense. While the current iteration of the 3D-printed tensile tester does not compete with the control achievable with the commercial options, it represents the potential for enormous cost savings and a significant advantage in terms of customizability. An additively manufactured tensile tester can be developed for a researcher’s specific constraints without major capital expenditure.

In future work, topology analysis could be improved by techniques such as digital image correlation (DIC) to quantify any change in surface structure. However, the use of DIC for this purpose is complicated by the change in sample orientation between images as well as the distortions that occur during SEM and AFM image capture[27, 28]. The use of DIC with the AFM is also additionally complicated due to the changes in the location of the scan window as the sample displacement is increased and artifacts that appear in the image during scanning. However, DIC has been used in conjunction with an AFM in a study conducted by Chasiotis and Knauss[28] and was able to resolve strain with a 0.04% resolution on the surface of a polysilicon dog bone. This technique may be suitable for future studies conducted with GO paper, but development would be needed to operate on samples more inhomogeneous than the polysilicon in that study. Future iterations of the conventional tensile tester could incorporate a load cell and DIC to allow for the generation of a stress-strain curve during image capture on the SEM.

As an alternative to DIC, the IDEAL lab has developed stochastic methods [29–32] that could be applied to the images presented in Figs 7a–7i and 5a–5f to characterize the microstructure. Any significant difference between the surface features due to applied strain could be determined without use of DIC, avoiding image distortions complications. However, this approach does not determine local strain fields explicitly, and instead provides quantitative statistical measures of microstructural changes.

To understand how AMT1 relates to commercial micro tensile testers, a list of commercial solutions for straining GO paper is compiled in Table 1. Each option was identified to be suitable for application based on the maximum capable load required to cause failure in our samples of GO paper (>5N), and a maximum sample length of at least 15mm. The commercial options presented here typically include the ability to directly measure the load applied across the sample which is a distinct advantage. Initial development work not presented here suggests that load sensing capability can be included with an updated AMT1 without significant additional cost or complexity.

**Table 1:** Comparison between commercially available options and AMT1 developed in this study.

Model	Load Capacity	Displacement Resolution	Displacement rate	Gauge range	Cost
AMT1	-	64μm	-	8-25mm	$12.04 ^a^^,^^b^
Deben Microtest	200 N	5μm	1.7–25μm/s	10–35 mm	$27,000
Kammrath Weiss	100 N	-	0.3–50 μm/s	80 mm	$62,000
Asylum NanoRack	80N	5μm	-	41-161mm	$26,500
AMT1 w/ piezo drive	10 N	1nm		30 mm	$4101^c^

NOTES:

^a^The estimated cost of the 3D printed tensile tester considers the cost of the PLA (USD$0.053 per gram (MakerBot^®^ Industries, USA)), the electrical power consumption per gram of printed material (12.43 cents/kWh (http://www.eia.gov/electricity/data/browser/#/topic) with ~0.007kWh per gram of printed material consumed by a typical desktop printer[5] and the cost of the actuation and DAQ components.

^b^The commercial prices and specifications were obtained from quotes and website data provided by the manufacturers of their respective tensile testers.

^c^The piezo motor considered is a PI N310^®^ piezo linear actuator and the cost includes the necessary driver.

It was >99.95% cheaper to manufacture the tensile tester in this study than purchase the NanoRack^™^ (Asylum Research, USA), which was designed specifically for the MFP-3D^™^ AFM (Asylum Research, USA). Even if future iterations of the additively manufactured tensile testers call for higher tolerance components and advanced piezo motor actuation, the relative cost savings compared to commercial solutions are expected to remain approximately 85–93% less (Table 1). Significantly less capital and technical skill is required to produce a functional, custom tensile tester via additive manufacturing than conventional manufacturing techniques. Whereas the conventional methods require multiple machining operations with several pieces of manufacturing equipment, the additively manufactured tensile can produce the required components in a single print cycle.

While a 3D printed tensile tester does represent a major increase in affordability and flexibility compared to commercial options, it should be noted that the 3D printed tensile lacks features and components that commercial options offer which makes a direct price comparison potentially facetious. However, it is believed that with sufficient time and crowdsourced development the deficiencies of a 3D printed tensile tester can be overcome. Websites such as GitHub.com or nanoHUB.org facilitate the sharing of information and could be tools to accelerate the development of additively manufactured scientific equipment through crowd sourcing of original designs as well as improvements on existing designs submitted to these shared online resources. On a broader scale, this represents an enormous opportunity for research scientists to produce their own scientific equipment when financial constraints or customization make purchase of commercial alternatives impractical.

## Conclusion

This study developed an open-source, additively manufactured tensile tester (termed ‘AMT1’); the first example of an additively manufactured tensile tester developed to be used in conjunction with microscopy techniques. In conjunction with advanced microscopy, the utility of AMT1 was demonstrated through examination of meso-scale Graphene Oxide (GO) thin films and papers. The concepts developed here provides an exemplar for how additive manufacturing can reduce the cost of scientific investigation for researchers while providing equipment specifically tailored to a desired study that can be >99% cheaper than a commercial solution. The inexpensive pieces of scientific equipment developed for the studies could provide valuable data and insight for understanding the material response to strain, even with some limitations on accuracy and control. With further development, 3D printed tensile testers hold promise to compete with the accuracy of currently available commercial micro-tensile testers. Access to additively manufactured scientific equipment will allow a much broader audience to make scientific contributions to their fields of interest.

The first demonstration problem aimed to determine the mechanism that produces the elastic response of GO paper to a tensile load. Investigation of GO papers underneath an atomic force microscope suggest that surface wrinkles expected to flatten under tensile load are highly constrained locally and are not affected by low amounts of strain in contrast to the expected behavior proposed in literature. A new hypothesis is proposed to explain the initial mechanical deformation of GO paper. We suggest that internal layers in a GO paper sample collapse and consolidate in response to an in-plane tensile load. For the second demonstration problem, AFM was used for one of the first quantitative characterizations of buckle development in GO thin films as a function of compressive strain. When compared to theoretical predictions, discrepancies in the experimental results suggest poor adhesion between the GO film and the PDMS substrate. Several suggestions for the improvement of the developed tensile testers are provided, including the inclusion of a piezo element for fine motor control and addition of a load cell for force measurement.

## Supporting information

S1 Drawing Engineering drawing and parts list for tensile tester AMT1. (PDF)

S1 Data Raw data files for the two demonstration problems. (ZIP)
