# Supplementary material for: Open-source micro-tensile testers via additive manufacturing for the mechanical characterization of thin films and papers
Source: PLoS One. 2018 May 29;13(5):e0197999. doi: 10.1371/journal.pone.0197999 (PMC5973562; doi:10.1371/journal.pone.0197999)
Supplement: S1 Drawing — (PDF) [file pone.0197999.s001.pdf]

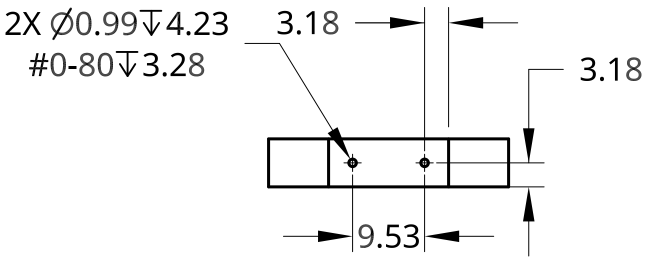

1-SHUTTLE
PLAN VIEW

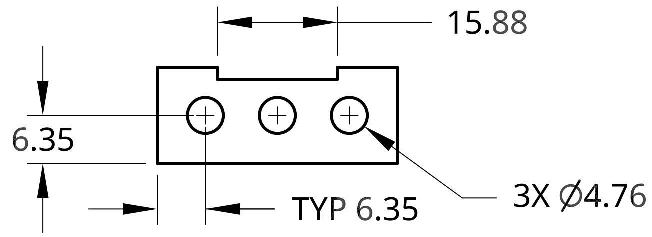

1-SHUTTLE
ELEVATION VIEW

PART NO.	NAME	QTY	DWG
1	FRAME	1	SI-2-2-1
2	SHUTTLE	1	SI-2-1-1
3	CLAMP	2	SI-2-1-2
4	SUPPORT ROD	2	SI-2-1-3
5	COMPRESSION SPRING, 0.3X2in, 2.6lb/in	1	-
6	ULTRA-FINE ADJUSTER, 3/16"-100, 2.0"	1	-
7	BUSHING, 3/16-100, O.D 7/16-UNC	1	-
8	CAP SCREW #0-80, 0.175in	4	-
9	PRESS INSERT, #0-80, O.D. 0.104in	4	-

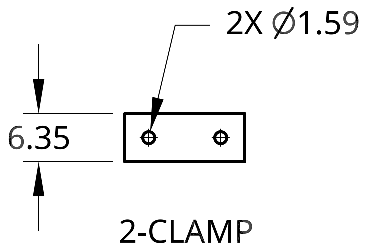

2-CLAMP
PLAN VIEW

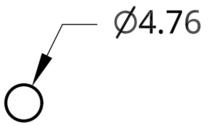

3-SUPPORT ROD
ELEVATION VIEW

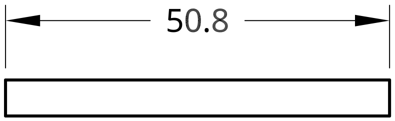

3-SUPPORT ROD
PLAN VIEW

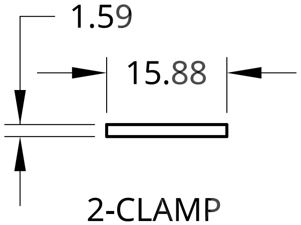

2-CLAMP
PLAN VIEW

UNLESS OTHERWISE SPECIFIED, DIMENSIONS ARE IN MILLIMETERS	SCHEMATIC AND PART LIST
DO NOT SCALE DRAWING<br>BREAK ALL SHARP EDGES AND REMOVE BURRS<br>THIRD ANGLE PROJECTION	TITLE: AMT1 - AFM COMPATIBLE TENSILE TESTER
SIZE: A3	DWG NO.: SI-2-1 — REV.: A
SCALE: 1:1	MATERIAL: PLA — SHEET: 1 of 2

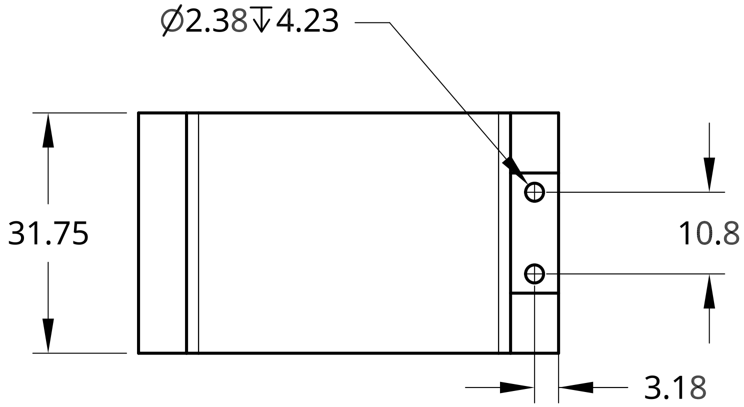

1-FRAME
PLAN VIEW

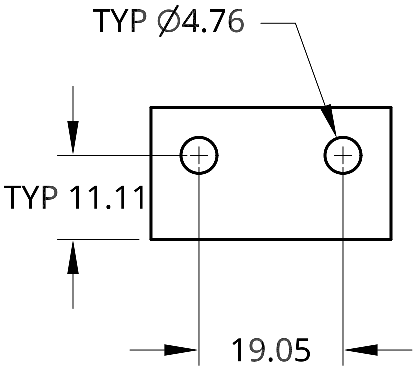

1-FRAME
SIDE VIEW

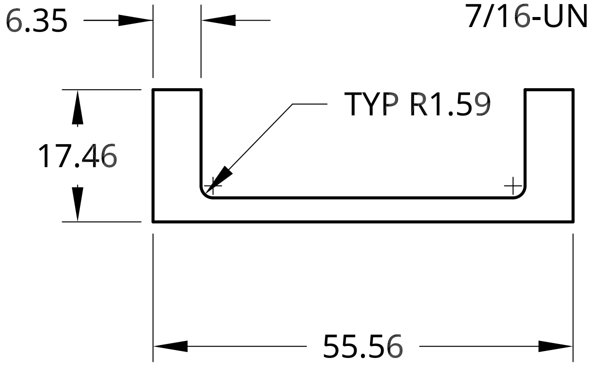

1-FRAME
ELEVATION VIEW

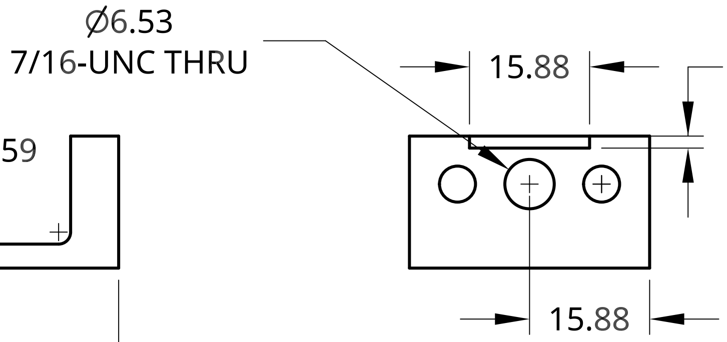

1-FRAME
SIDE VIEW

DWG NO.	SIZE	SCALE	SHEET	REV.
SI-2-2	A	1:1	2 of 2	A
