# Supplementary figures and images for: Open-source micro-tensile testers via additive manufacturing for the mechanical characterization of thin films and papers
Source: PLoS One. 2018 May 29;13(5):e0197999. doi: 10.1371/journal.pone.0197999 (PMC5973562; doi:10.1371/journal.pone.0197999)

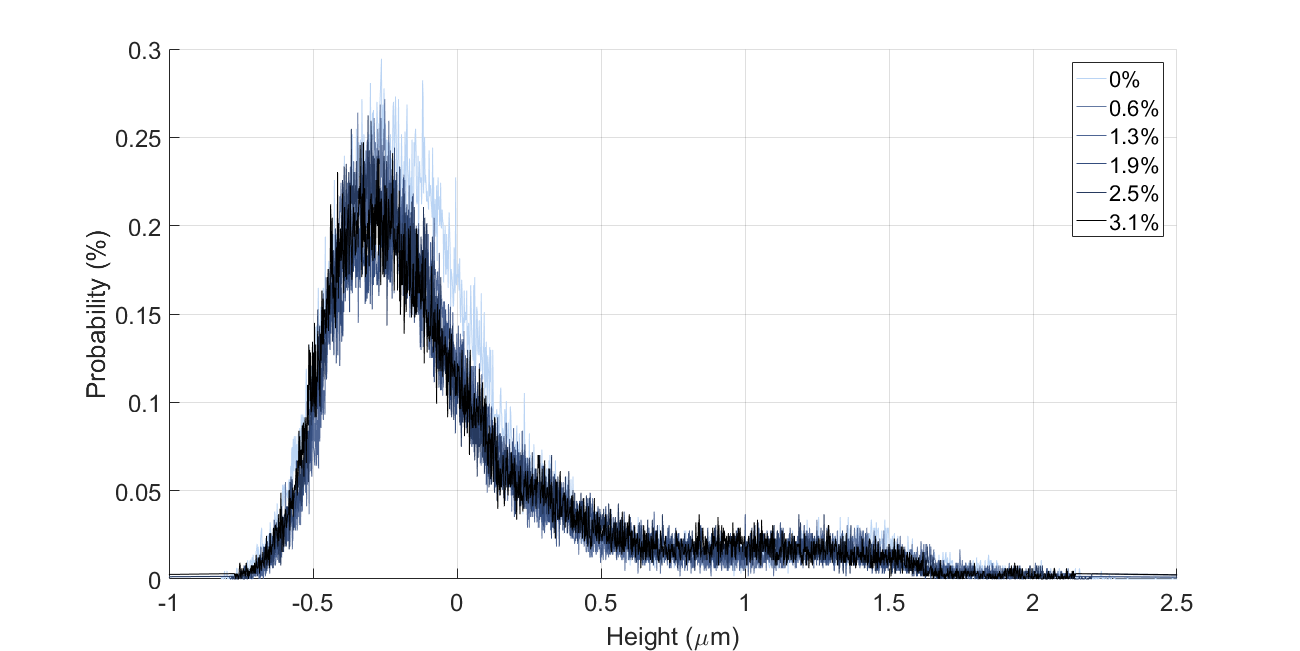

Supplement: S1 Data — (ZIP) [file pone.0197999.s002.zip › SI2_Data/Demonstration Problem 1/Exp1 Results/Area1 Height/Area1_height.png]

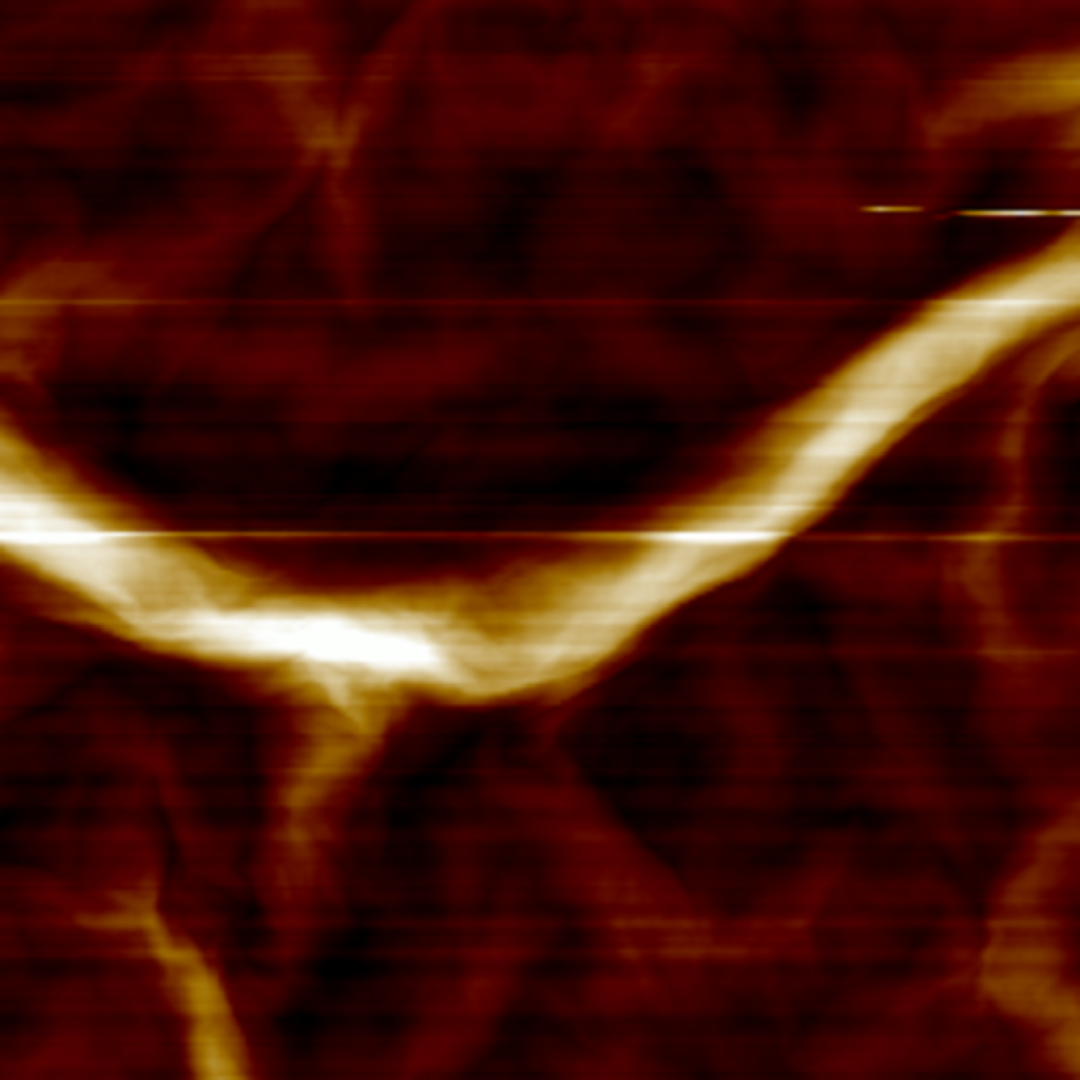

Supplement: S1 Data — (ZIP) [file pone.0197999.s002.zip › SI2_Data/Demonstration Problem 1/Exp1 Results/Area1 Height/Sample1_Area1_0turns_50um_1.001.png]

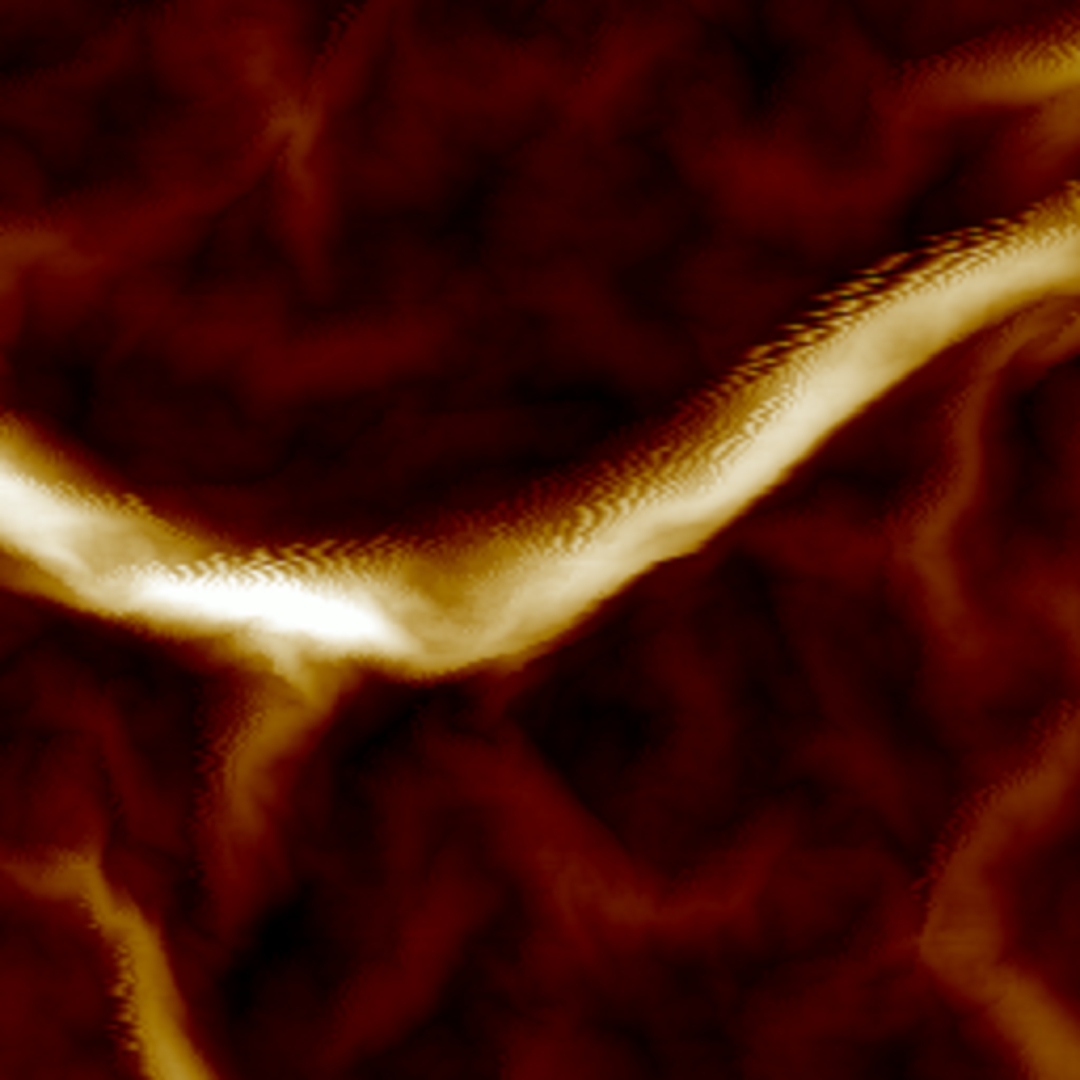

Supplement: S1 Data — (ZIP) [file pone.0197999.s002.zip › SI2_Data/Demonstration Problem 1/Exp1 Results/Area1 Height/Sample1_Area1_0_5turns_50um_1.003.png]

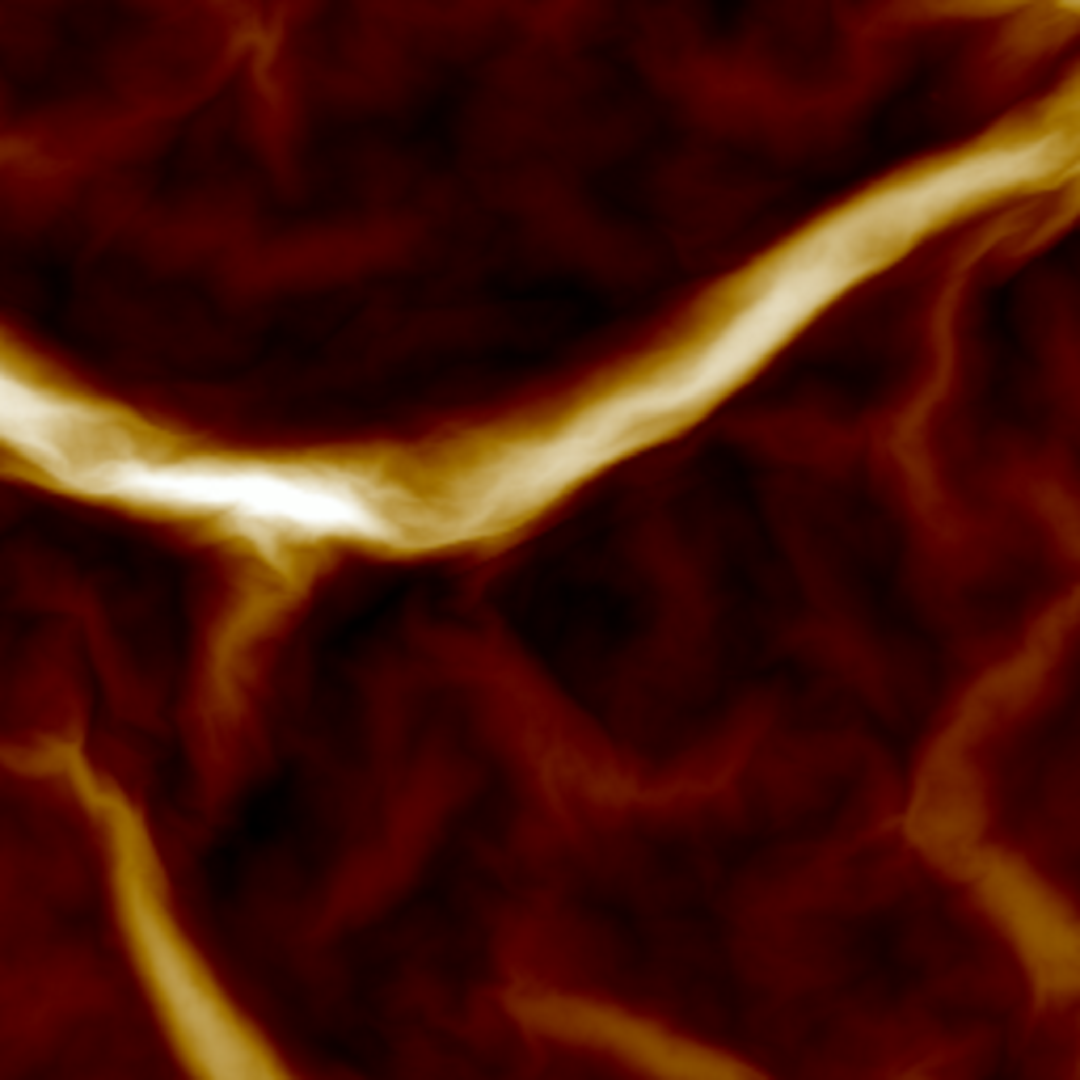

Supplement: S1 Data — (ZIP) [file pone.0197999.s002.zip › SI2_Data/Demonstration Problem 1/Exp1 Results/Area1 Height/Sample1_Area1_1_0turns_50um_1.005.png]

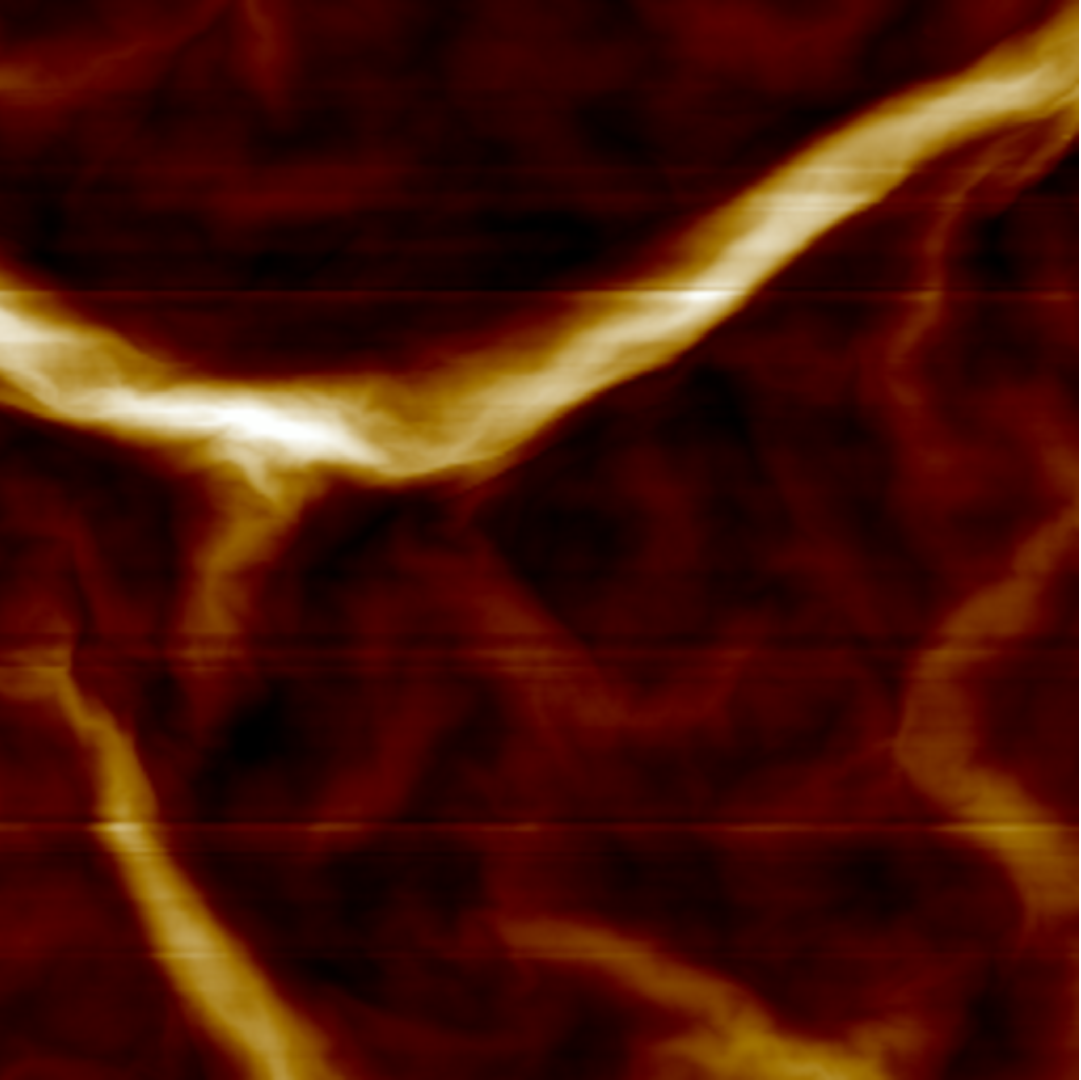

Supplement: S1 Data — (ZIP) [file pone.0197999.s002.zip › SI2_Data/Demonstration Problem 1/Exp1 Results/Area1 Height/Sample1_Area1_1_5turns_50um_1.007.png]

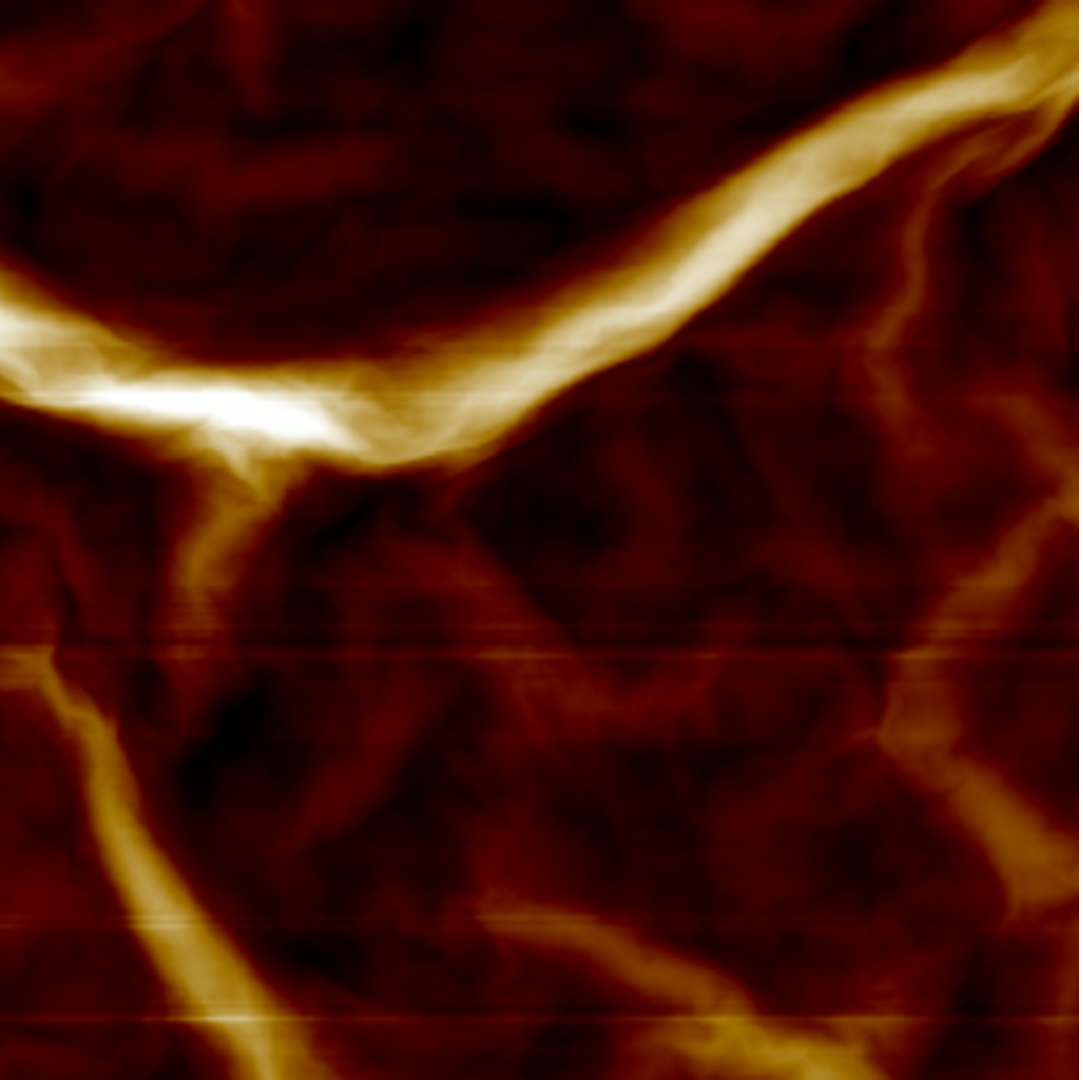

Supplement: S1 Data — (ZIP) [file pone.0197999.s002.zip › SI2_Data/Demonstration Problem 1/Exp1 Results/Area1 Height/Sample1_Area1_2_0turns_50um_1.009.png]

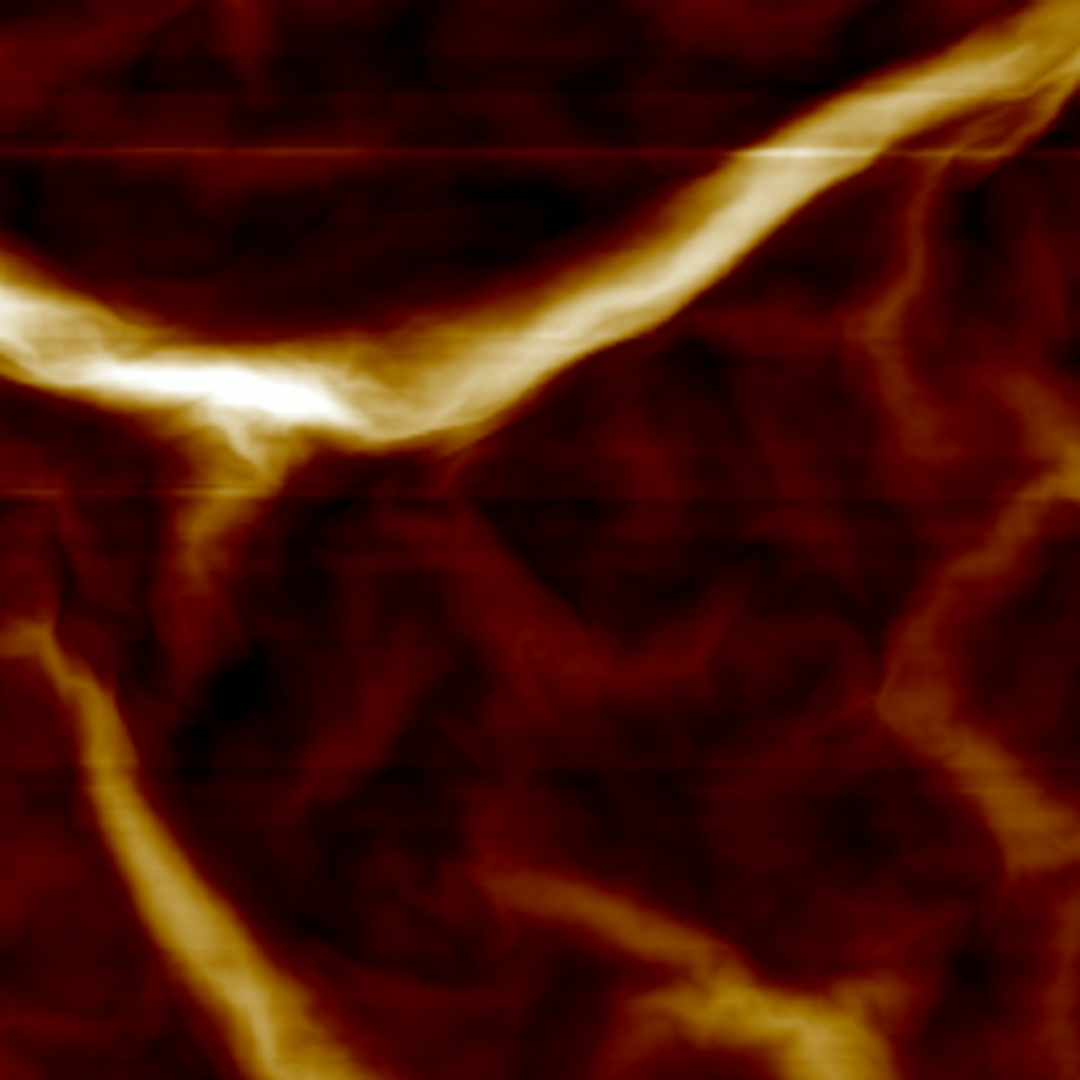

Supplement: S1 Data — (ZIP) [file pone.0197999.s002.zip › SI2_Data/Demonstration Problem 1/Exp1 Results/Area1 Height/Sample1_Area1_2_5turns_50um_1.011.png]

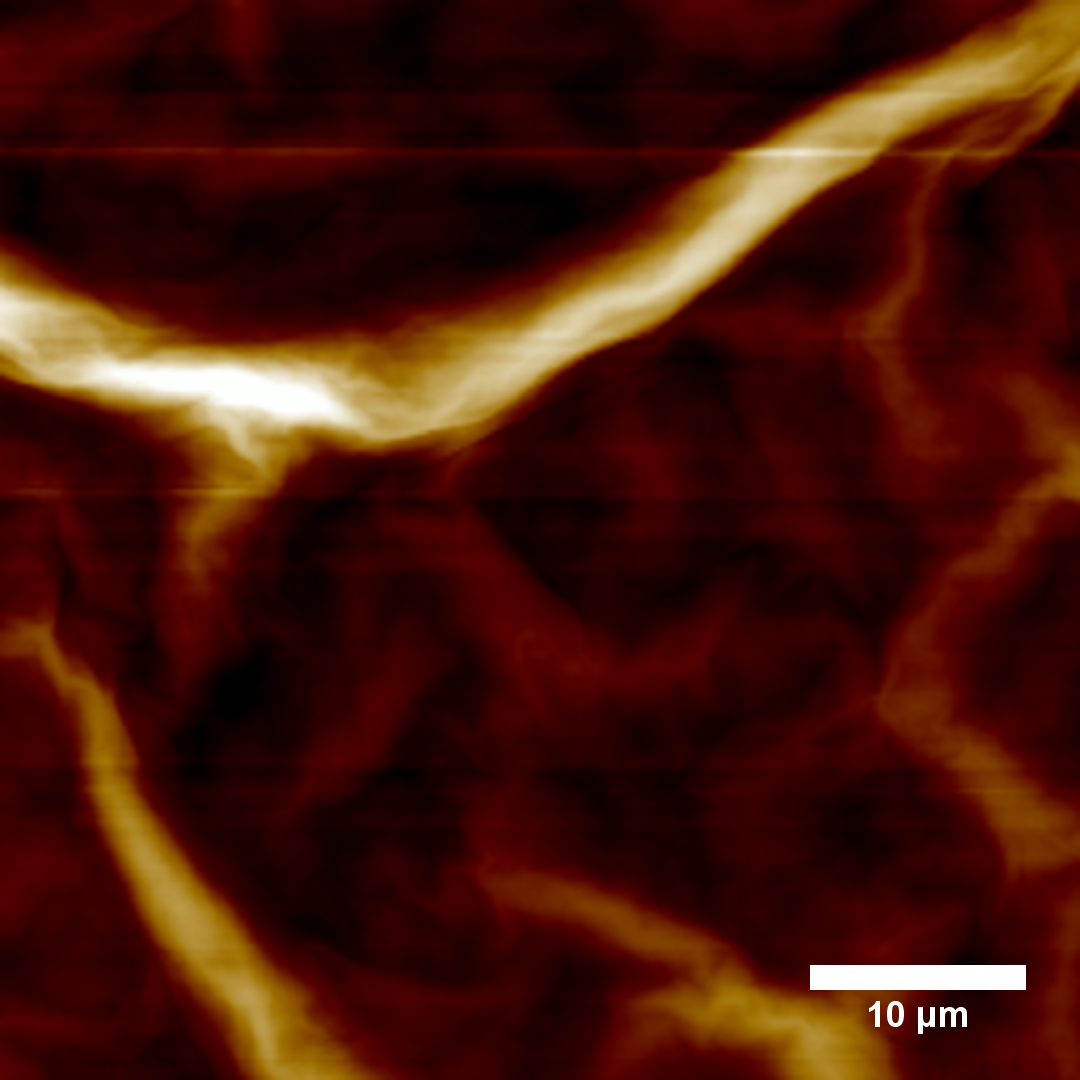

Supplement: S1 Data — (ZIP) [file pone.0197999.s002.zip › SI2_Data/Demonstration Problem 1/Exp1 Results/Area1 Height/Sample1_Area1_2_5turns_50um_sb.png]

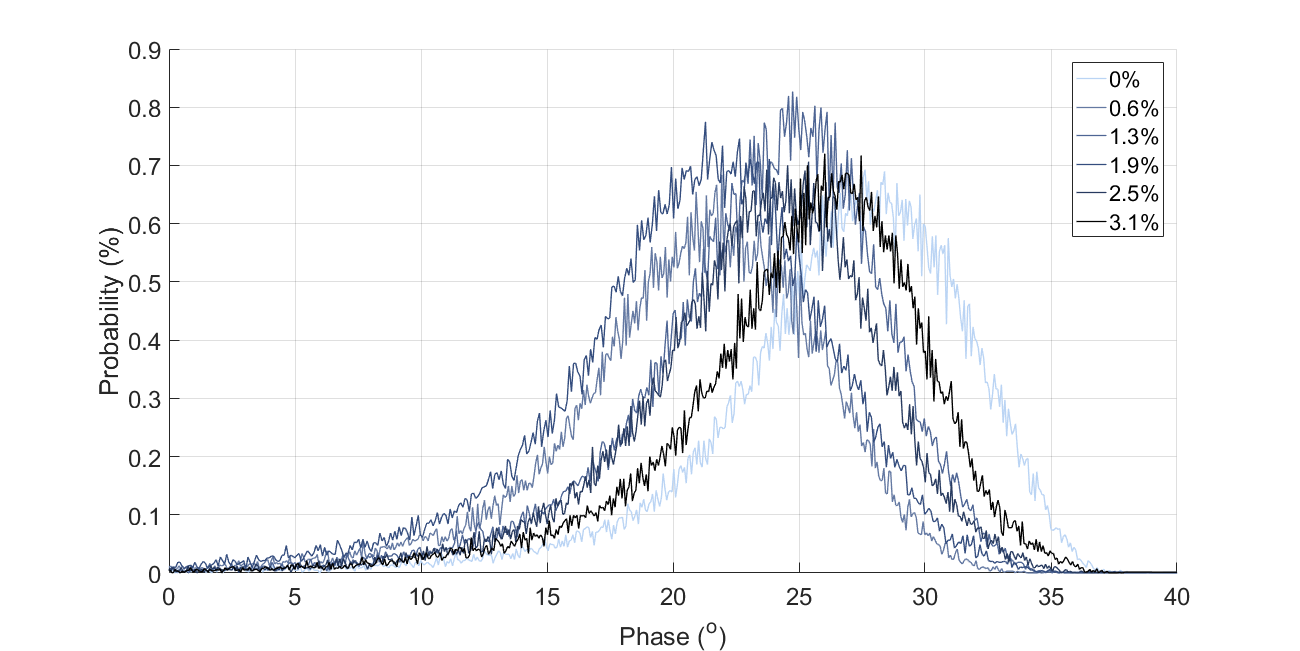

Supplement: S1 Data — (ZIP) [file pone.0197999.s002.zip › SI2_Data/Demonstration Problem 1/Exp1 Results/Area2 Phase/Phase_Histo_Area2.png]

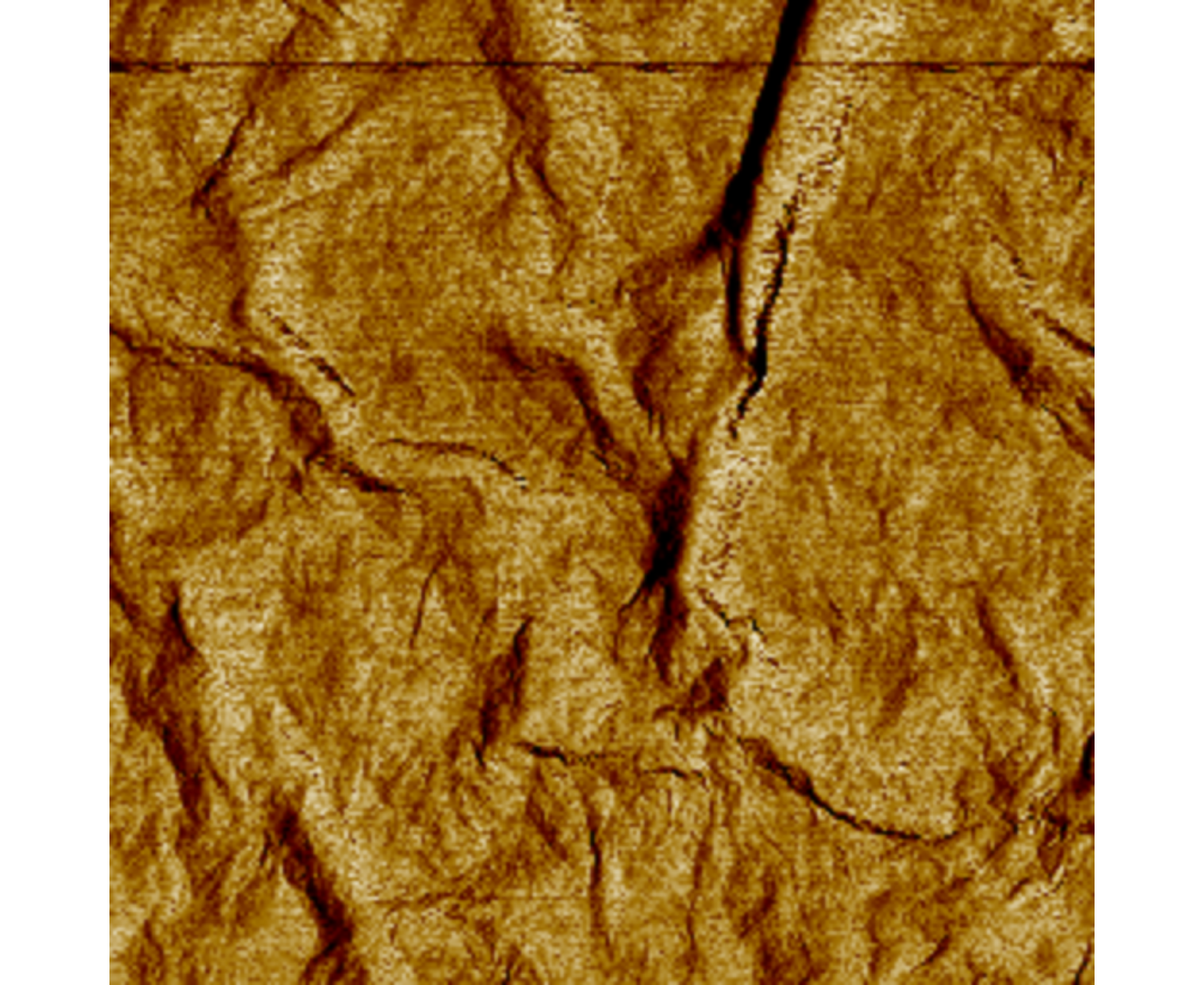

Supplement: S1 Data — (ZIP) [file pone.0197999.s002.zip › SI2_Data/Demonstration Problem 1/Exp1 Results/Area2 Phase/Sample1_Area2_0-5turns_50um_3.002.png]

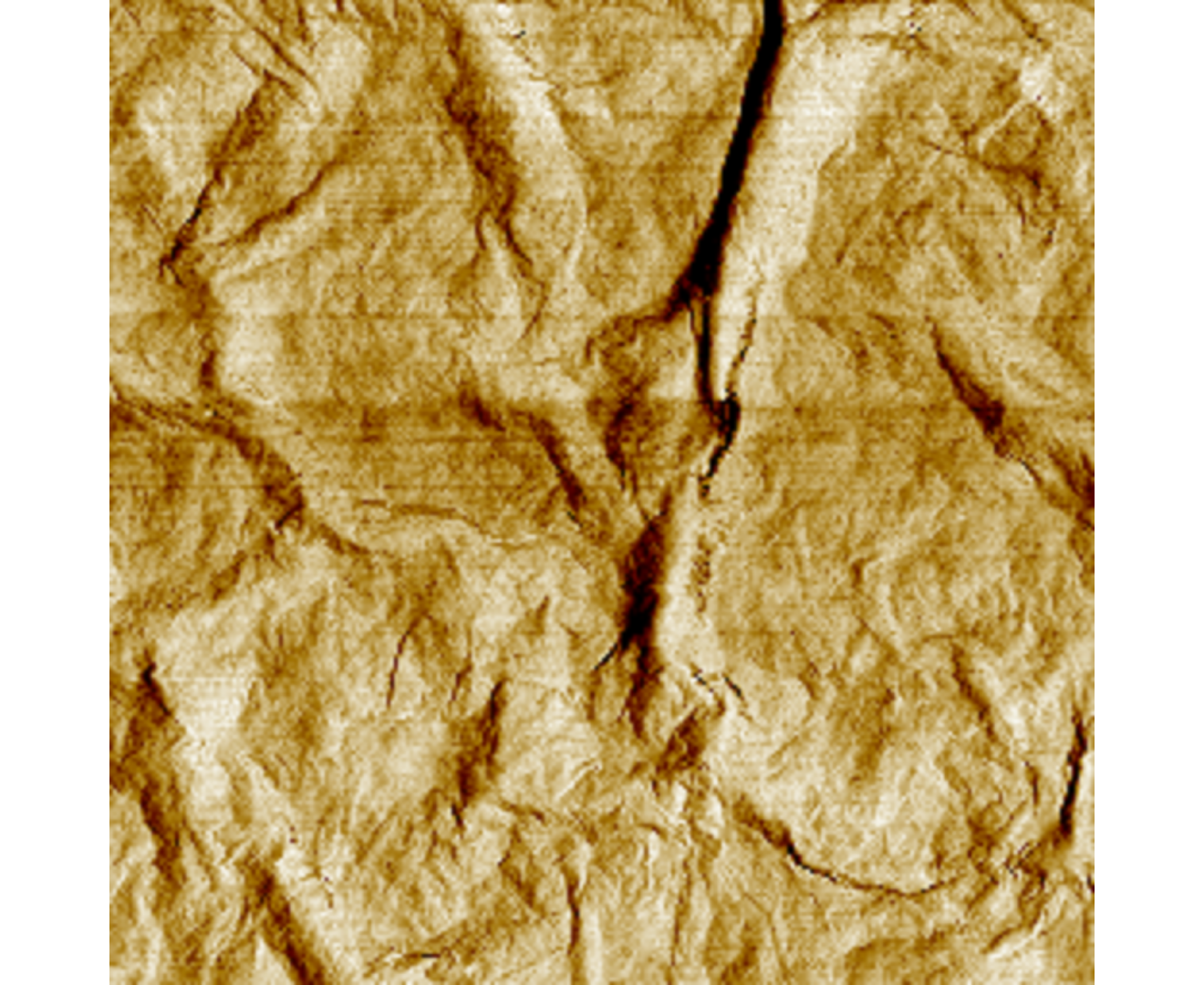

Supplement: S1 Data — (ZIP) [file pone.0197999.s002.zip › SI2_Data/Demonstration Problem 1/Exp1 Results/Area2 Phase/Sample1_Area2_0turns_50um_3.002.png]

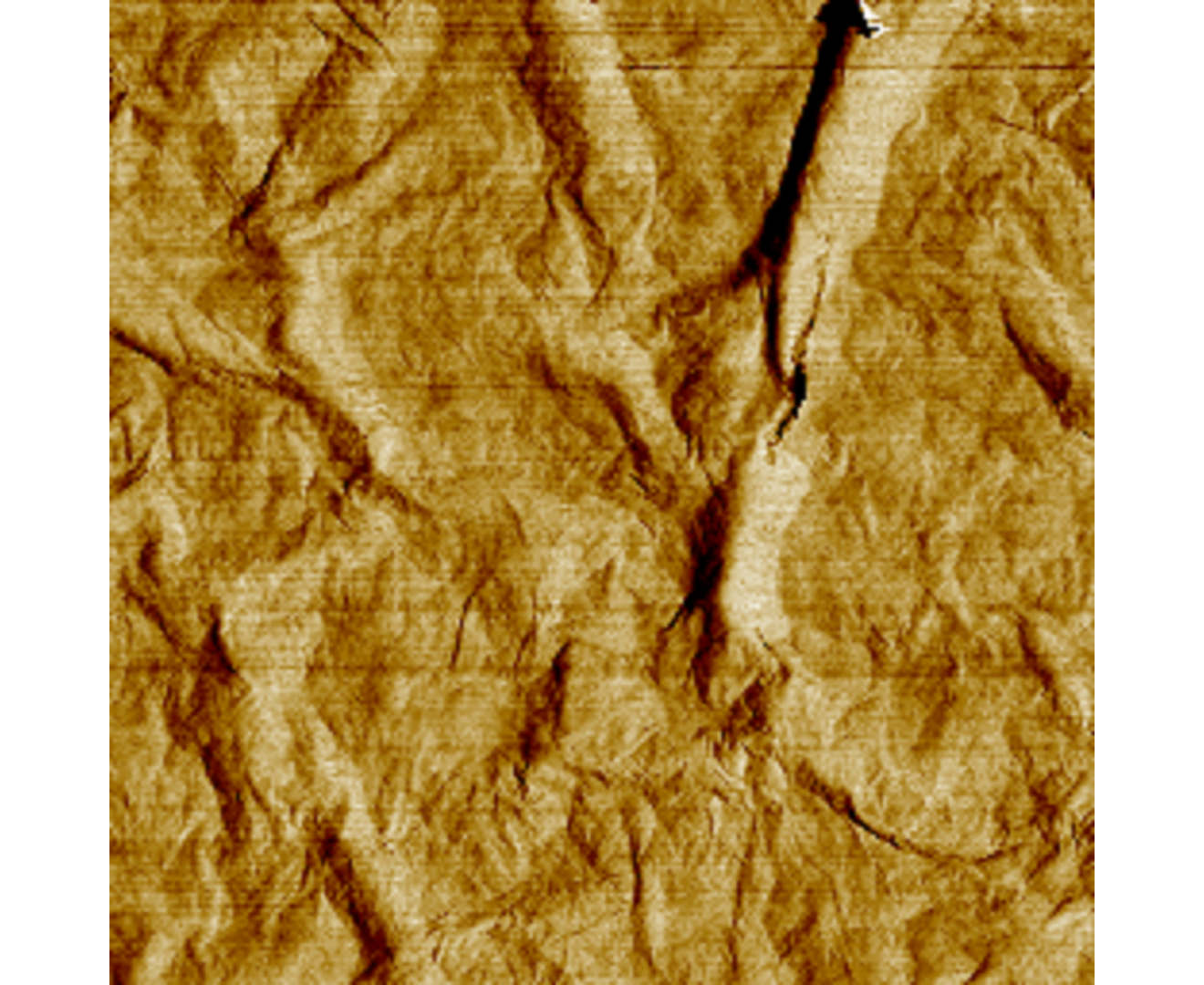

Supplement: S1 Data — (ZIP) [file pone.0197999.s002.zip › SI2_Data/Demonstration Problem 1/Exp1 Results/Area2 Phase/Sample1_Area2_1_0turns_50um_3.006.png]

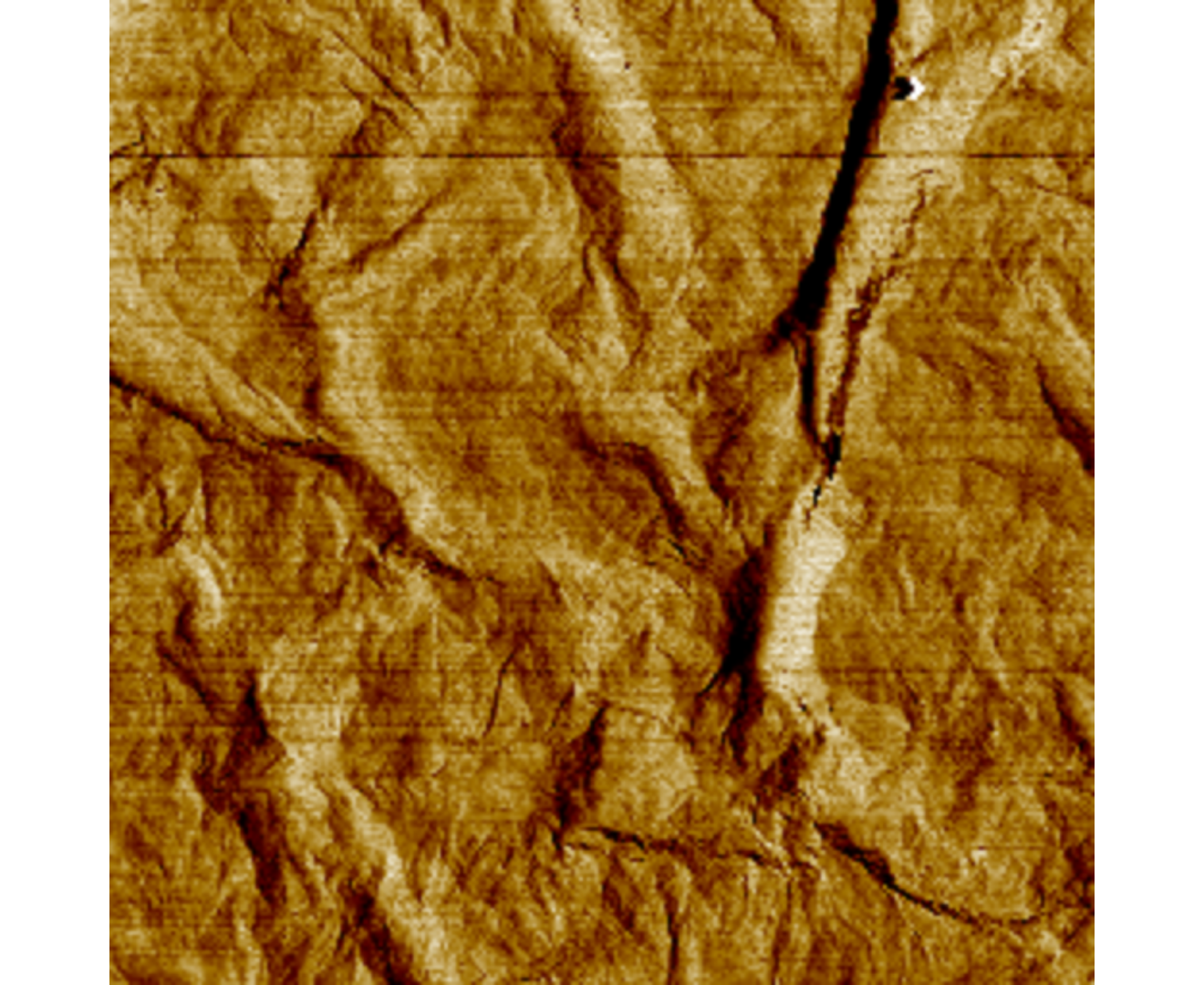

Supplement: S1 Data — (ZIP) [file pone.0197999.s002.zip › SI2_Data/Demonstration Problem 1/Exp1 Results/Area2 Phase/Sample1_Area2_1_5turns_50um_3.008.png]

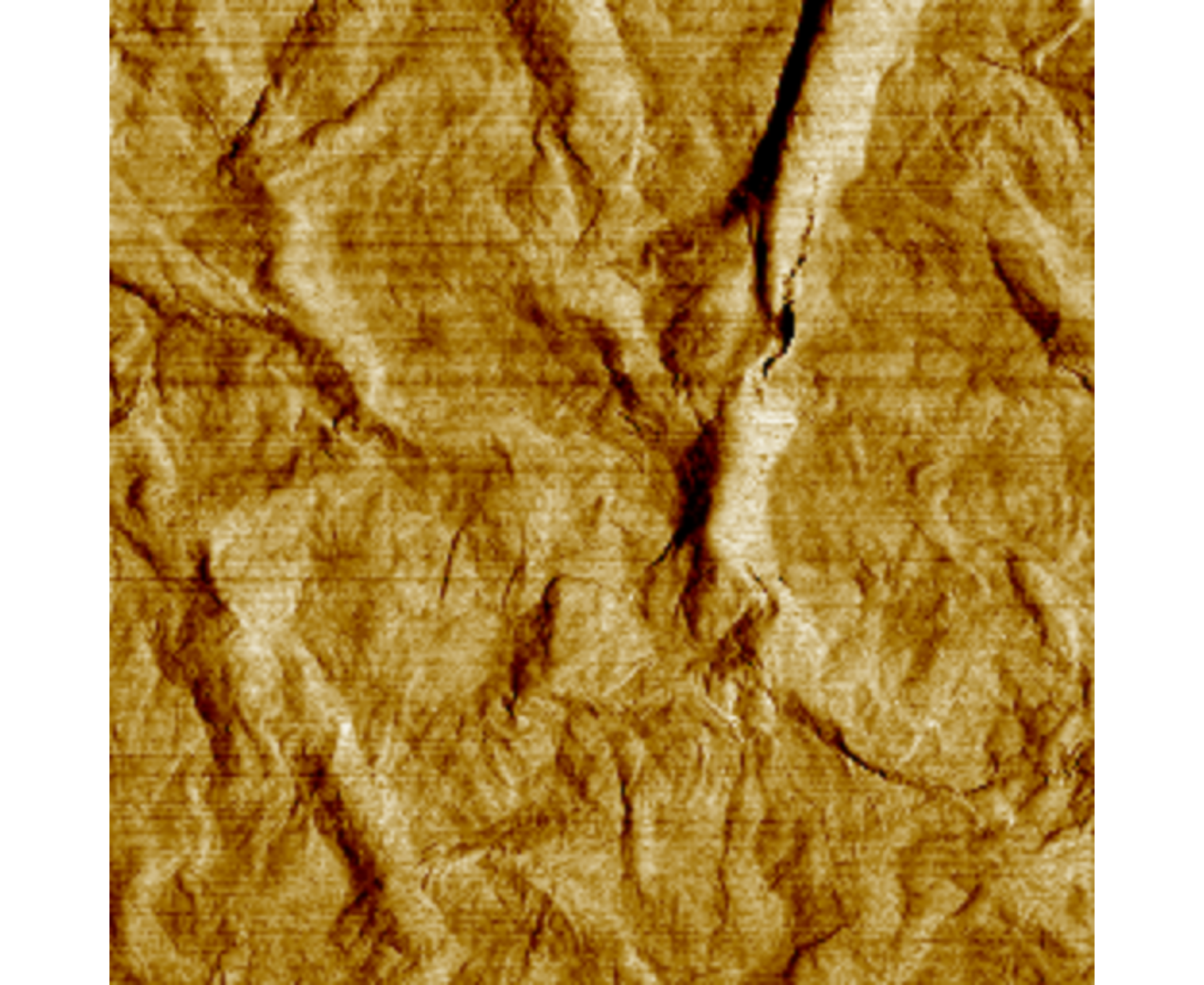

Supplement: S1 Data — (ZIP) [file pone.0197999.s002.zip › SI2_Data/Demonstration Problem 1/Exp1 Results/Area2 Phase/Sample1_Area2_2_0turns_50um_3.010.png]

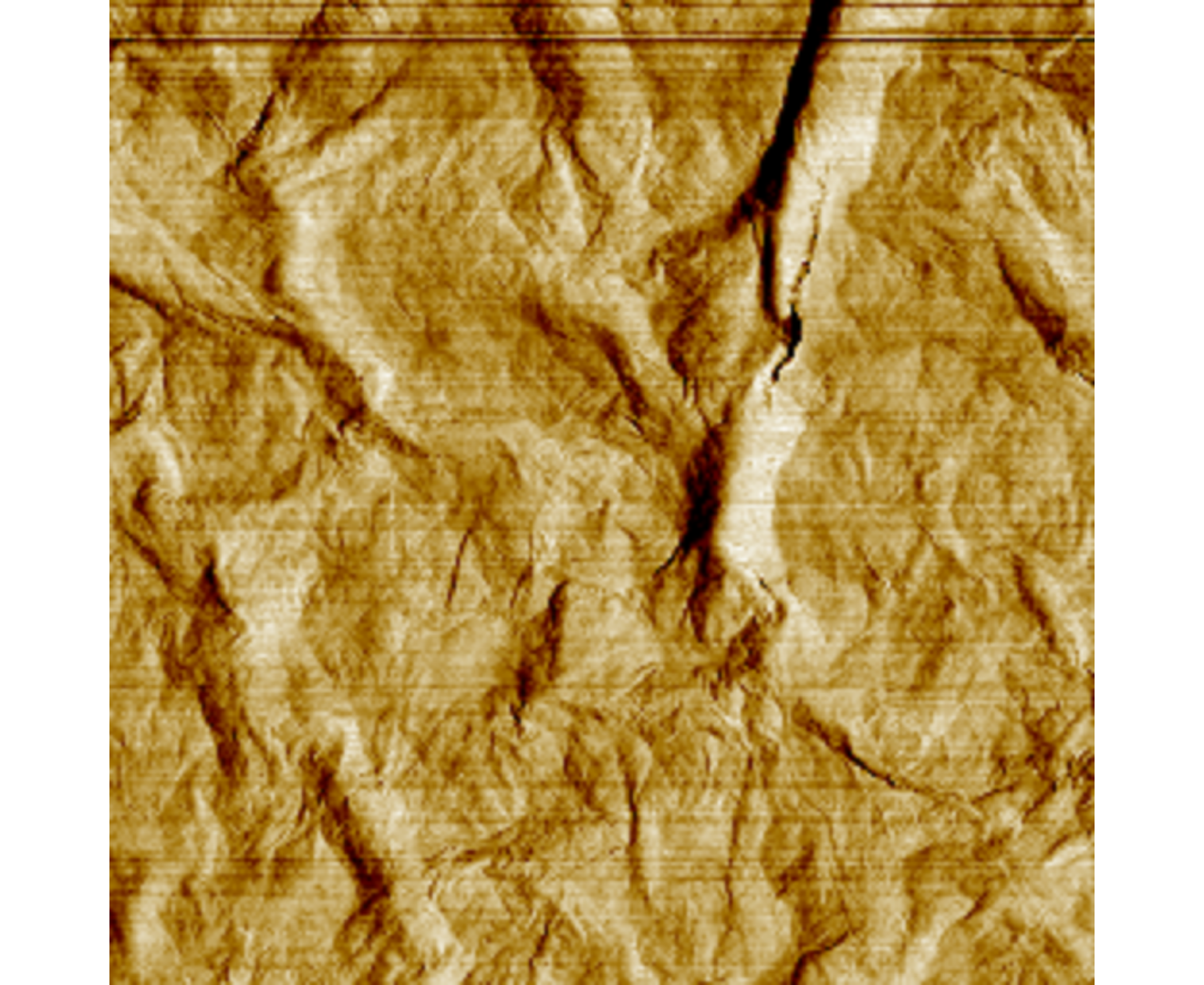

Supplement: S1 Data — (ZIP) [file pone.0197999.s002.zip › SI2_Data/Demonstration Problem 1/Exp1 Results/Area2 Phase/Sample1_Area2_2_5turns_50um_3.012.png]

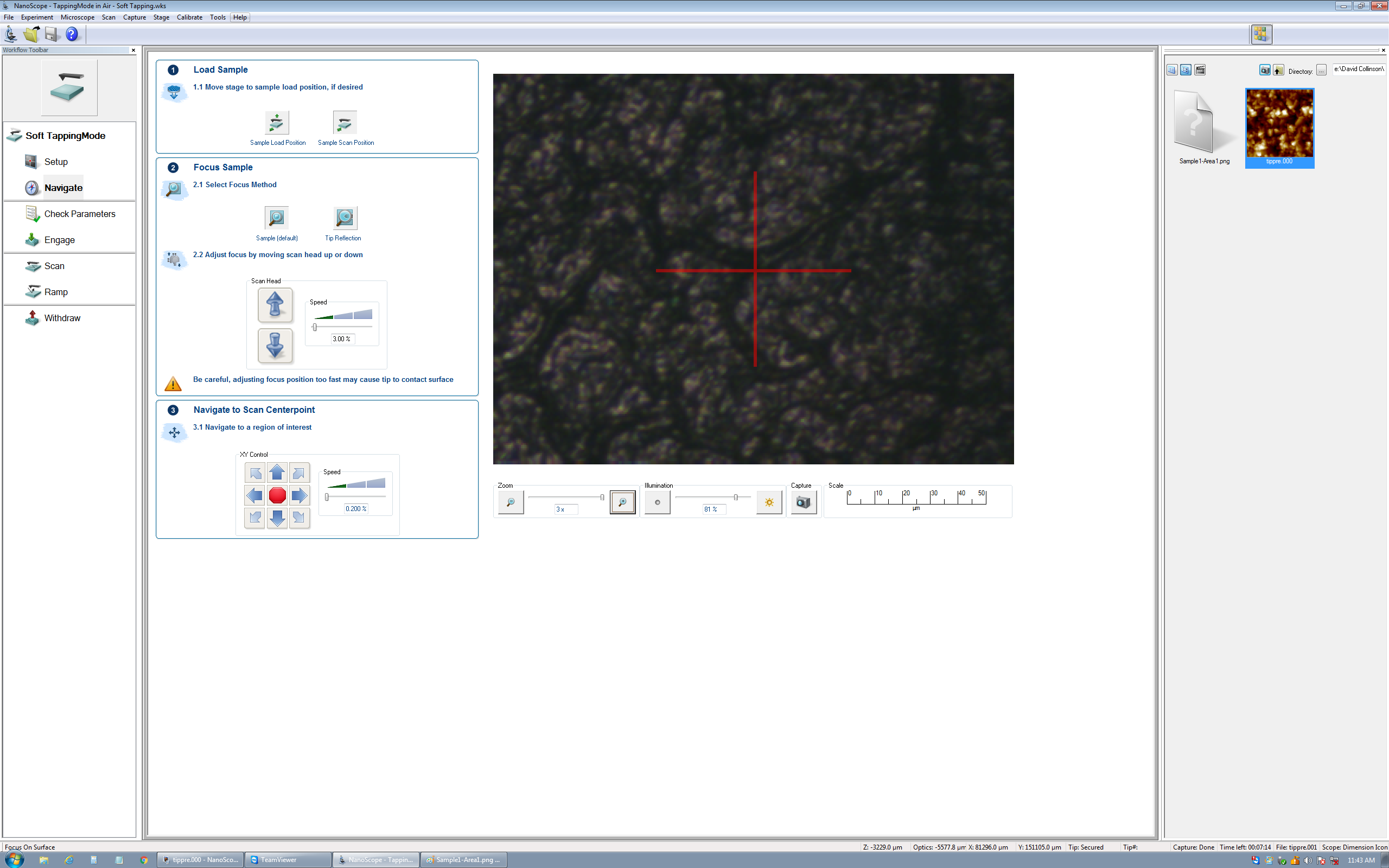

Supplement: S1 Data — (ZIP) [file pone.0197999.s002.zip › SI2_Data/Demonstration Problem 1/Exp1/Sample1-Area1-zoom.png]

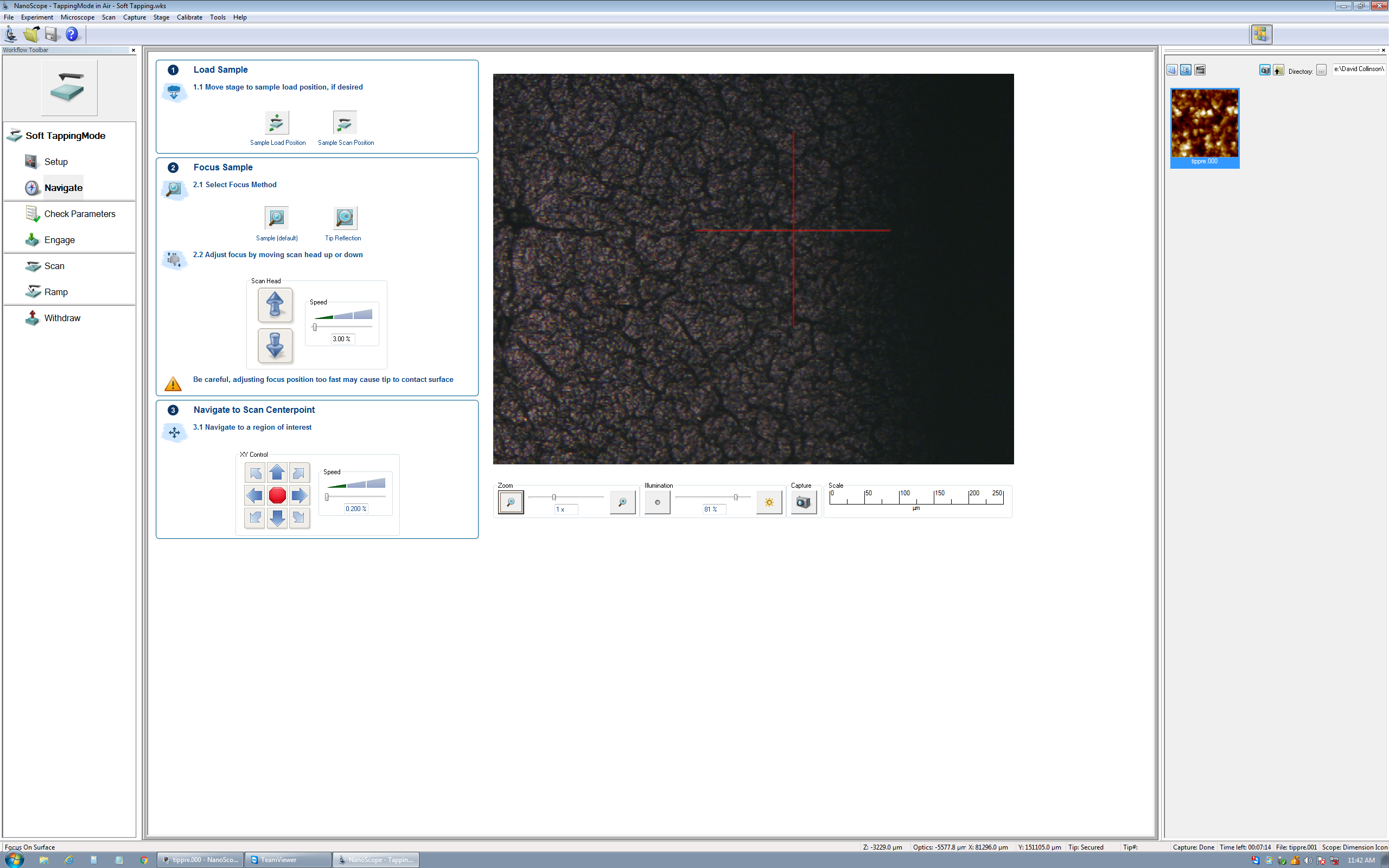

Supplement: S1 Data — (ZIP) [file pone.0197999.s002.zip › SI2_Data/Demonstration Problem 1/Exp1/Sample1-Area1.png]

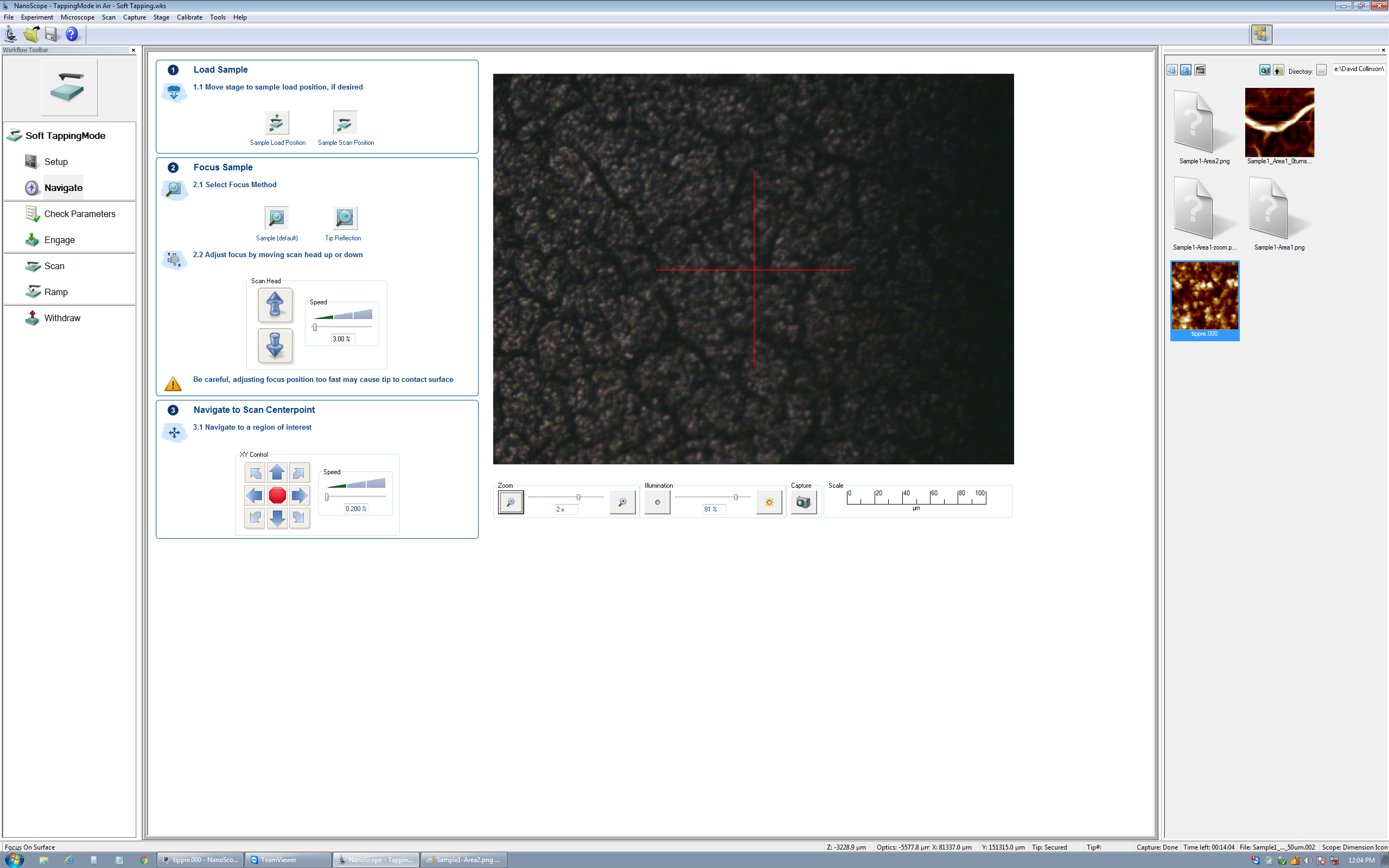

Supplement: S1 Data — (ZIP) [file pone.0197999.s002.zip › SI2_Data/Demonstration Problem 1/Exp1/Sample1-Area2-zoom.png]

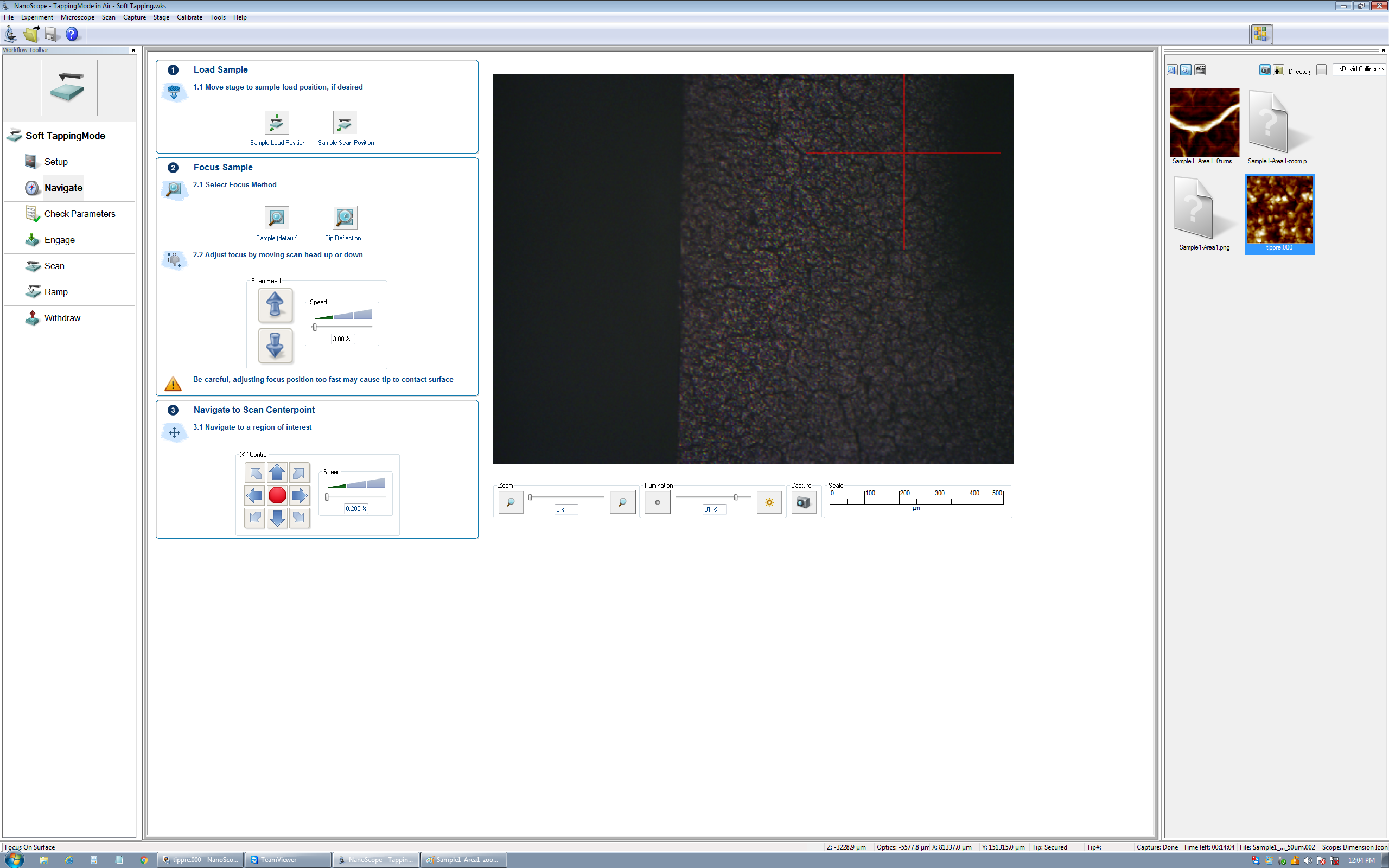

Supplement: S1 Data — (ZIP) [file pone.0197999.s002.zip › SI2_Data/Demonstration Problem 1/Exp1/Sample1-Area2.png]

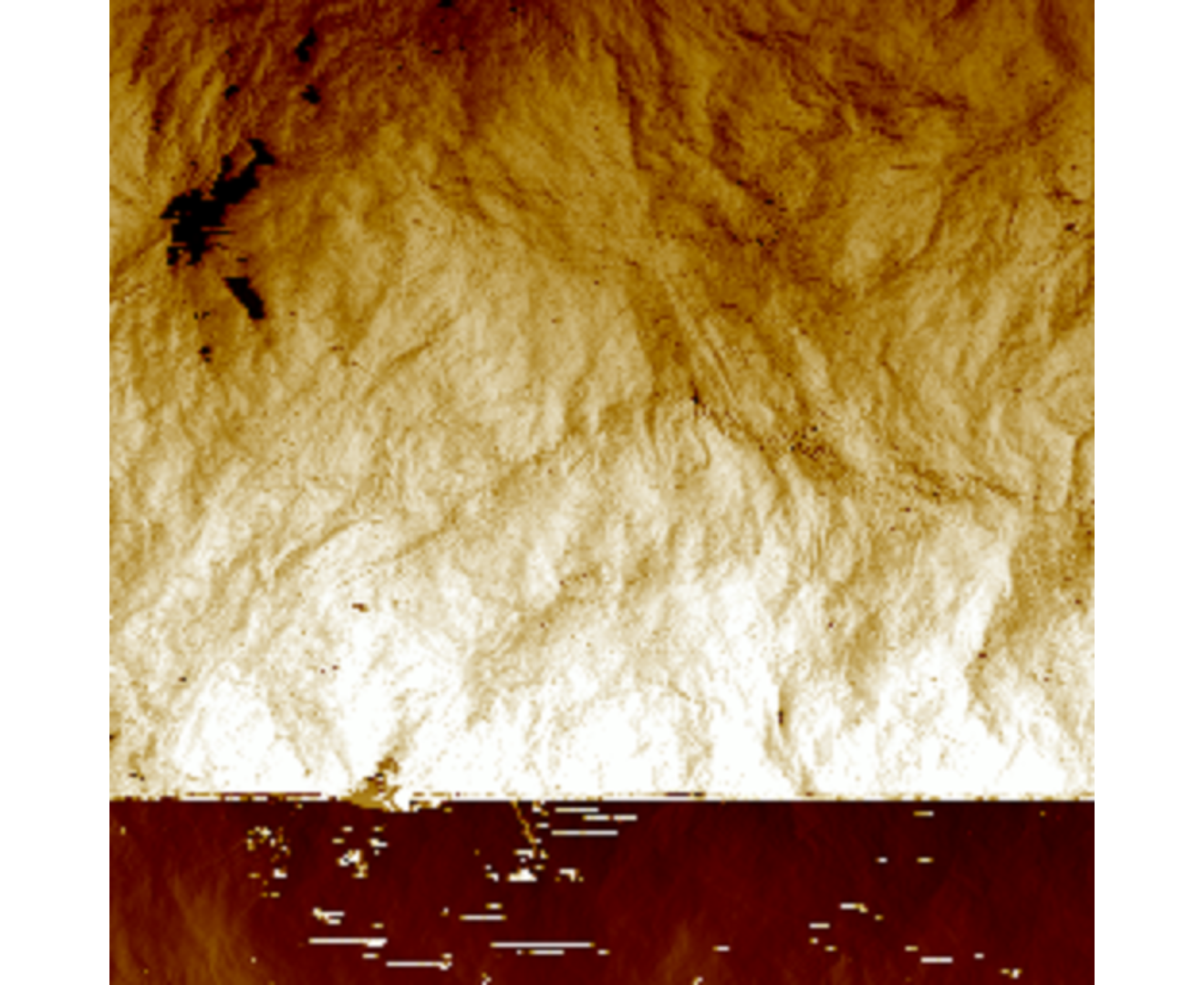

Supplement: S1 Data — (ZIP) [file pone.0197999.s002.zip › SI2_Data/Demonstration Problem 1/Exp2 Results/sample3_turn0-25.png]

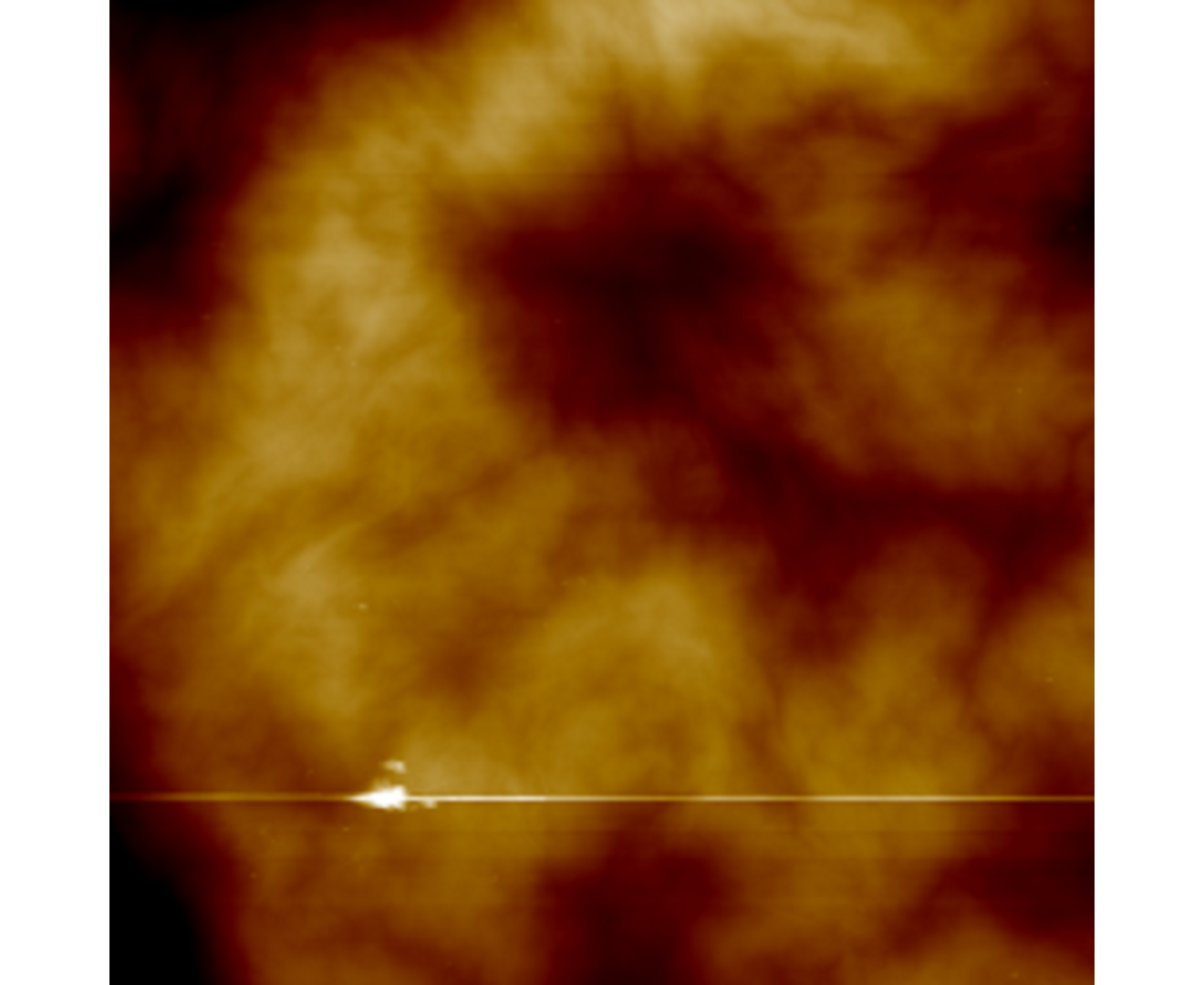

Supplement: S1 Data — (ZIP) [file pone.0197999.s002.zip › SI2_Data/Demonstration Problem 1/Exp2 Results/sample3_turn0-25_topo.png]

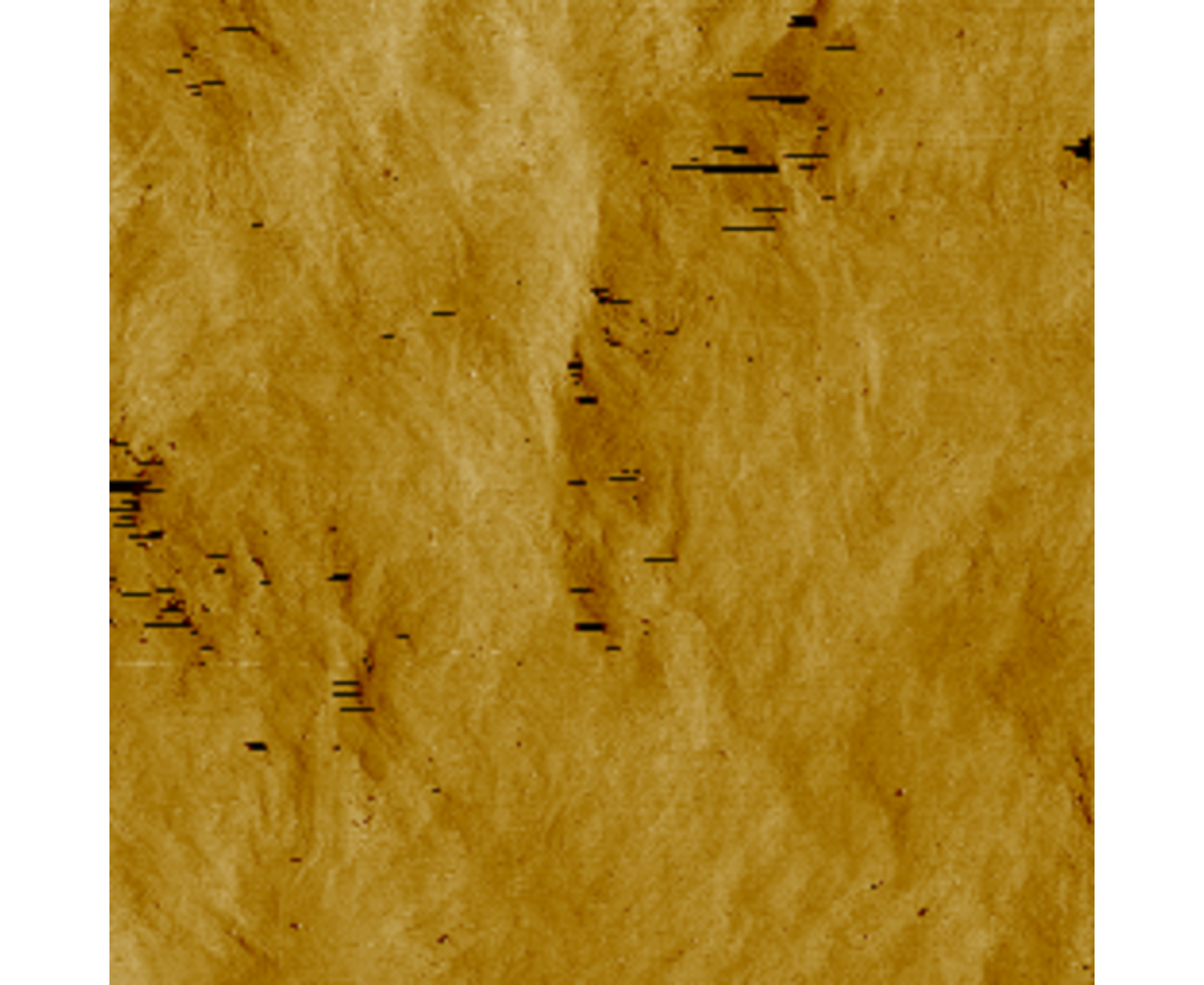

Supplement: S1 Data — (ZIP) [file pone.0197999.s002.zip › SI2_Data/Demonstration Problem 1/Exp2 Results/sample3_turn0-5.png]

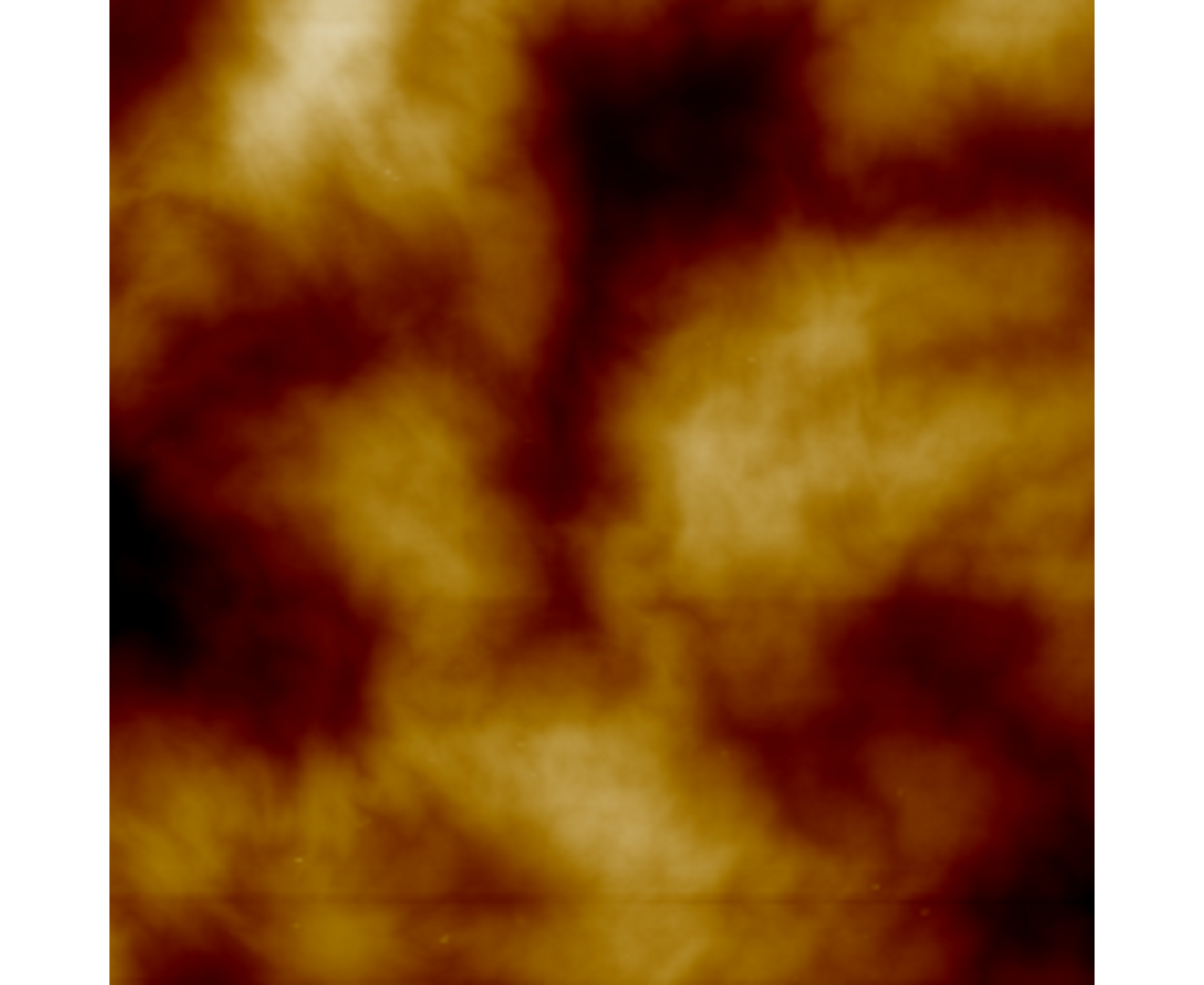

Supplement: S1 Data — (ZIP) [file pone.0197999.s002.zip › SI2_Data/Demonstration Problem 1/Exp2 Results/sample3_turn0-5_topo.png]

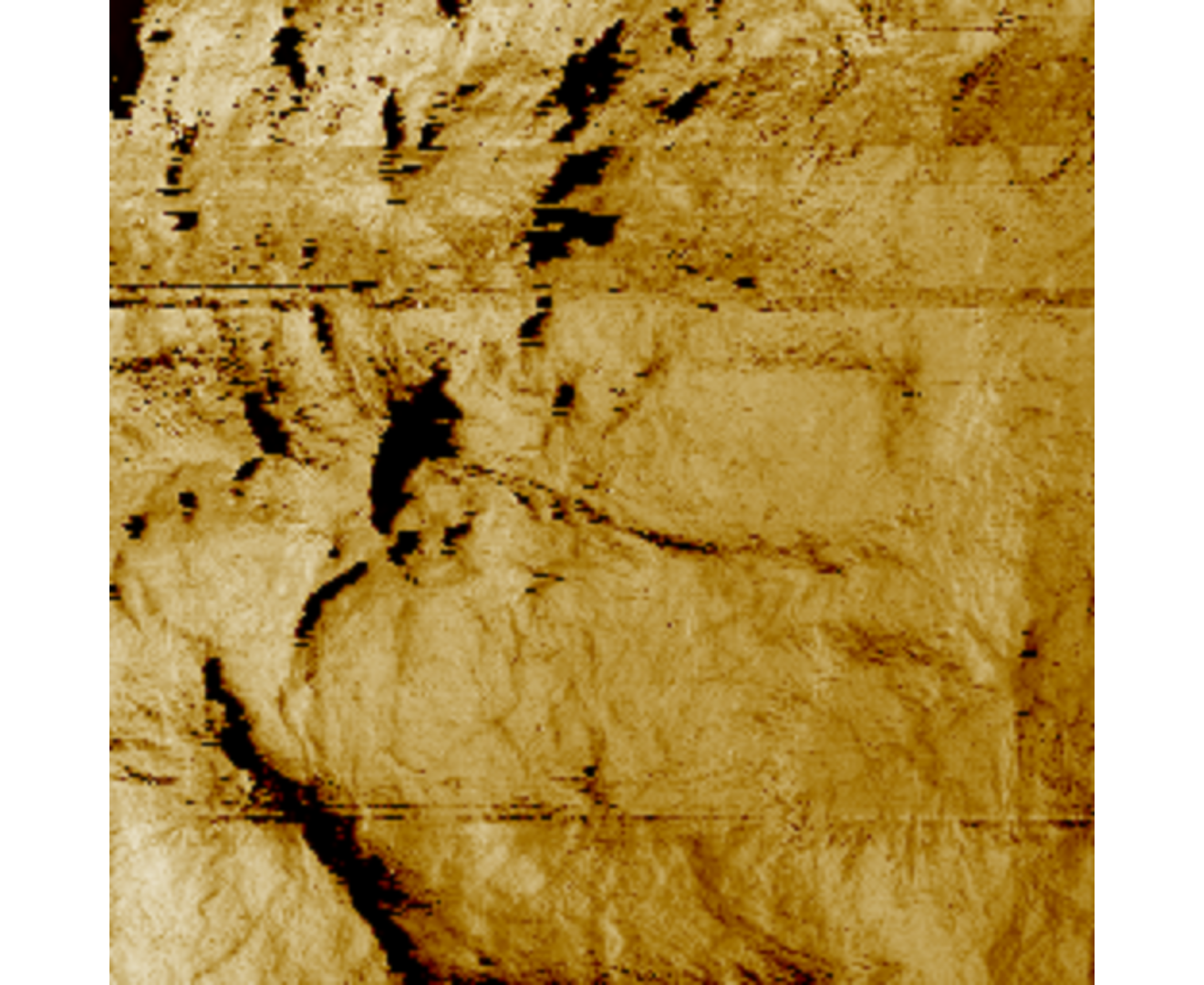

Supplement: S1 Data — (ZIP) [file pone.0197999.s002.zip › SI2_Data/Demonstration Problem 1/Exp2 Results/sample3_turn0.png]

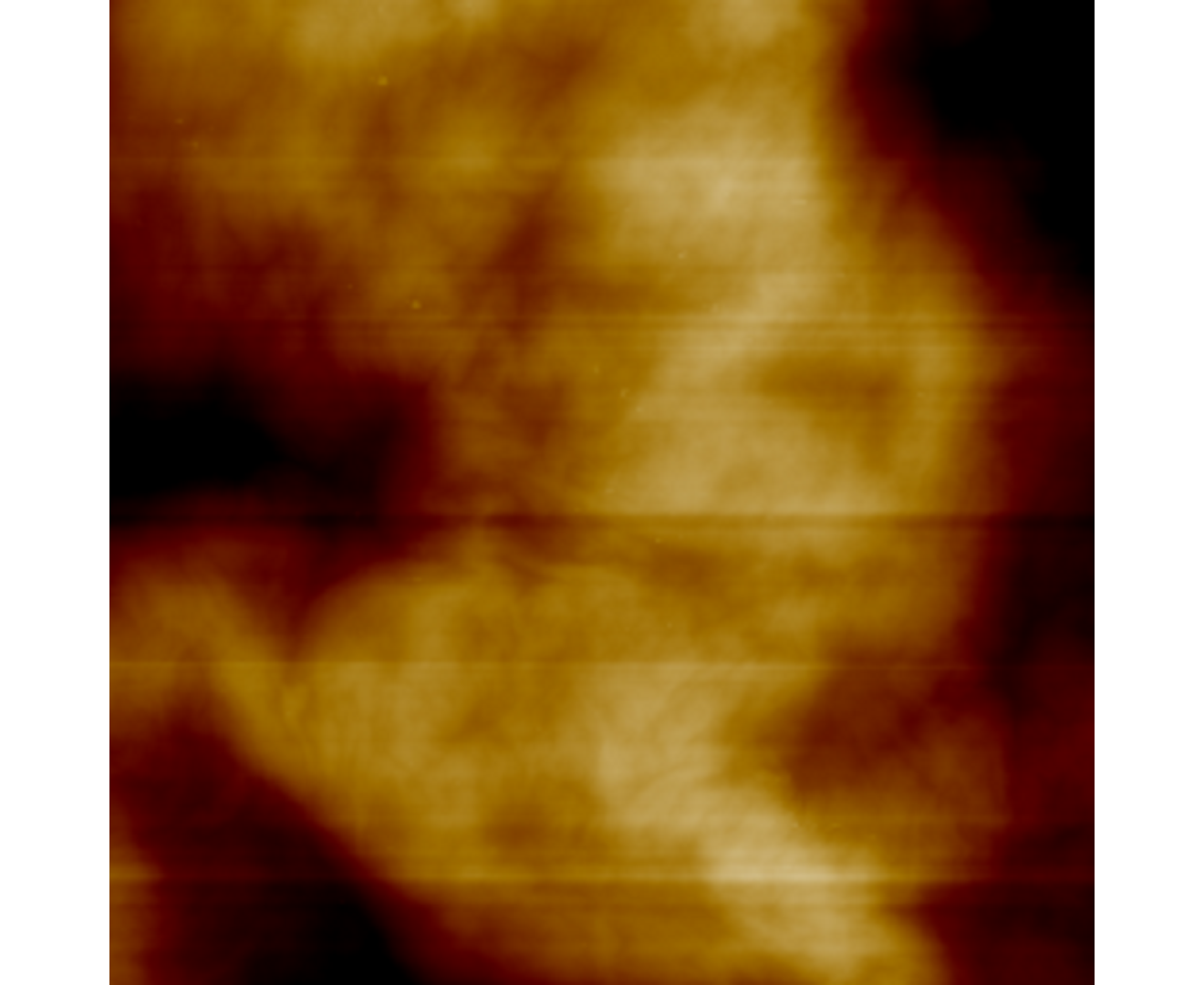

Supplement: S1 Data — (ZIP) [file pone.0197999.s002.zip › SI2_Data/Demonstration Problem 1/Exp2 Results/sample3_turn0_topo.png]

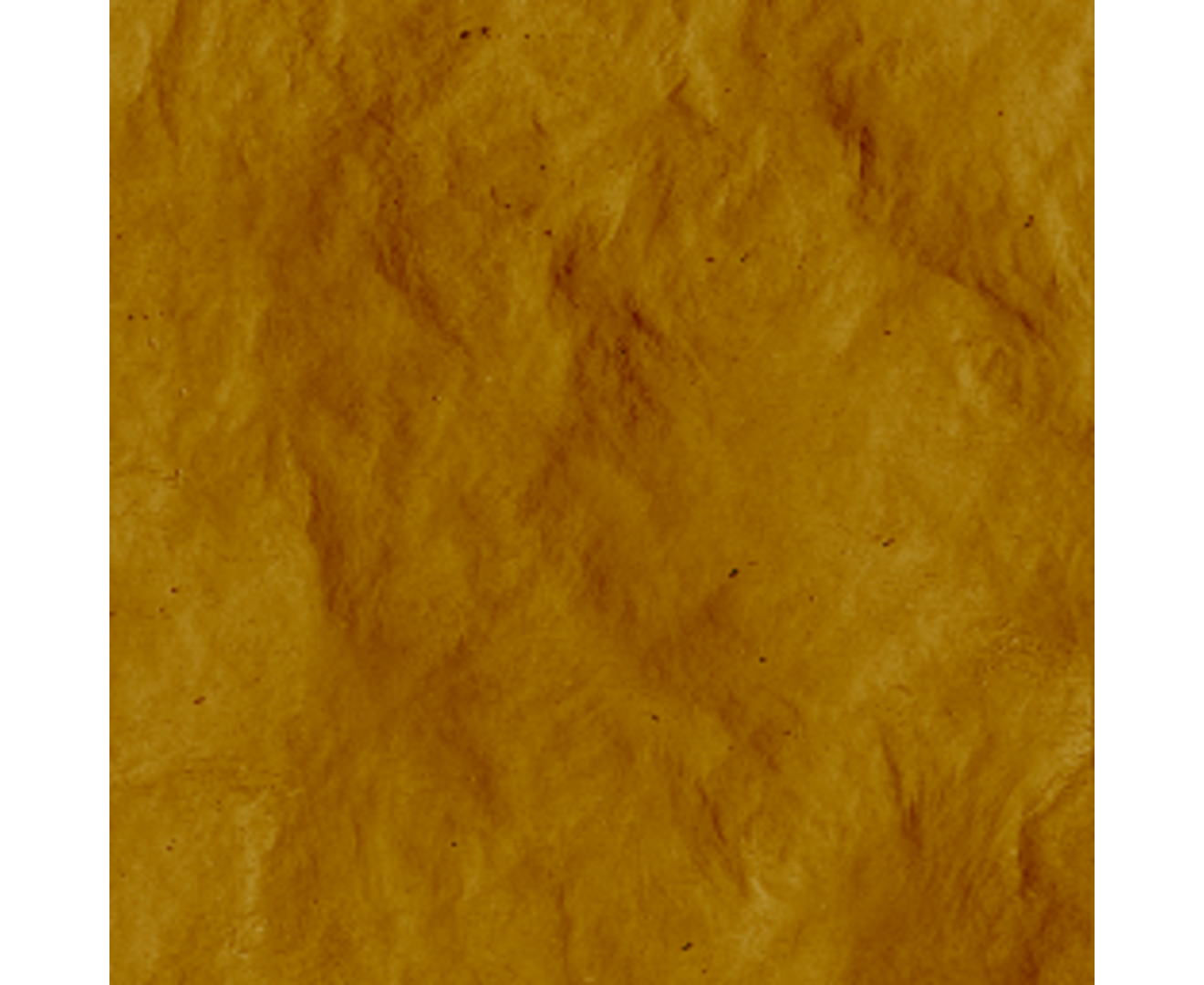

Supplement: S1 Data — (ZIP) [file pone.0197999.s002.zip › SI2_Data/Demonstration Problem 1/Exp2 Results/sample3_turn1-5.png]

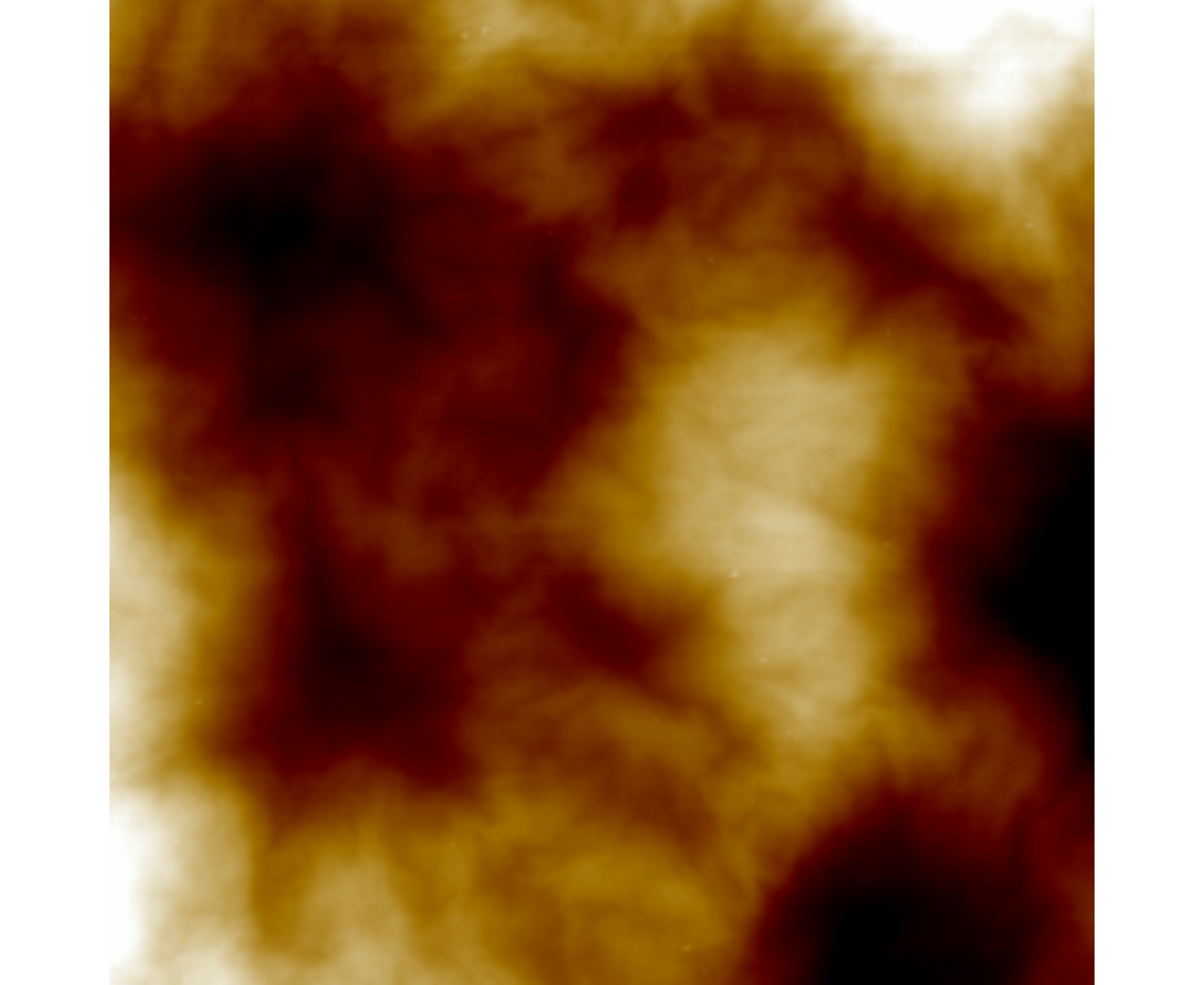

Supplement: S1 Data — (ZIP) [file pone.0197999.s002.zip › SI2_Data/Demonstration Problem 1/Exp2 Results/sample3_turn1-5_topo.png]

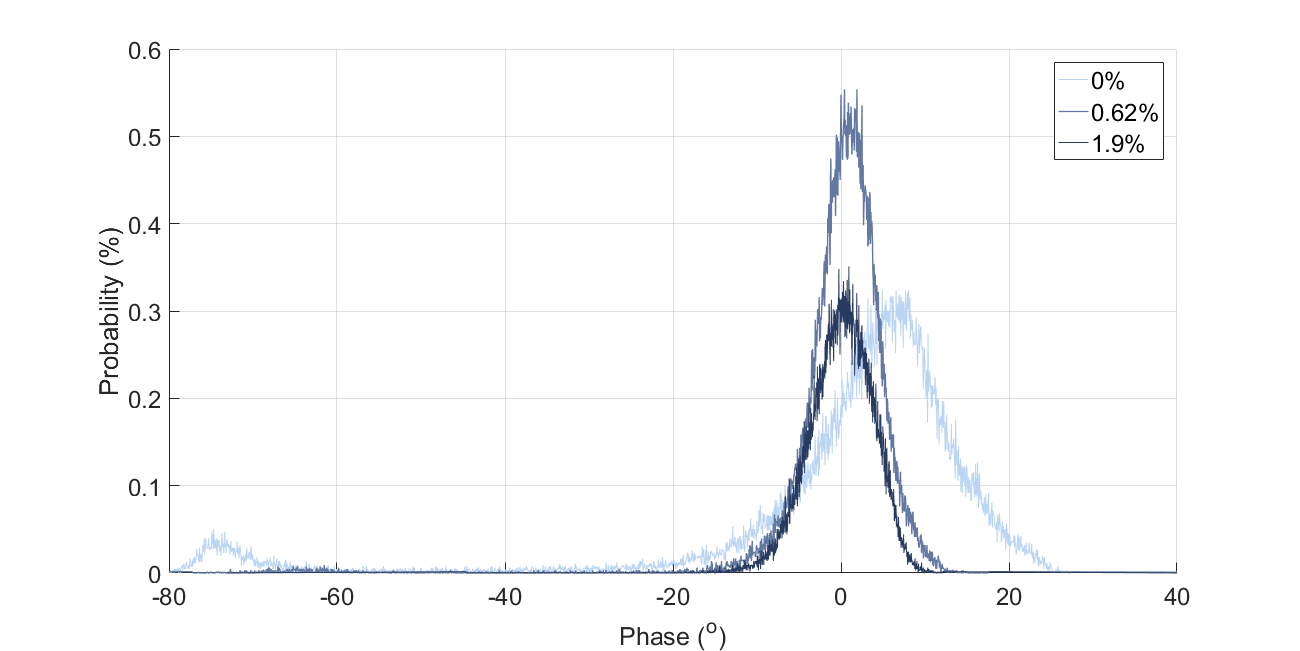

Supplement: S1 Data — (ZIP) [file pone.0197999.s002.zip › SI2_Data/Demonstration Problem 1/Exp2 Results/Summary_Histo.png]

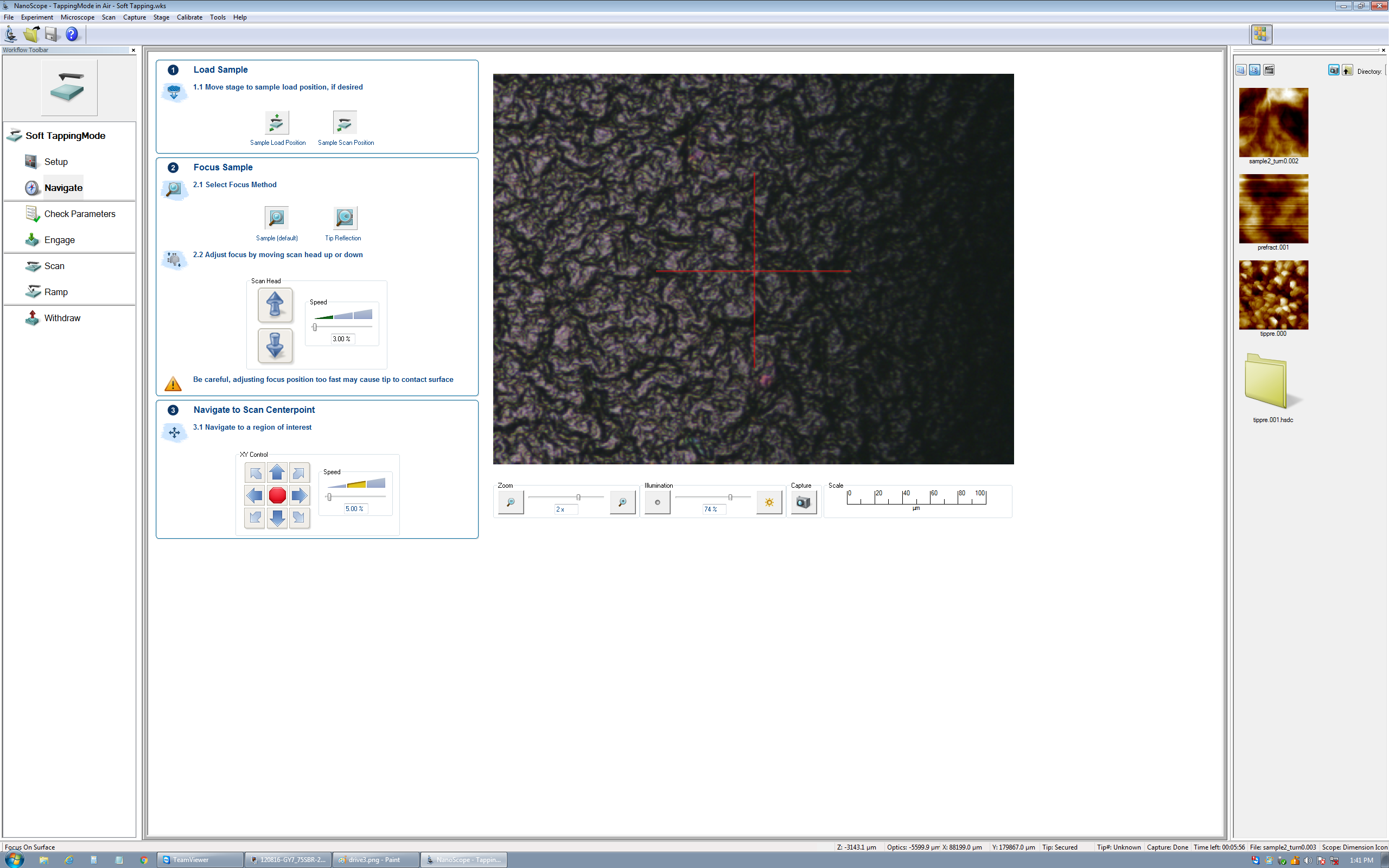

Supplement: S1 Data — (ZIP) [file pone.0197999.s002.zip › SI2_Data/Demonstration Problem 1/Exp2/sample3.png]

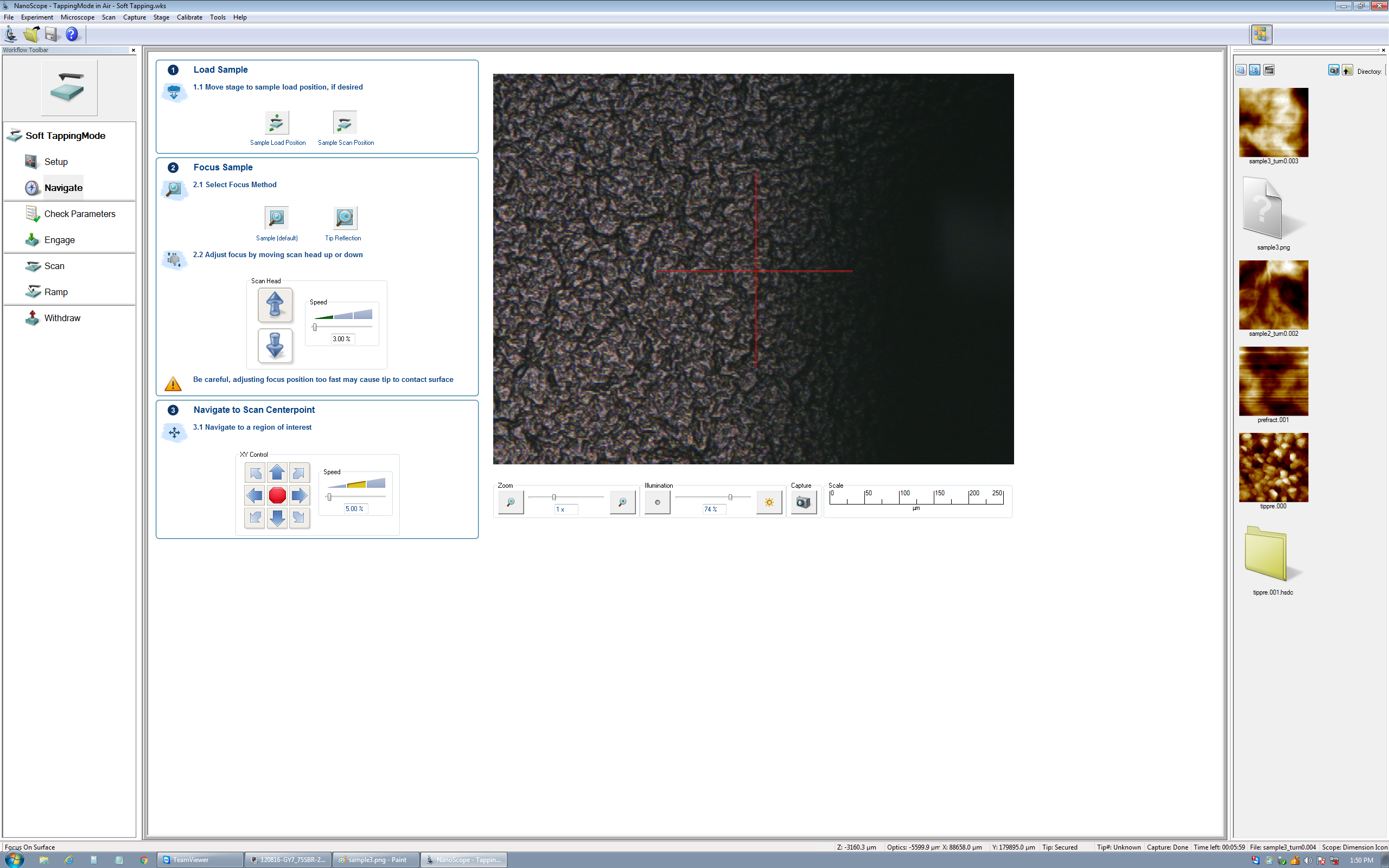

Supplement: S1 Data — (ZIP) [file pone.0197999.s002.zip › SI2_Data/Demonstration Problem 1/Exp2/sample3_0-25turn.png]

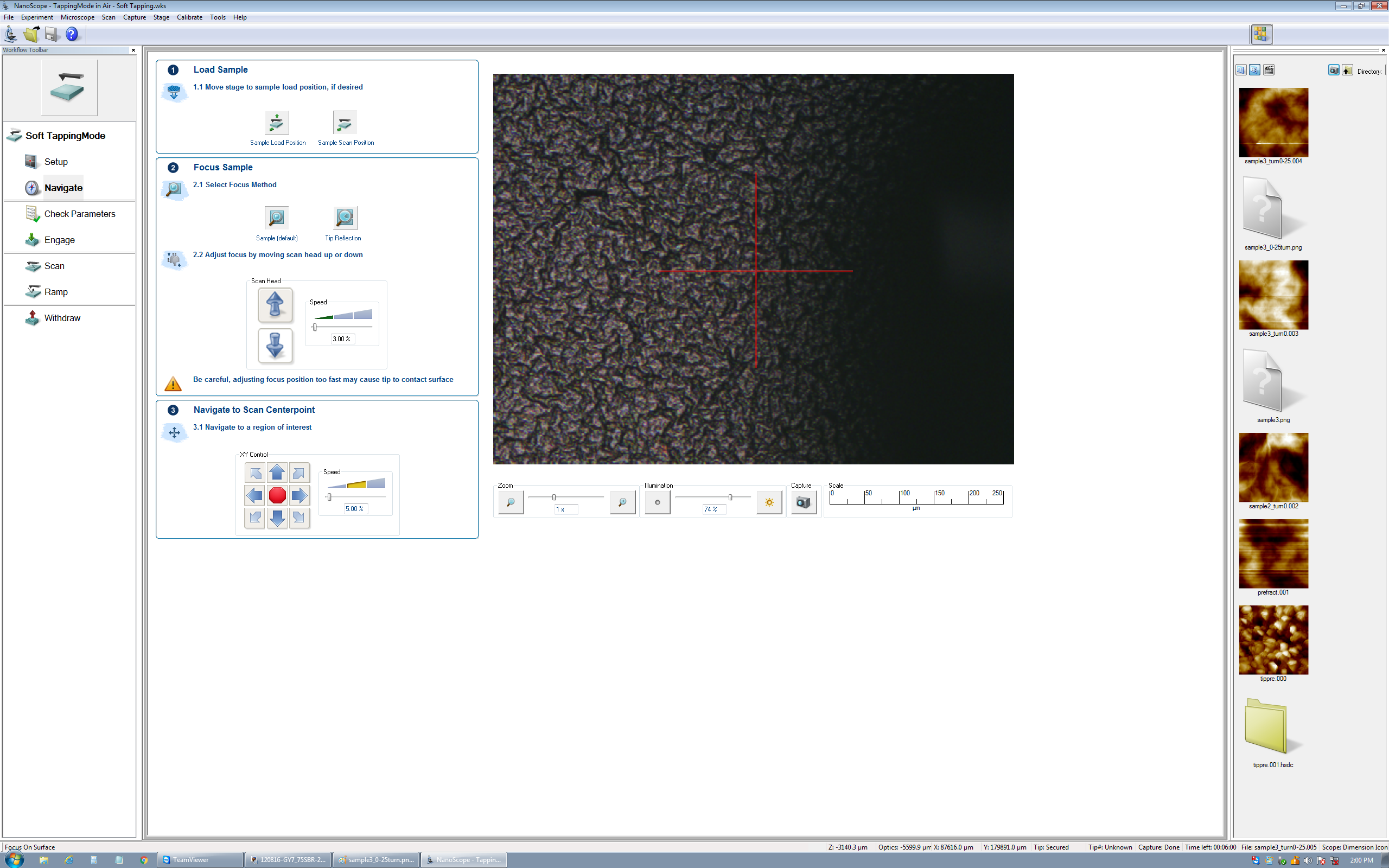

Supplement: S1 Data — (ZIP) [file pone.0197999.s002.zip › SI2_Data/Demonstration Problem 1/Exp2/sample3_0-5turn.png]

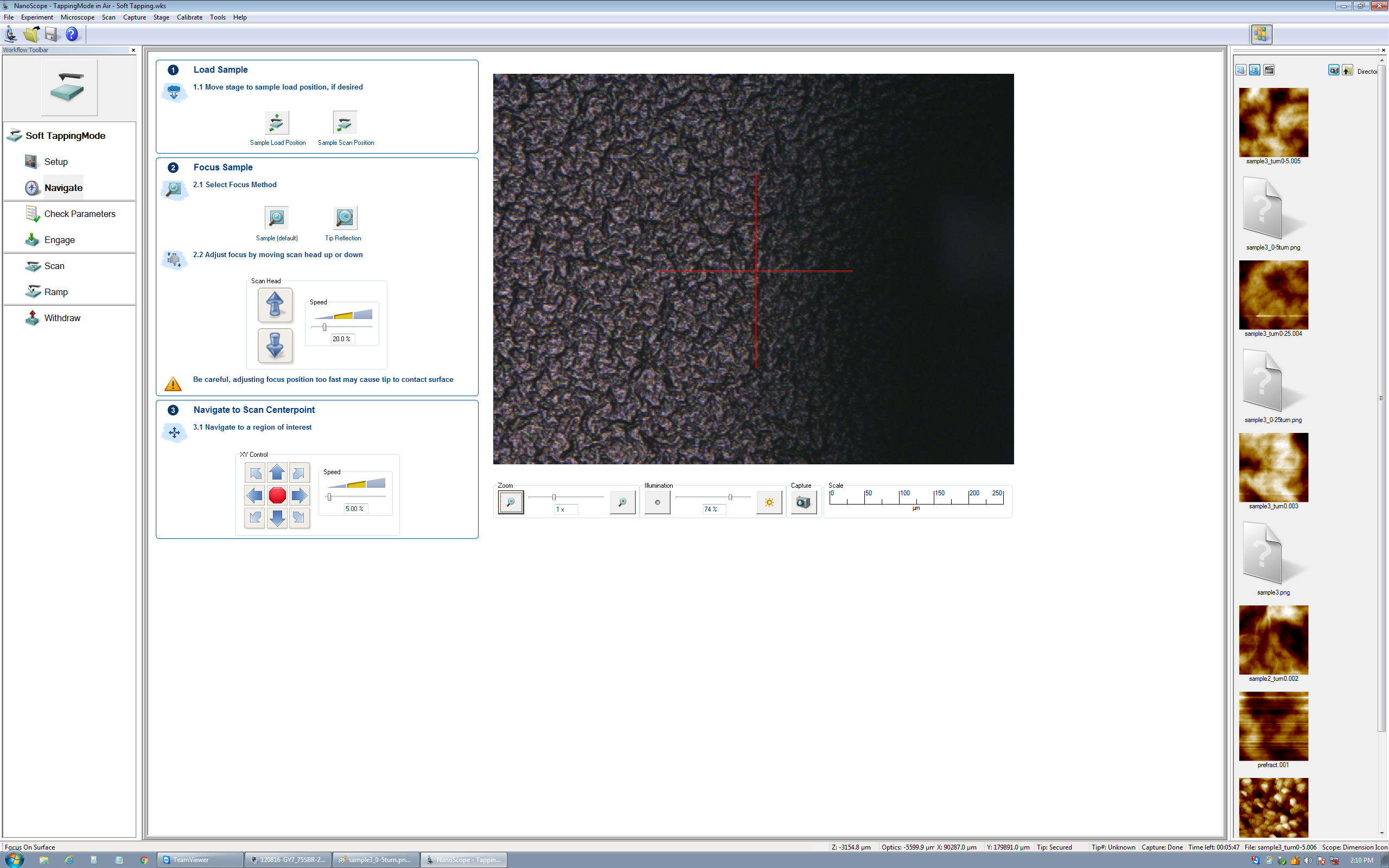

Supplement: S1 Data — (ZIP) [file pone.0197999.s002.zip › SI2_Data/Demonstration Problem 1/Exp2/sample3_1-5turn.png]

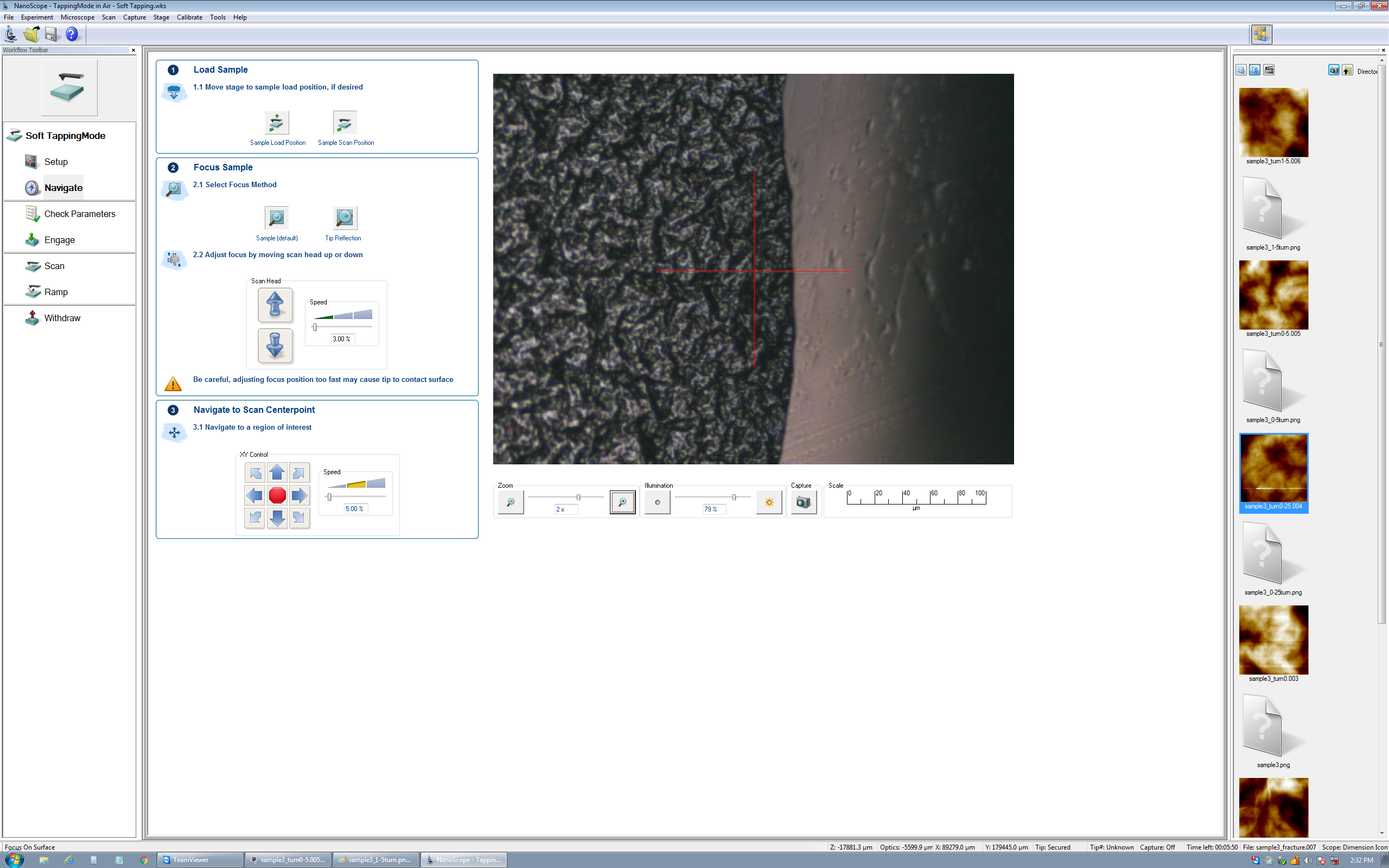

Supplement: S1 Data — (ZIP) [file pone.0197999.s002.zip › SI2_Data/Demonstration Problem 1/Exp2/sample3_fracture.png]

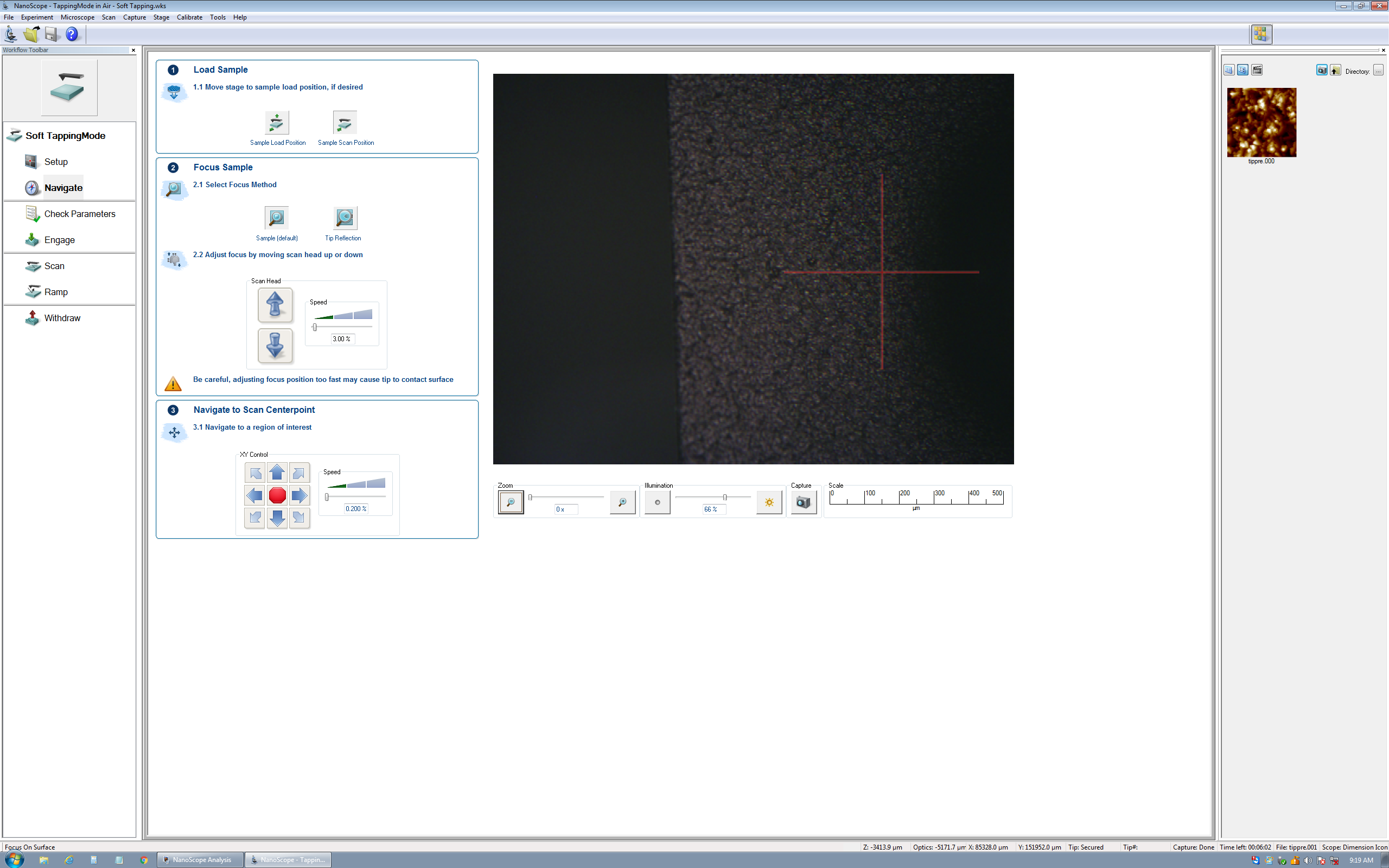

Supplement: S1 Data — (ZIP) [file pone.0197999.s002.zip › SI2_Data/Demonstration Problem 1/Exp3/Area1.png]

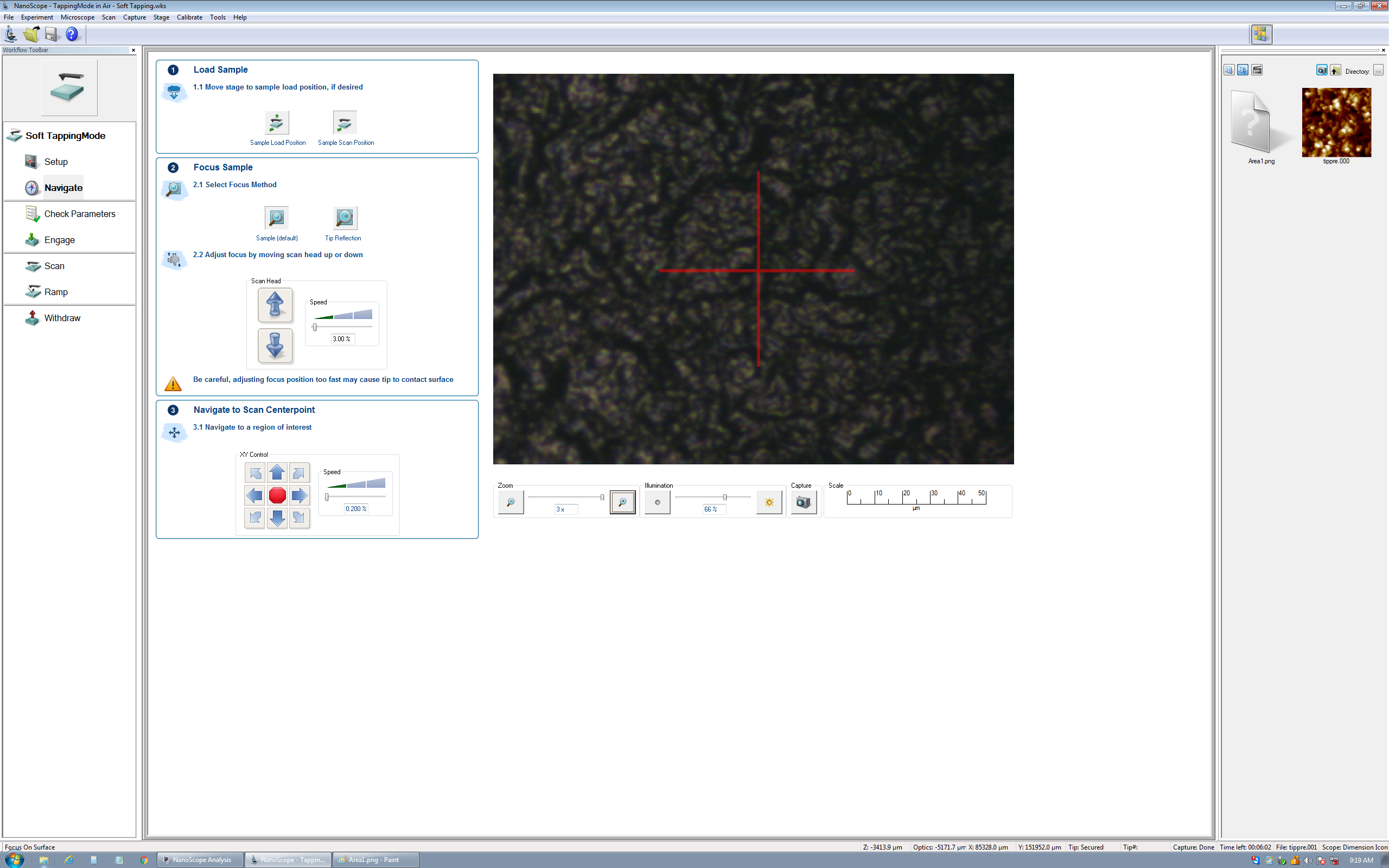

Supplement: S1 Data — (ZIP) [file pone.0197999.s002.zip › SI2_Data/Demonstration Problem 1/Exp3/Area1_zoom.png]

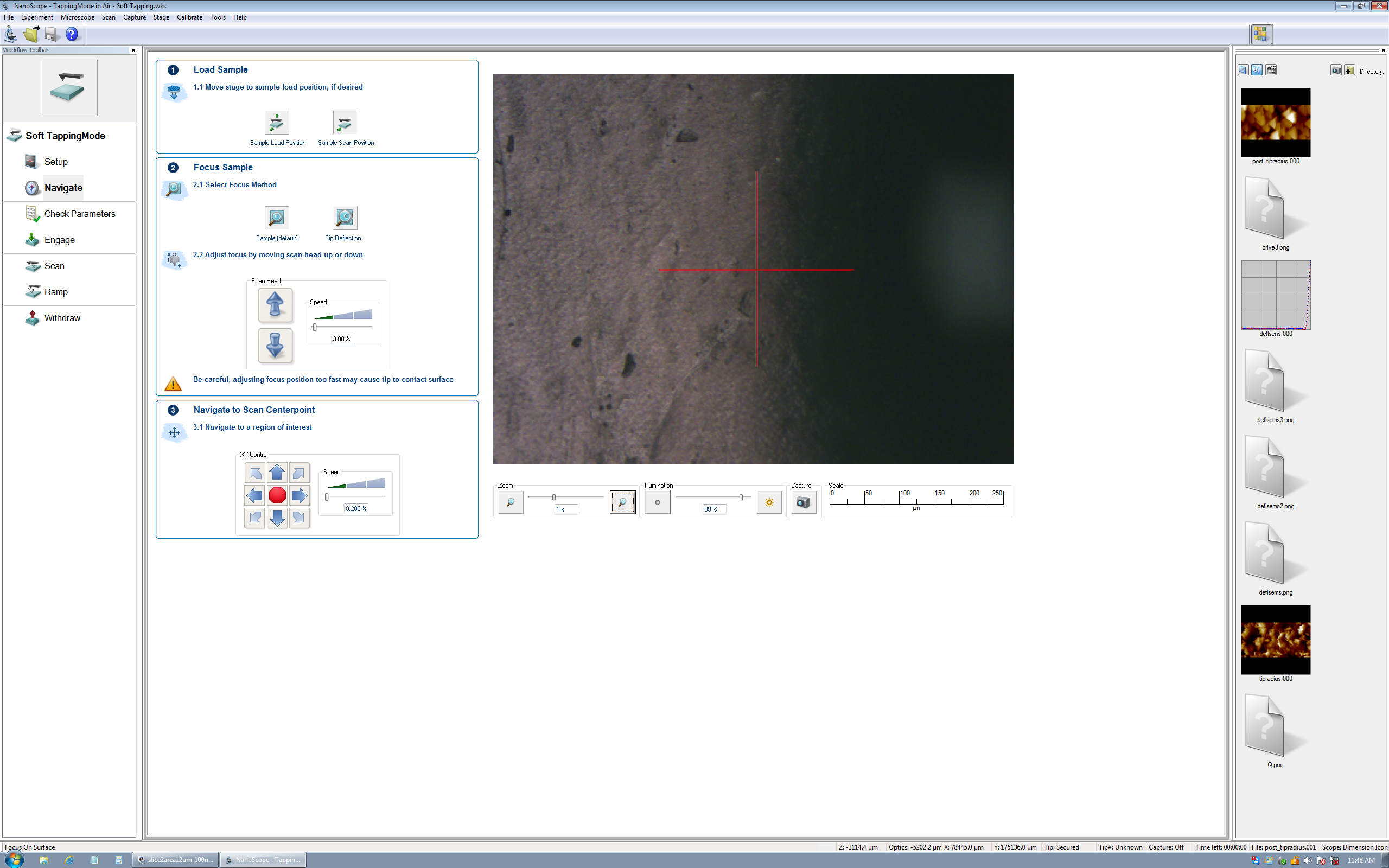

Supplement: S1 Data — (ZIP) [file pone.0197999.s002.zip › SI2_Data/Demonstration Problem 2/0-0025mgmL/Data/Scan area.png]

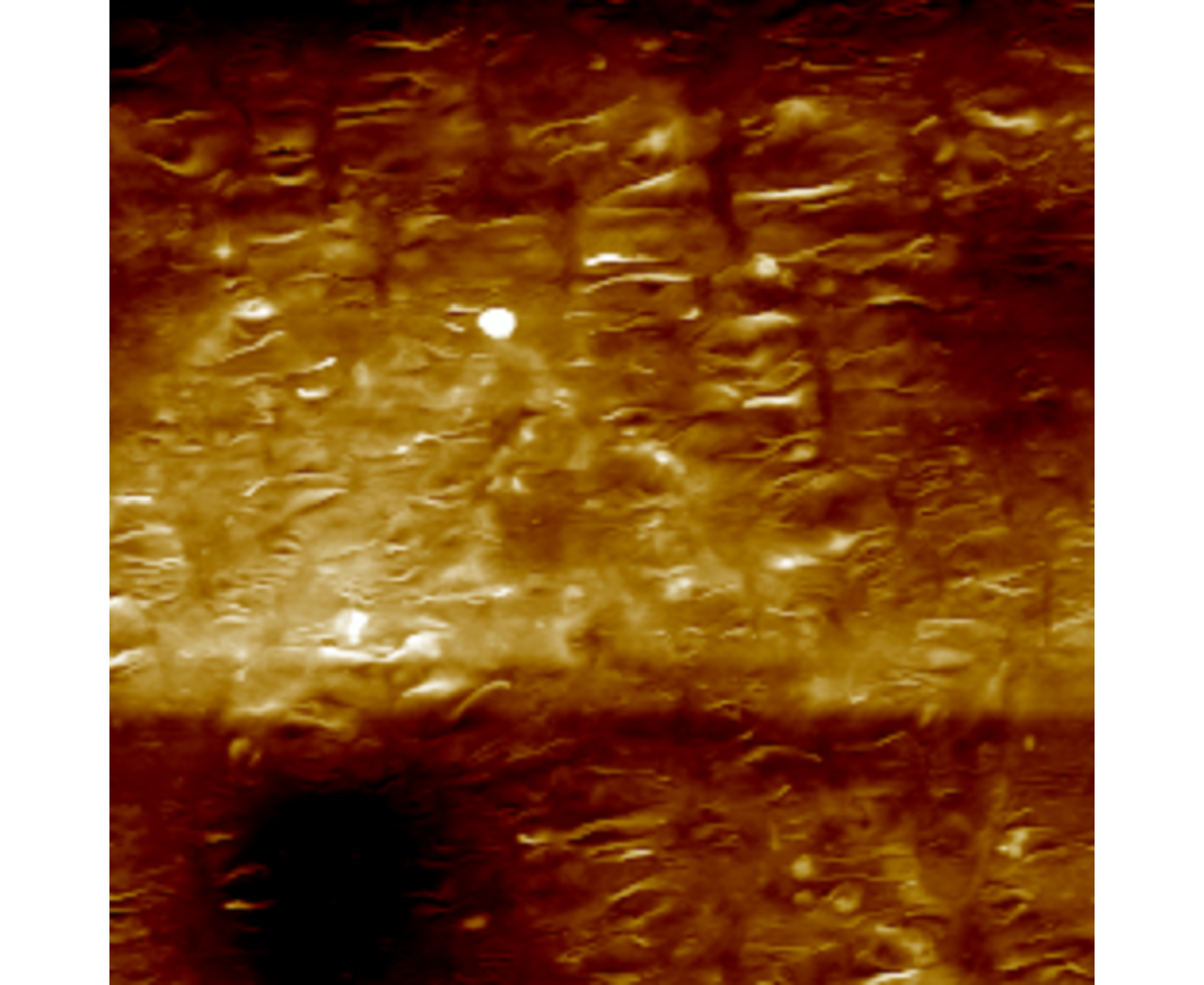

Supplement: S1 Data — (ZIP) [file pone.0197999.s002.zip › SI2_Data/Demonstration Problem 2/0-0025mgmL/Results/area1_eps246_turn_38_1.png]

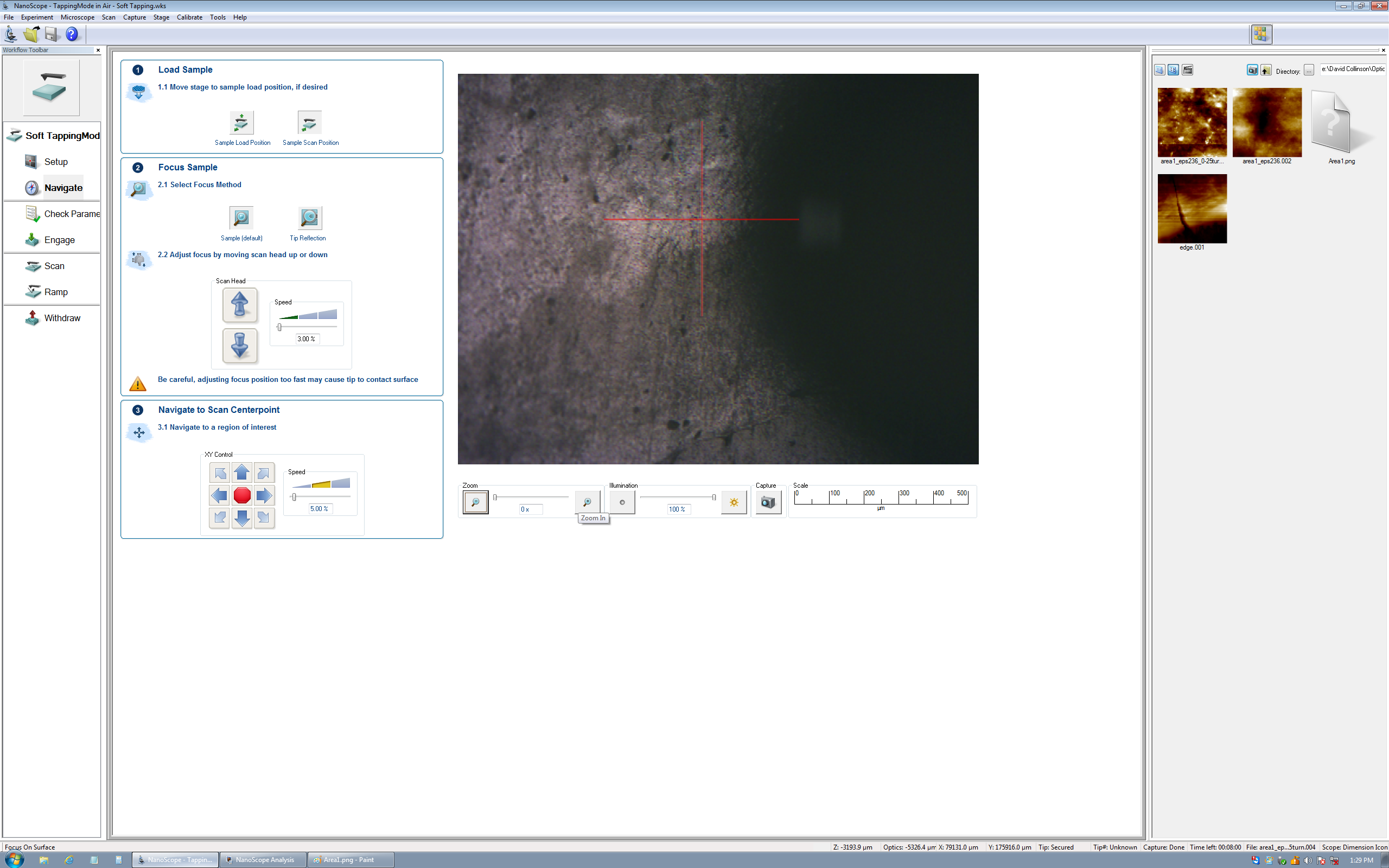

Supplement: S1 Data — (ZIP) [file pone.0197999.s002.zip › SI2_Data/Demonstration Problem 2/0-01mgmL/Data/Area1.png]

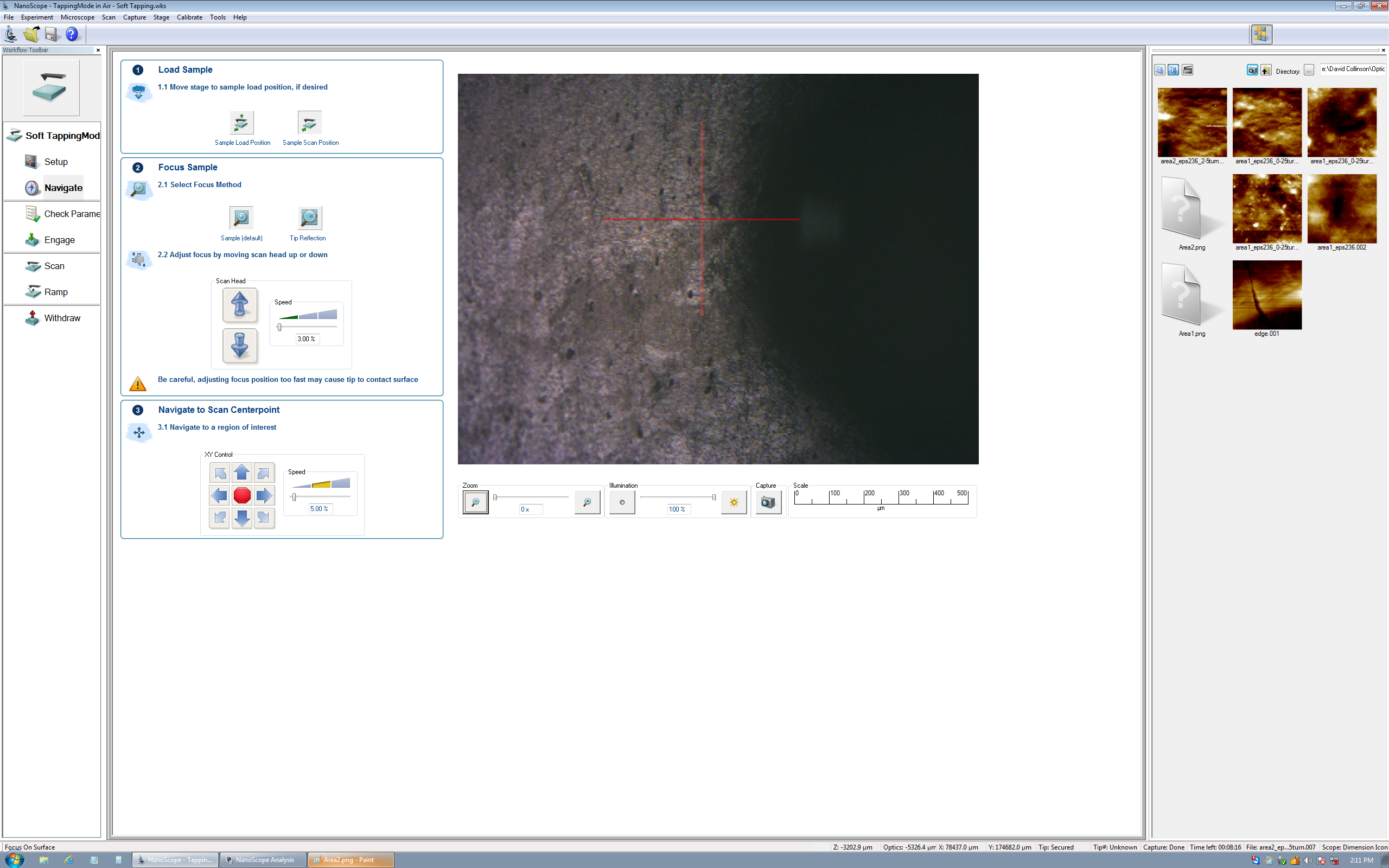

Supplement: S1 Data — (ZIP) [file pone.0197999.s002.zip › SI2_Data/Demonstration Problem 2/0-01mgmL/Data/Area3.png]
